# Reduction of Na^+^ within a {Mg_2_Na_2_} Assembly

**DOI:** 10.1002/anie.202213670

**Published:** 2022-12-08

**Authors:** Han‐Ying Liu, Samuel E. Neale, Michael S. Hill, Mary F. Mahon, Claire L. McMullin, Emma Richards

**Affiliations:** ^1^ Department of Chemistry University of Bath Claverton Down Bath BA2 7AY UK; ^2^ School of Chemistry Cardiff University Park Place Cardiff CF10 3AT UK

**Keywords:** Density Functional Theory, Magnesium, Main Group Chemistry, Redox Chemistry, Sodium

## Abstract

Ionic compounds containing sodium cations are notable for their stability and resistance to redox reactivity unless highly reducing electrical potentials are applied. Here we report that treatment of a low oxidation state {Mg_2_Na_2_} species with non‐reducible organic bases induces the spontaneous and completely selective extrusion of sodium metal and oxidation of the Mg^I^ centers to the more conventional Mg^II^ state. Although these processes are also characterized by a structural reorganisation of the initially chelated diamide spectator ligand, computational quantum chemical studies indicate that intramolecular electron transfer is abetted by the frontier molecular orbitals (HOMO/LUMO) of the {Mg_2_Na_2_} ensemble, which arise exclusively from the 3*s* valence atomic orbitals of the constituent sodium and magnesium atoms.

## Graphical abstract

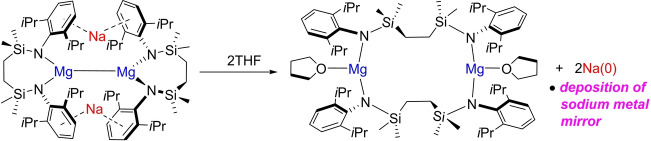

## Introduction

As the archetypal alkali metal, the chemistry of sodium is dominated by ionic compounds, NaX (where X=e.g. halide, hydroxide), comprising Na^+^ ions arising from the loss of its 3*s* ^1^ valence electron.^[1]^ Reflecting the stability of its compounds under “normal” (i.e. thermodynamic) conditions, both Davy's 1807 isolation of elemental sodium and the continuing method of its industrial‐scale production require electrolysis and the application of a highly negative standard potential (*E* ^0^=−2.71 V) to effect single electron reduction.^[2]^ Although the +1 oxidation state appears immutable, it was challenged by the discovery of homo‐ or heterometallic “sodides” (LM^+^Na^−^) (M=Na, K, Rb, Cs; L=crown or cryptand), the nature of which was widely explored by Dye and co‐workers from the 1970s onwards.[^3^, ^4^, ^5^, ^6^, ^7^, ^8^, ^9^, ^10^, ^11^, ^12^, ^13^, ^14^, ^15^, ^16^, ^17^, ^18^, ^19^, ^20^, ^21^, ^22^, ^23^, ^24^, ^25^, ^26^, ^27^, ^28^, ^29^, ^30^, ^31^, ^32^, ^33^] These fascinating ionic systems comprise isolated Na^−^ ions with a closed shell [Ne]3*s* ^2^ electronic configuration, albeit with a tendency to comproportionate back to Na^0^/M^0^. While this latter behaviour necessarily represents a process of intermetallic electron transfer, the resistance of the Na^+^ ion toward well‐defined molecular redox processes continues to prevail.

**Figure 1:**
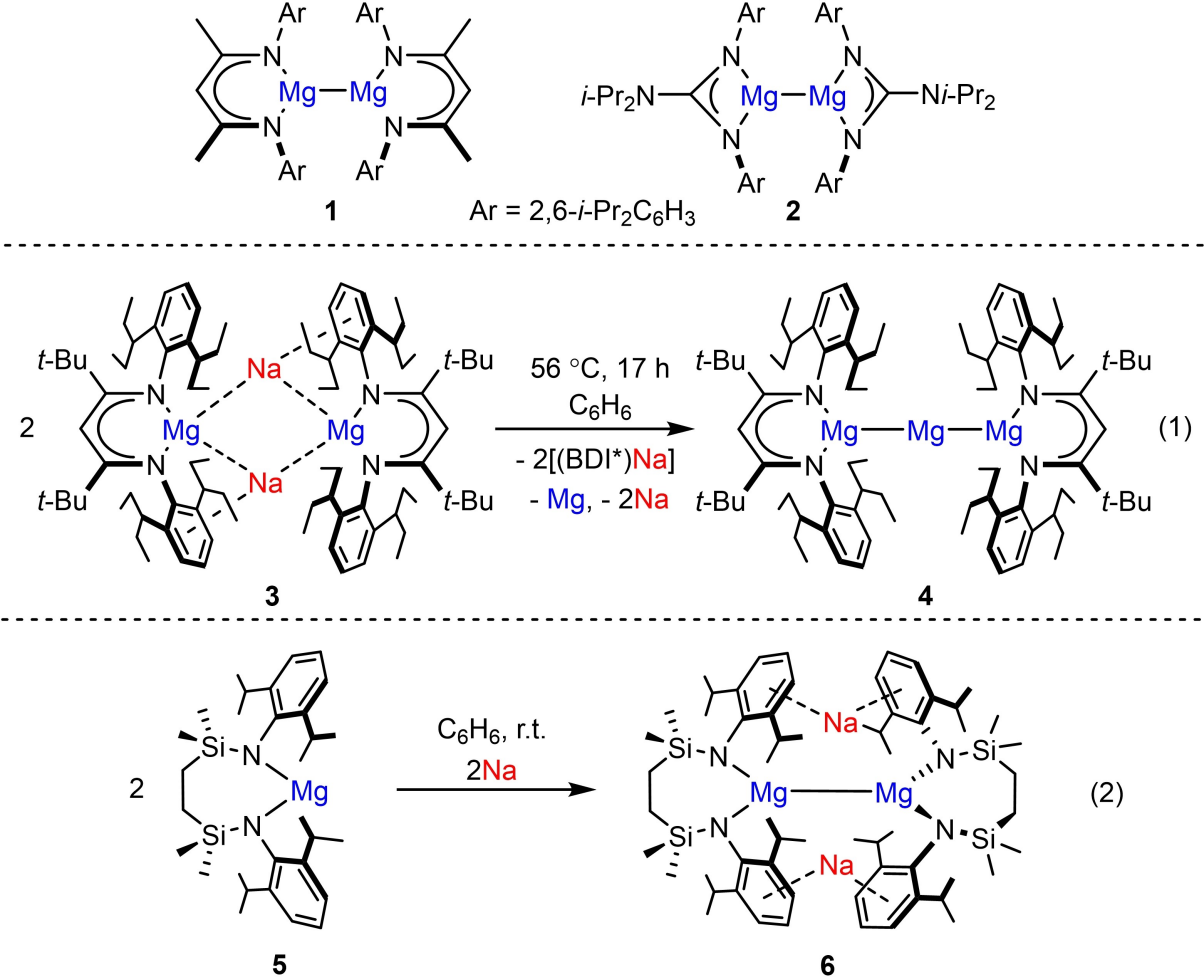
The structures of compounds **1**–**6** and the synthesis of compounds **4** [Eq. (1)] and **6** [Eq. (2)].

While closed shell Ae^2+^ ions also invariably result from oxidation of the alkaline earth elements of group 2 (Ae=Be, Mg, Ca, Sr, Ba), Jones and co‐workers’ isolation of Mg−Mg bonded β‐diketiminate (BDI) and guanidinate derivatives (e.g. **1** and **2**, Figure 1), in 2007 initiated a new era of kinetically stabilized molecular compounds containing a low oxidation state alkaline earth element.[^34^, ^35^, ^36^, ^37^, ^38^, ^39^, ^40^, ^41^] The highly reducing nature and solubility in common organic solvents of compounds **1** and **2** instigated a rapid development of Mg^I^ chemistry and, to a more limited extent, low oxidation state beryllium and calcium species during the subsequent 15 years.[^42^, ^43^, ^44^, ^45^, ^46^] Synthetic access to these derivatives is typically achieved by sodium or potassium metal reduction of an Ae^II^ halide reagent, an approach that also recently provided the unique Mg^0^ species, [(BDI*)MgNa]_2_ (**3**; BDI*=HC{C(*t*‐Bu)N(DiPeP)}_2_; DiPeP=2,6‐(3‐pentyl)‐phenyl), which exploits the extreme kinetic protection provided by the BDI* anion.^[47]^ Compound **3** was sufficiently stable to allow an initial assessment of its highly reducing reactivity and behaviour as a magnesium‐centered nucleophile. It was reported to decompose, however, either to unidentified species in the presence of ethereal bases or in benzene solution at moderately elevated temperatures [Figure 1; Eq. (1)]. This latter process provided [(BDI*)Na] and a striking tri‐magnesium species, [(BDI*)MgMgMg(BDI*)] (**4**), which was isolated in 9 % yield by mechanical separation from the mixture of compounds and identified by X‐ray diffraction analysis. The formation of **4** occurred simultaneously with the deposition of a metallic mirror of Na^0^ and Mg^0^ in which the metals were alloyed in a respective 2 : 1 ratio. Although the complexity of its decomposition products may militate against a more precise identification of the mode of electron transfer in **3**, the appearance of metallic magnesium and (BDI*)Na was attributed to the extrusion of the Mg^0^ centers from **3**. Similarly, the generation of elemental sodium and the mixed oxidation state {Mg^I^−Mg^0^−Mg^I^} unit of **4** was suggested to have plausibly arisen by formal reduction of Na^+^ by Mg^0^.^[47]^

The apparent significance of the Na‐arene interactions to the structural integrity of compound **3** prompted us to synthesize the topologically‐related but electronically contrasting species, [{SiN^Dipp^}MgNa]_2_ (**6**; [{SiN^Dipp^}={CH_2_SiMe_2_N(Dipp)}_2_; Dipp=2,6‐*i‐*Pr_2_C_6_H_3_)].^[48]^ Compound **6** was prepared by reduction of the magnesium(II) precursor (**5**) [Figure 1; Eq. (2)], the disilazide {SiN^Dipp^} ligand of which, in contrast to the {Mg^0^_2_Na^I^_2_} unit of **3**, attributes formal oxidation states of Na^I^ and Mg^I^ to the dissimilar metal centers. Although its resultant Mg^I^−Mg^I^ interaction is significantly elongated [ca. 3.21 Å] in comparison to those observed in Mg^I^ derivatives such as **1** and **2** [ca. 2.81 Å], quantum theory of atoms in molecules (QTAIM) analysis of **6** revealed a bond critical point (BCP; *ρ*=0.0194) consistent with the maintenance of a covalent Mg−Mg interaction. Two further bond paths were located between the Na cations and the Mg−Mg BCP itself. While weak (*ρ*=0.0034), these were estimated by perturbation energy analysis to represent an overall σ‐donation strength between the Mg−Mg bond and each Na^+^ cation of Δ*E* ^(2)^≈25 kcal mol^−1^. While its reactivity with CO highlighted that **6** is a potent two‐electron reductant in which the Na and Mg centers operate in harness, this initial computational assay hinted that the {Na_2_Mg_2_} unit is better considered as a contiguous ensemble rather than as a series of independent s‐block centers. In the current study, we provide an experimental and theoretical examination of this supposition, which demonstrates that perturbation of the electronic structure of **6** by its treatment with a range of non‐reducible neutral or anionic nucleophiles induces a counterintuitive, but facile and molecularly‐defined, Na^I^→Na^0^ redox process.

## Results and Discussion

**Figure 2:**
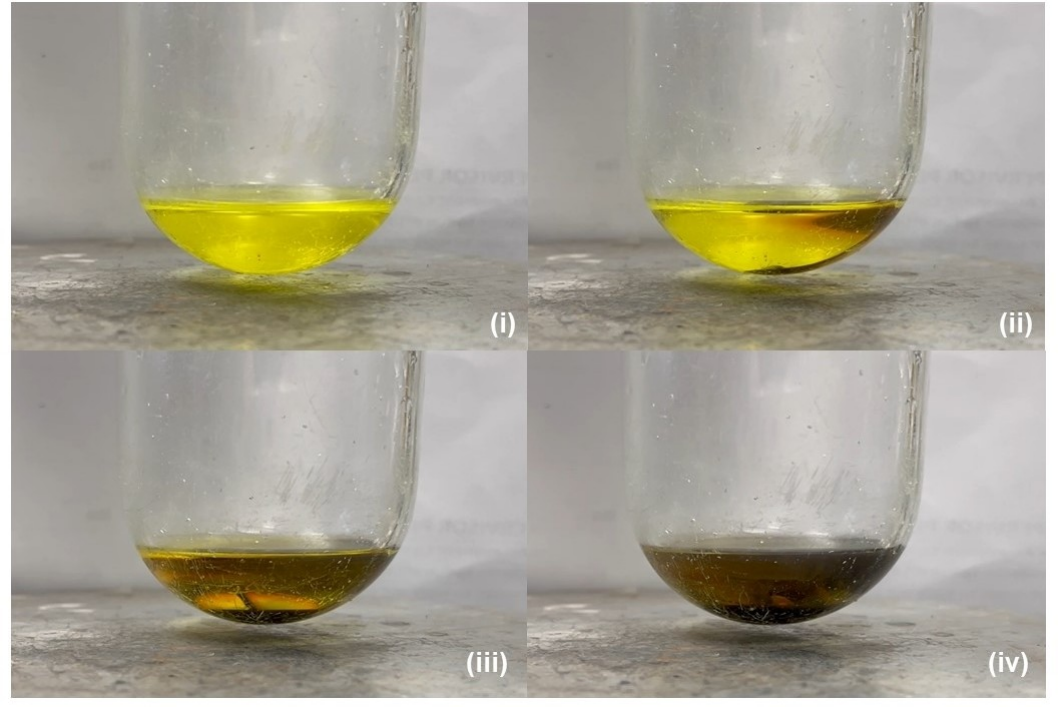
Photographs of a benzene solution of compound **6** (i) prior to, (ii) 5 seconds, (iii) 15 seconds and (iv) 30 seconds after the addition of a drop of THF, highlighting the formation of metallic sodium.

It was noted during the early stages of our study that bright yellow arene solutions of **6** did not tolerate even trace quantities of basic co‐solvents. Addition of a drop of THF, for example, induced the initial formation of a red/purple coloration and a subsequent bleaching of the solution over the course of several seconds, a process which occurred with the simultaneous deposition of a metallic mirror on the surface of the reaction vessel (Figure 2). Performance of this reaction in C_6_D_6_ with a two‐fold stoichiometry of THF, followed by filtration and analysis of the filtrate by ^1^H and ^13^C NMR spectroscopy, evidenced the selective formation of a single new C_2_‐symmetric compound (**7**), which was assessed to contain two molecules of coordinated ether by relative integration (4*H*) of the THF methylene ^1^H NMR resonances at *δ* 2.33 and 1.26 ppm. Consistent with the operation of a completely selective Na^I^→Na^0^ redox process, quantitative analysis by ICP‐OES demonstrated that the metallic mirror consisted solely (and of the entirety) of the sodium content of **6** employed in the reaction. Furthermore, the fate of the constituent magnesium of **6** was revealed by crystallization of compound **7**, which was isolated as colorless single crystals from the initial filtrate in high yield (83 %).

**Figure 3:**
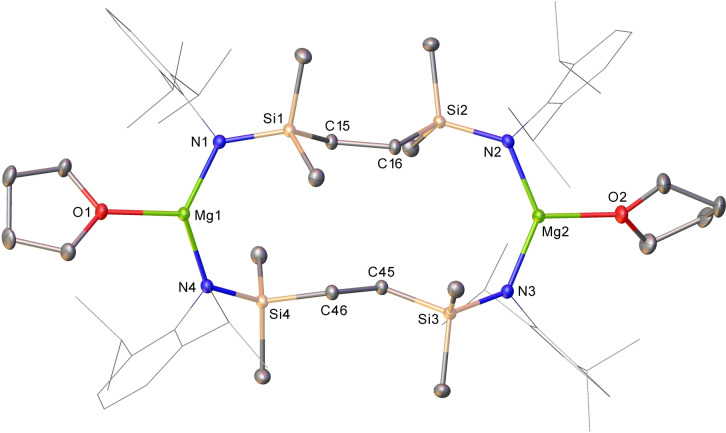
Displacement ellipsoid (30 % probability) plot of compound **7**. For clarity, hydrogen atoms, the second components of disordered atoms and solvent (benzene) have been omitted. Dipp substituents are shown as wireframe. Selected bond lengths [Å] and angles [°]: Mg1−O1 2.0200(9), Mg1−N1 1.9780(11), Mg1−N4 1.9795(11), Mg2−O2 2.0311(10), Mg2−N2 1.9651(11), Mg2−N3 1.9671(11), N1−Mg1−O1 111.16(4), N1−Mg1−N4 136.66(4), N4−Mg1−O1 112.10(4), N2−Mg2−O2 111.89(4), N2−Mg2−N3 134.52(4), N3−Mg2−O2 113.59(4).

The resultant X‐ray analysis (Figure 3) revealed that the extrusion of elemental sodium from **6** occurs through the oxidation of its {Mg^I^−Mg^I^} unit and ring opening of the 7‐membered disilazide chelate structures, which now adopt alternative {Mg‐μ‐κ^1^‐*N*,μ‐κ^1^‐*N*′‐Mg′} bridging modes between the magnesium centers to provide a 14‐atom macrocycle. The 3‐coordinate Mg1 and Mg2 are each further ligated by a single molecule of THF, while the relevant Mg−N [Mg1−N1 1.9780(11), Mg1−N4 1.9795(11), Mg2−N2 1.9651(11), Mg2−N3 1.9671(11) Å] and Mg−O [Mg1−O1 2.0200(9), Mg2−O2 2.0311(10) Å] bond lengths are consistent with an assignment of the Mg^II^ oxidation state to both atoms. Although it cannot be ascertained with any level of confidence whether the disilazide ligands of **7** originate from a single parent molecule of **6**, the atomic specificity of this process convincingly advocates the reaction stoichiometry shown in Equation 3.

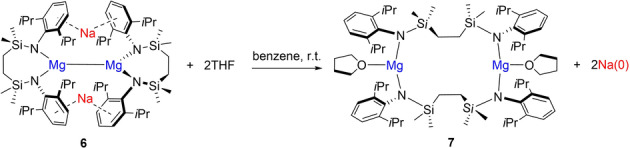




**Scheme 1:**
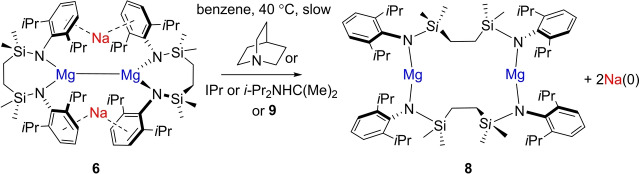
Synthetic routes to compound **8**.

**Figure 4:**
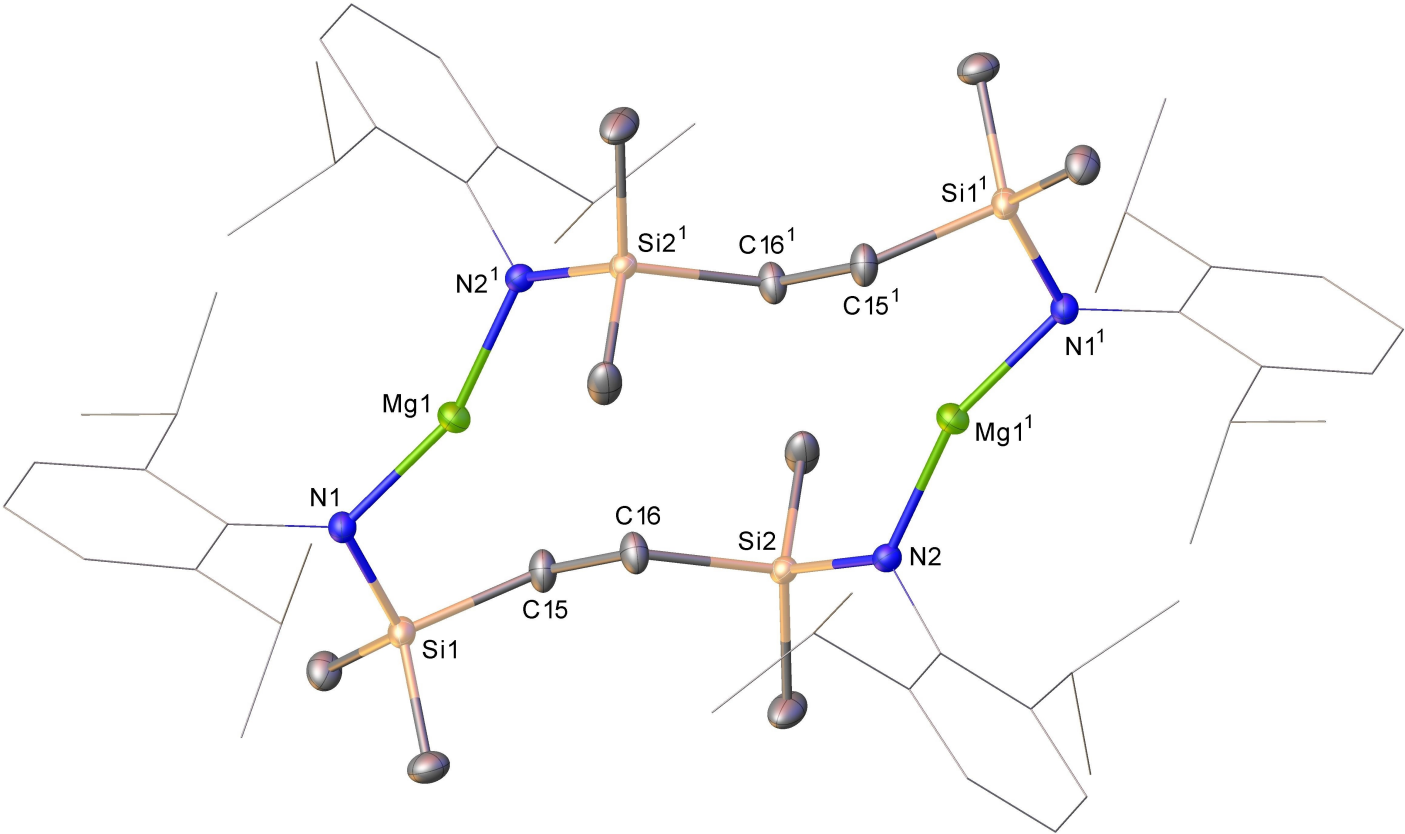
Displacement ellipsoid (30 % probability) plot of **8**. For clarity, hydrogen atoms omitted. Dipp substituents are shown as wireframe. Selected bond lengths [Å] and angles [°]: Mg1−N1 1.9207(12), Mg1−N2 1.9360(11), N1−Mg1−N2^1^ 160.17(5). Symmetry elements to generate symmetry‐related atoms, (^1^) 1−*x*, 1−*y*, 1−*z*.

Intrigued by this behavior, compound **6** was treated with two molar equivalents of the charge neutral, but more sterically encumbered, *N‐* and *C*‐donor bases, quinuclidine, 1,3‐di‐*iso*propyl‐4,5‐dimethyl‐2‐ylidene (*i*‐Pr_2_NHC(Me)_2_) and 1,3‐bis(2,6‐di‐*iso*propylphenyl)‐2‐ylidene (IPr) in d_6_‐benzene. Although no discernible changes were observed by ^1^H NMR spectroscopy, heating of these reactions to 40 °C for four weeks resulted in the similar formation of a grey powder. This process occurred alongside the deposition of subsequently insoluble colorless single crystals of compound **8** (Scheme 1 and Figure 4). Although definitive solution‐state characterization was limited by the low solubility of this species, mechanical separation and X‐ray diffraction analysis revealed, in all three cases, that **8** was a THF‐free variant of compound **7** containing 2‐coordinate magnesium (Figure 4). While its centrosymmetric structure necessitates no further comment, the isolation of **8** indicates that a similar process of sodium metal extrusion is induced even if the coordination of the basic molecule to magnesium is precluded by its steric demands.

**Figure 5:**
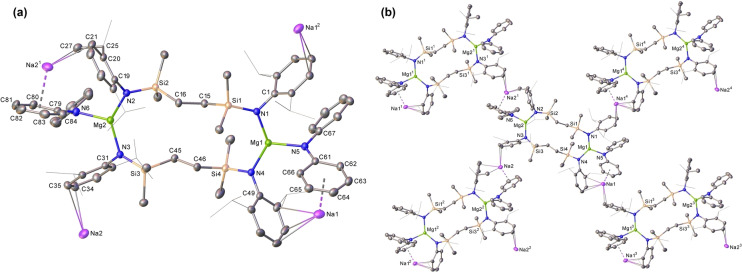
a) Displacement ellipsoid (30 % probability) plot of the asymmetric unit of compound **10** and b) the 2‐dimensional polymeric framework adopted by compound **10**. For clarity, hydrogen atoms, the second components of disordered atoms and solvent (benzene) have been omitted. Dipp isopropyl substituents are shown as wireframe. Selected bond lengths [Å] and angles [°]: Mg1−N1 2.0142(18), Mg1−N4 2.0130(19), Mg1−N5 2.0379(19), Mg2−N2 2.0057(19), Mg2−N3 2.0194(19), Mg2−N6 2.0341(19), N1−Mg1−N5 112.31(8), N4−Mg1−N1 133.02(8), N4−Mg1−N5 114.40(8), N2−Mg2−N3 133.16(8), N2−Mg2−N6 112.57(8), N3−Mg2−N6 114.05(8). Symmetry elements to generate symmetry‐related atoms, (^1^) 3/2−*x*, 1/2+*y*, 1/2−*z*; (^2^) 3/2−*x*, 1/2+*y*, 3/2−*z*; (^3^) 3/2−*x*, −1/2+*y*, 1/2−*z*; (^4^) 3/2−*x*, −1/2+*y*, 3/2−*z*.

Consistent with this supposition, reaction of **6** with the sterically encumbered, but formally charged, potassium alumanyl nucleophile, [{SiN^Dipp^}AlK]_2_ (**9**), provided similarly slow deposition at 40 °C of metallic sodium and single crystals of compound **8** (Scheme 1), which were identified by comparison of its unit cell parameters. Although the rate of a further reaction between **6** and two molar equivalents of the less bulky, but similarly charged, *N‐*donor nucleophile NaNPh_2_ was also limited by the low solubility of the amide reagent, heating to 40 °C over the course of 3 days was sufficient to induce the formation of sodium metal as a grey powder and a colorless solution, which was observed to comprise a single predominant product (**10**) by ^1^H and ^13^C NMR spectroscopy. Compound **10** was isolated by removal of volatiles, washing with hexane and crystallization from hot benzene, which provided colorless single crystals suitable for X‐ray diffraction analysis (Figure 5).

Like that of **7**, the asymmetric unit of **10** is based around a 14‐center macrocycle in which both Mg1 and Mg2 are 3‐coordinate. In this case, however, both magnesium atoms comprise magnesiate units resulting from *N‐*coordination of a formally anionic diphenylamide residue. Charge balance is maintained by incorporation of two sodium cations, Na1 and Na2, which reside within coordination environments defined by the aromatic substituents of the diphenylamide and *N‐*Dipp silylanilide ligands. Na1 is bound primarily by intramolecular chelation resulting from η^6^‐ and η^3^‐coordination of the N5‐containing diphenylamide and the N4*‐*bound Dipp substituents, respectively. Whereas encapsulation of Na1 is completed by a further close, but intermolecular, η^2^‐contact with an adjacent Mg2‐bound *N‐*Dipp substituent, Na2 is coordinated by a sequence of entirely intermolecular η^2^‐Dipp‐*N* and η^3^‐Ph‐*N* interactions such that the individual asymmetric units tessellate as a 2‐dimensional supramolecular framework (Figure 5b). Although it is not possible to ascertain the origin of the sodium cations in **10**, the specificity of the sodium metal extrusion observed during the formation of compound **7**, suggests that the balance of probability lies with the operation of an analogous process involving the reductive elimination of the sodium cations introduced as the heterobimetallic starting material (**6**).

As a further assessment of this behavior, benzene solutions of compound **6** were judged to be EPR silent, albeit such samples were consistently observed to contain variable quantities of unidentifiable paramagnetic impurities. A further reaction performed by addition of THF to a frozen solution of **6** within the cavity of a CW EPR spectrometer and then warmed to the melting point of the solvent also provided no definitive evidence for the production of observable radical intermediates. Development of the sodium mirror, however, was accompanied by the appearance of a broad signal (*g*_iso_=2.0068), which persisted after completion of the reaction. While sodium atoms trapped in noble gas (Ar, Kr, Xe) or hydrocarbon matrices have been observed to provide well resolved spectra displaying the expected ^23^Na (*I*=3/2) couplings,^[49]^ broad features obtained at *g*≈2 have been ascribed to the formation of the particulate metal after atomic diffusion.^[50]^ On this basis, we tentatively attribute the broad signals arising from these transformations of compound **6** to a similar phenomenon.

**Figure 6:**
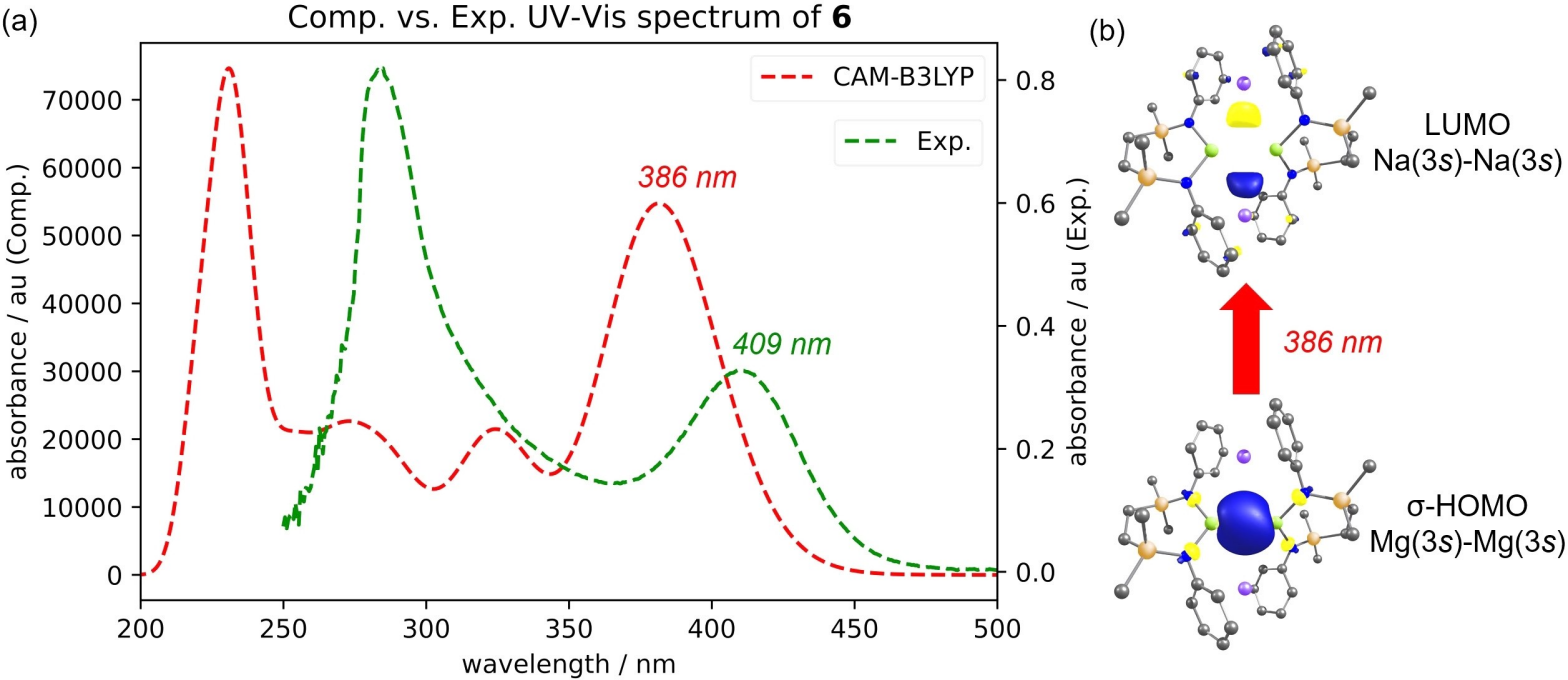
a) Experimental (benzene, 8.5×10^−8^ M) and TD‐DFT simulated (CAM‐B3LYP‐D3BJ,C_6_H_6_/def2‐TZVP//BP86/def2‐SVP—ORCA 5.0) UV/Vis spectra of compound **6**; b) the HOMO→LUMO transition attributed to the experimental absorption centered at 409 nm.

The yellow coloration of compound **6** in both solution and the solid state provides a notable feature for further investigation of its electronic structure. Accordingly, a UV/Vis spectrum (250–500 nm) was recorded of an 8.5×10^−8^ M solution in benzene (Figure 6a). Consistent with its physical appearance, the lowest energy electronic transition presents as a moderately intense absorption at 409 nm, while a more intense band with a discernible shoulder appears at 285 nm. TD‐DFT calculations were performed using the CAM‐B3LYP functional to simulate the spectrum and to provide an indication of the nature of the observed absorptions (see the ESI for full details). Although the absorption maxima displayed an appreciable blue shift of ca. 20–40 nm for the individual transitions, these methods provided a reasonable qualitative correspondence with the experimental data (Figure 6a). On this basis, the lowest energy excitation is attributed to a HOMO→LUMO transition between the Mg−Mg σ‐bond arising from overlap of the magnesium 3*s* wavefunctions and an MO represented by an out of phase combination of the sodium 3*s* atomic orbitals (Figure 6b). This observation is reminiscent of our previous examination of the {Mg_2_Na_2_} core using QTAIM and NBO methods and is, again, indicative of a significant degree of cooperativity between the alkali and alkaline earth element components. Moreover, the calculations indicate that the yellow color is largely governed by this HOMO→LUMO excitation in the visible blue light region. The next highest excitation characterized is a HOMO→LUMO+1 excitation, in which the LUMO+1 features predominant π*‐antibonding character with respect to each Dipp group that is engaged via cation‐π interactions with sodium. Hence, the TD‐DFT calculations are also consistent with a degree of involvement of the Dipp groups in the visible light absorption behaviour of **6**.

**Figure 7:**
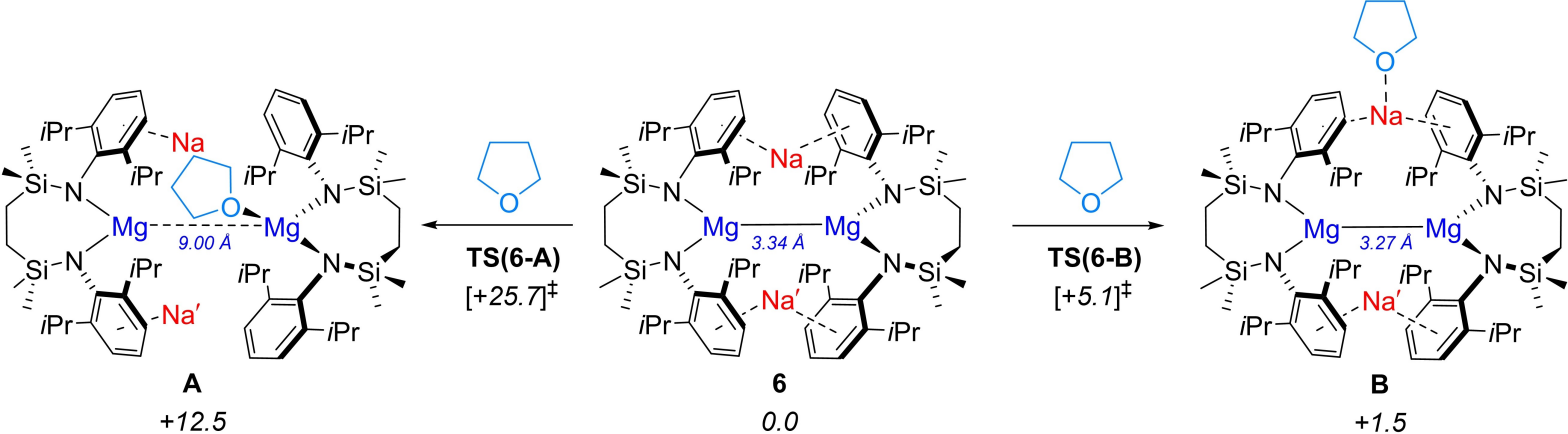
DFT‐computed (BP86‐D3BJ/BS2(C_6_H_6_)//BP86/BS1) free energies in kcal mol^−1^ for THF coordination to **6** at either the Mg^I^ (left) or Na^I^ centers (right).

In the context of its transformation to compounds **7**, **8** and **10**, we hypothesise that this inferred communication between the formal {Mg^I^−Mg^I^} unit and the constituent sodium cations of **6** provides the necessary conduit for electron transfer and the notably facile Na^I^→Na^0^ redox events. To explore the reactivity of **6** and probe the formation of **7**, computational mechanistic studies were performed with DFT at the BP86‐D3BJ(C_6_H_6_)/BS2//BP86/BS1 level of theory (see the ESI for full details). Multiple scenarios were assessed, including the thermodynamics associated with the removal of one or both of the Na atoms, either as single Na^0^ atoms or Na^I^ cations (lone cation or solvated {Na(THF)_4_}^+^). The loss of Na^I^ from **6** (Δ*G*_bnz_=+23.0 kcal mol^−1^) was, despite still being endergonic, thermodynamically favoured over the removal of an Na^0^ atom (Δ*G*_bnz_=+35.9 kcal mol^−1^). To interrogate how the formation of **7** may be initiated, therefore, coordination of an initial single molecule of THF to **6** at both Mg^I^ (**A**) and Na^I^ (**B**) was computed (Figure 7).

**Figure 8:**
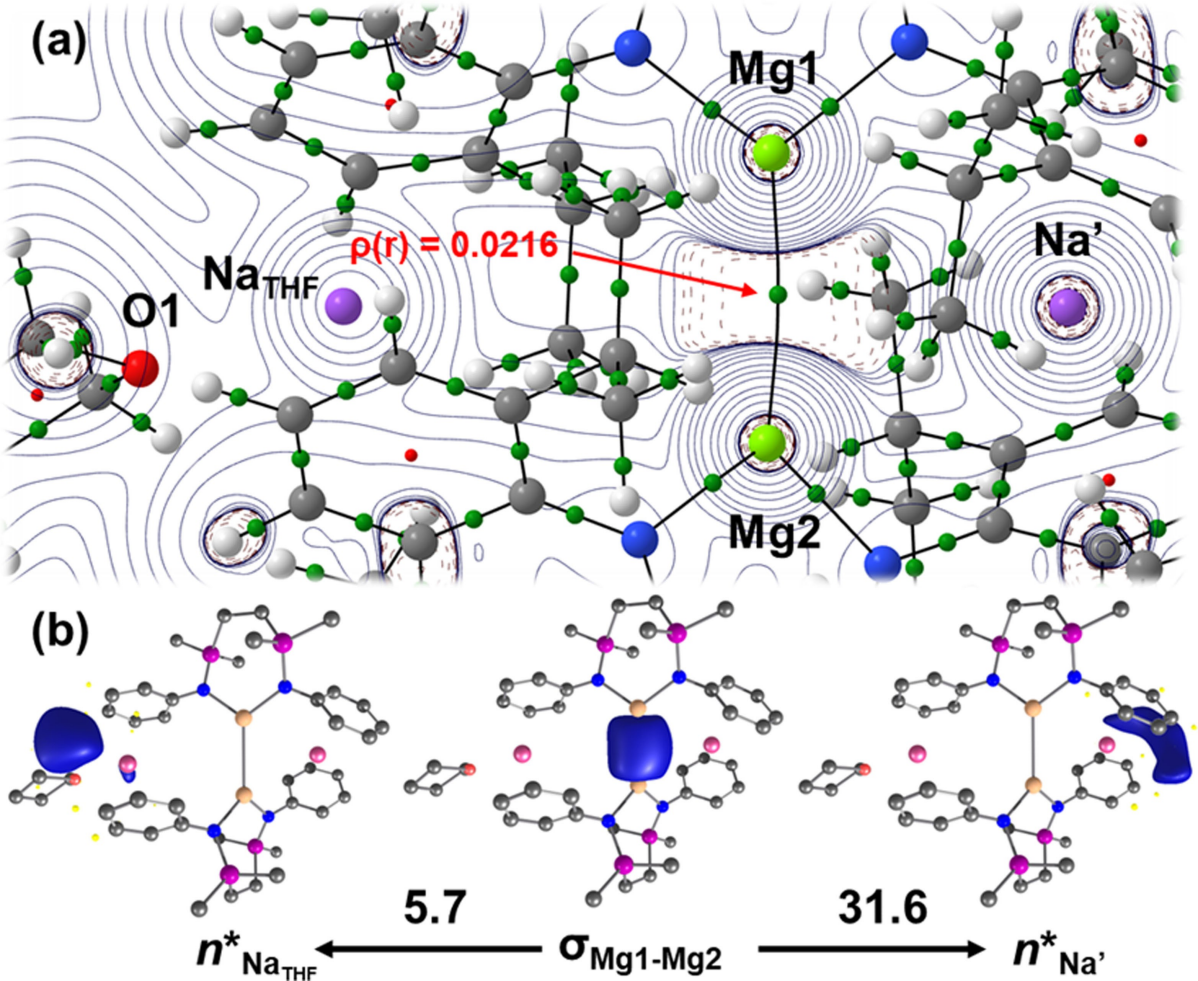
a) QTAIM Laplacian plot of **B**. b) The two sets of σ_MgMg_→n*_Na_ interactions in **B**, as identified by second‐order perturbation energy analysis of the Fock matrix in NBO basis, with the donor‐acceptor interaction energies, Δ*E* ^(2)^, quoted in kcal mol^−1^.

A significant kinetic and thermodynamic preference was identified towards THF coordination at the Na^I^ center, via **TS(6‐B)** (+5.1 kcal mol^−1^) to form **B** (+1.5 kcal mol^−1^), rather than coordination at Mg^I^ via **TS(6‐A)** (+25.7 kcal mol^−1^) (Figure 7).^[51]^ Moreover, the barrier to formation of **B** at +5.1 kcal mol^−1^ is qualitatively consistent with the room temperature observation of an immediate color change upon addition of THF to the benzene solution of **6** (Figure 2). Coordination of THF to Na^I^ induces a partial disengagement of the cation from the Dipp substituents, which perturbs the structure of the {Mg_2_Na_2_} tetrametallic core in **B** compared to **6** (Figure 7). Although this process incurs an elongation of the Mg⋅⋅⋅Na_THF_ contacts to 5.025 and 5.127 Å, the interaction of the remaining sodium (Na′) with the {Mg−Mg} unit is augmented, resulting in a contraction of the Mg⋅⋅⋅Na′ distances to 3.737 and 3.776 Å and a shortening of the Mg−Mg bond to 3.270 Å. QTAIM and NBO analyses were performed on **B** to probe the effects on this now perturbed tetrametallic core for comparison with the previously described data for **6**. The results of this study are outlined in Figure 8.

QTAIM analysis reveals that the Mg1−Mg2 bond in **B** has a larger density at the bond critical point (BCP) with respect to **6**, where *ρ*(*r*)=0.0216 (0.0194 in **6**), and more negative Laplacian (∇^2^ *ρ*(*r*)=−0.0155 for **B**, versus −0.0136 in **6**) and energy density values (where H(*r*)=−0.00412 in **B** vs. −0.00362 in **6**). These data are consistent with an augmentation of the Mg1−Mg2 covalent bond in **B** as a result of THF coordination to the Na_THF_ cation. Furthermore, the Mg1−Mg2 bond path now displays a mild curvature towards the Na′ center, reinforcing the inference that formation of the Na_THF_ unit has disrupted the previously characterized equivalence of the interactions between the Mg−Mg bond and Na cations in **6**.^[48]^ Analysis of BCPs of low density in **B** revealed a small, yet appreciable, Na′−Mg1 BCP that is primarily ionic in nature, where *ρ*(*r*)=0.005 and ∇^2^ *ρ*(*r*)=+0.0074. While the density at this BCP has, thus, increased from that characterized in **6** (*ρ*(*r*)=0.0034),^[48]^ no appreciable BCP between the Na_THF_ unit and the Mg−Mg bond could now be characterized. This is also supported by second‐order perturbation energy analysis of the Fock matrix in NBO basis, where two sets of σ_MgMg_→*n*_Na*_ interactions are captured (Figure 8b), with stronger σ‐donation toward Na′ over Na_THF_, and where Δ*E* ^(2)^=31.6 kcal mol^−1^ (*cf*. 25.4 kcal mol^−1^ in **6**).^[48]^ From this perspective, therefore, **B** is now better considered as comprising a cooperative {Mg_2_Na′} trimetallic subunit, wherein the shortening of the interatomic distances may facilitate a viable pathway for initiation of the redox chemistry observed upon addition of THF or other non‐reducible bases (i.e. formal Mg oxidation and Na reduction).

Together, these observations indicate that the thermodynamic stability of compound **6** is delicately poised. It is also notable that the formation of **8** is effectively the reverse of the process involved in synthesis of **6** [Figure 1; Eq. (2)], albeit the chelate structure of **5** is disrupted during Mg^I^ to Mg^II^ oxidation. Any further theoretical analysis of the formation of **7** is, thus, necessarily speculative and complicated by the observed macrocyclization of the disilazido magnesium chelate structure of **6** and the coordination of a molecule of THF to both magnesium centers. We have not yet observed any evidence, either thermally or under the stimulus of other reagents, for the direct conversion of compound **5** to compound **8**. The calculated free energies of both species, however, not only indicate that macrocycle formation is appreciably exergonic (Δ*G*=−12.9 kcal mol^−1^), but also highlight the significant stabilization provided to the structure of **8** by the computed dispersion correction, the absence of which maintains the equilibrium significantly toward the chelated structure of **5** (Table S14). In further mitigation of this hypothesis, and although the calculations do not take into account possible crystal packing effects, the optimized structure of **8** identifies close Mg⋅⋅⋅H_Cβ_ contacts (Mg⋅⋅⋅H_Cβ_=2.38–2.39 Å) that are expedited by the conformational flexibility of the macrocyclic structure. On this basis, therefore, it appears likely that related macrocyclization processes contribute significantly to the overall exothermicity of the transformation of **6** to compounds **7**, **8** and **10**. Furthermore, we have been able to assess the thermodynamic viability of the formation of both **7** and **8** and note the exergonic stability of both species to be −11.2 and −18.1 kcal mol^−1^, respectively (See Supporting Information for a presentation of the computational approaches adopted and further structural information).

## Conclusion

In summary, we have observed that treatment of a low oxidation state {Mg_2_Na_2_} assembly with basic molecules that are less prone to reduction than the sodium cation itself induces the complete extrusion of the elemental alkali metal. Although this process also results in a significant reorganisation of the initially chelating diamide supporting ligand, quantum chemical calculations indicate that the origin of this process may be traced to a perturbation of the electronic structure of the tetra‐metallic ensemble. We are continuing to further characterize, generalize and utilize this unusual *s*‐block element derived redox reactivity.

## Supporting Information

Full experimental and instrumental details, NMR, UV/Vis and EPR spectral data, details of the X‐ray analysis of compounds **7**, **8** and **10**,^[52]^ and the methods employed in the quantum chemical investigations of this chemistry are available in the Supporting Information to this article.

## Conflict of interest

The authors declare no conflict of interest.

1

## Supporting information

As a service to our authors and readers, this journal provides supporting information supplied by the authors. Such materials are peer reviewed and may be re‐organized for online delivery, but are not copy‐edited or typeset. Technical support issues arising from supporting information (other than missing files) should be addressed to the authors.

Supporting Information

Supporting Information

Supporting Information

Supporting Information

## Data Availability

The data that support the findings of this study are available in the Supporting Information of this article.
