# Supplementary material for: Reduction of Na+ within a {Mg2Na2} Assembly
Source: Angew Chem Int Ed Engl. 2022 Dec 8;62(3):e202213670. doi: 10.1002/anie.202213670 (PMC10107709; doi:10.1002/anie.202213670)
Supplement: Supplementary file 1 — Supporting Information [file ANIE-62-0-s002.pdf]

Angewandte Chemie
Eine Zeitschrift der Gesellschaft Deutscher Chemiker

Supporting Information

Reduction of Na^+ within a $\{Mg_2Na_2\}$ Assembly

H.-Y. Liu, S. E. Neale, M. S. Hill, M. F. Mahon, C. L. McMullin*, E. Richards*

1 Experimental and Supplementary Information

1.1. General information

Unless stated otherwise, all experiments were conducted using standard Schlenk line and/or glovebox techniques under an inert atmosphere of argon. NMR spectra were recorded with an Agilent ProPulse spectrometer (^{1}H at 500 MHz, ^{13}C at 126 MHz). The spectra are referenced relative to residual protio solvent resonances. Elemental analyses were performed at Elemental Microanalysis Ltd., Okehampton, Devon, UK. Solvents were dried by passage through a commercially available solvent purification system and stored under argon in ampoules over 4 Å molecular sieves. C_6D_6 was purchased from Sigma-Aldrich, dried over a potassium mirror before distilling and storage over molecular sieves. $[\{SiN^{Dipp}\}MgNa]_2$ (**6**),[1] $NaNPh_2$,[2] 1,3-diisopropyl-4,5-dimethyl-2-ylidene[3] and 1,3-bis(2,6-diisopropylphenyl)-2-ylidene[3] and $[\{(SiN^{Dipp})Al\}K]_2$[4] were prepared according to reported procedures. All other chemicals were purchased from Merck and used without further purification.

1.2. Synthetic procedures

Synthesis of [{SiN^{Dipp}}$_2$Mg$_2$-THF$_2$] (7)

In a J Young's tube, [{SiN^{Dipp}}MgNa]$_2$ (**6**, 21.6 mg, 0.02 mmol) was dissolved in 0.4 mL of d$_6$-benzene before the addition of tetrahydrofuran (3.2 μL, 2.8 mg, 0.04 mmol) *via* a micropipette to the bright yellow solution. Upon addition, the reaction mixture turned an immediate reddish purple as the tetrahydrofuran diffused into the solution. A metallic mirror was seen to deposit on the walls of the reaction vessel after a few seconds and the reaction mixture was observed to have completely decolourised within a few minutes. The reaction mixture was then filtered and the filtrate was kept at room temperature to afford **7** as colourless crystals, from which a single crystal suitable for X-ray diffraction was selected. Yield 19.6 mg, 83 %. Anal. Calc'd. for $C_{68}H_{116}Mg2N_4O_2Si_4$ (**7**, 1182.65): C, 69.06; H, 9.89; N, 4.74 %. Found: C, 68.69; H, 10.19; N, 4.30 %. ^{1}H NMR (500 MHz, 298 K, Benzene-*d*$_6$) δ 7.05 (d, *J* = 7.6 Hz, 8H, *m*-C$_6$*H*$_3$), 6.89 (t, *J* = 7.6 Hz, 4H, *p*-C$_6$*H*$_3$), 4.14 (sept, *J* = 6.9 Hz, 8H, C*H*Me$_2$), 2.33 (s br, 8H, OC*H*$_2$CH$_2$), 1.37 (d, *J* = 6.9 Hz, 24H, CH*Me*$_2$), 1.26 (s, 8H, SiC*H*$_2$), 1.09 (d, *J* = 6.9 Hz, 24H, CH*Me*$_2$), 0.69 (s br, 8H, OCH$_2$C*H*$_2$), 0.34 (s, 24H, Si*Me*$_2$). ^{13}C NMR (126 MHz, 298 K, Benzene-*d*$_6$) δ 152.7 (*i*-*C*$_6$H$_3$), 144.8 (*o*-*C*$_6$H$_3$), 123.5 (*m*-*C*$_6$H$_3$), 120.0 (*p*-*C*$_6$H$_3$), 69.4 (O*C*H$_2$CH$_2$) 27.4 (*C*HMe$_2$), 25.4 (CH*Me*$_2$), 25.2 (OCH$_2$*C*H$_2$), 25.0 (CH*Me*$_2$), 14.7 (SiCH$_2$), 1.9 (Si*Me*$_2$). Identification of the metallic mirror was conducted by ICP-OES analysis in Butterworth Lab, and it was confirmed to be Na metal; Anal. Calc'd: Na, 1001; Mg 0 mg/L; Found: Na, 975; Mg, <1 mg/L. (Details are available in section 1.5). Recorded footage of ehe rapid change of the benzene solution of **7** upon addition of (excess) tetrahydrofuran conducted in both a Young's NMR tube scale and a larger scale Schlenk flask is available in the electronic Supporting Information.

Figure S1. ^{1}H NMR (500 MHz, 298 K, d$_6$-benzene) spectrum of **7**.

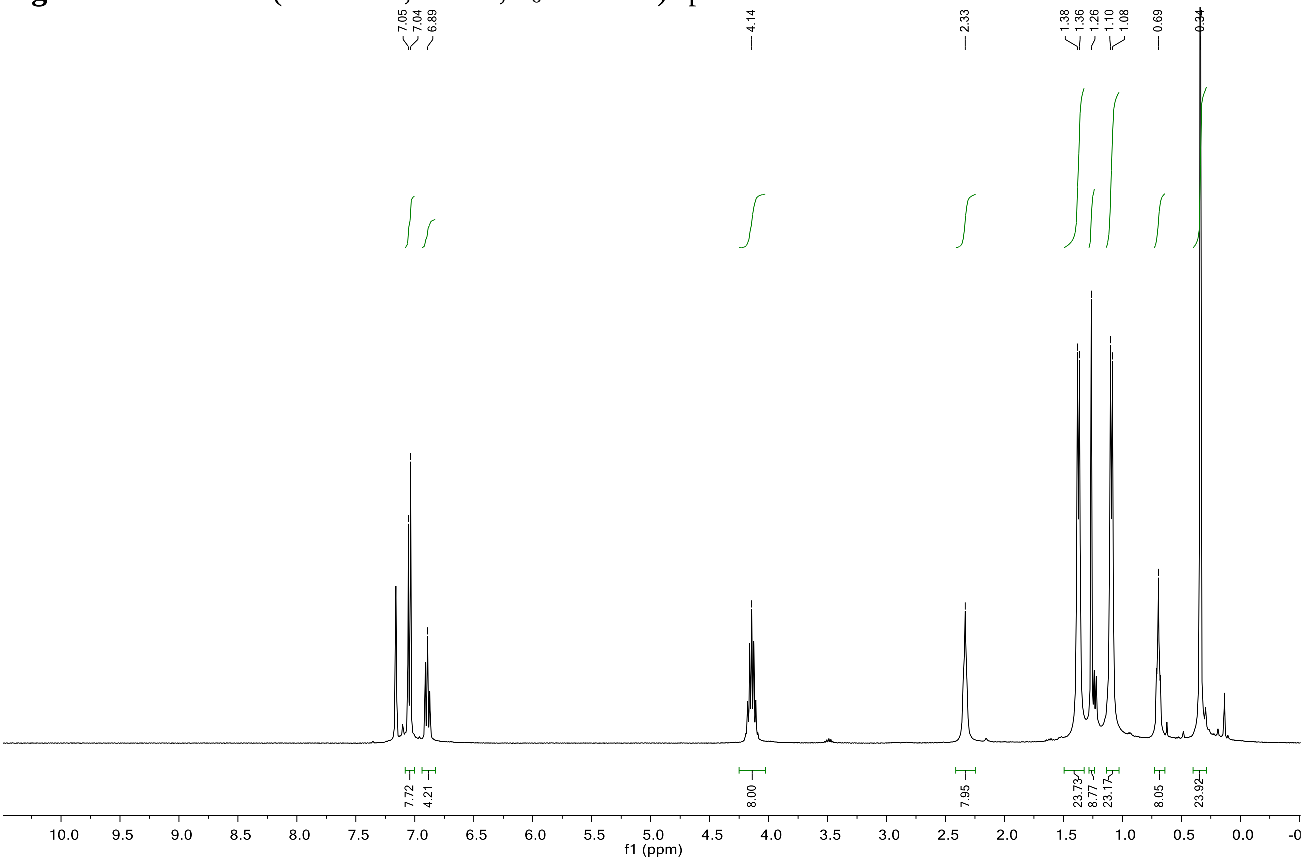

Figure S2. ^{13}C{^{1}H} NMR (126 MHz, 298 K, d$_6$-benzene) spectrum of **7**.

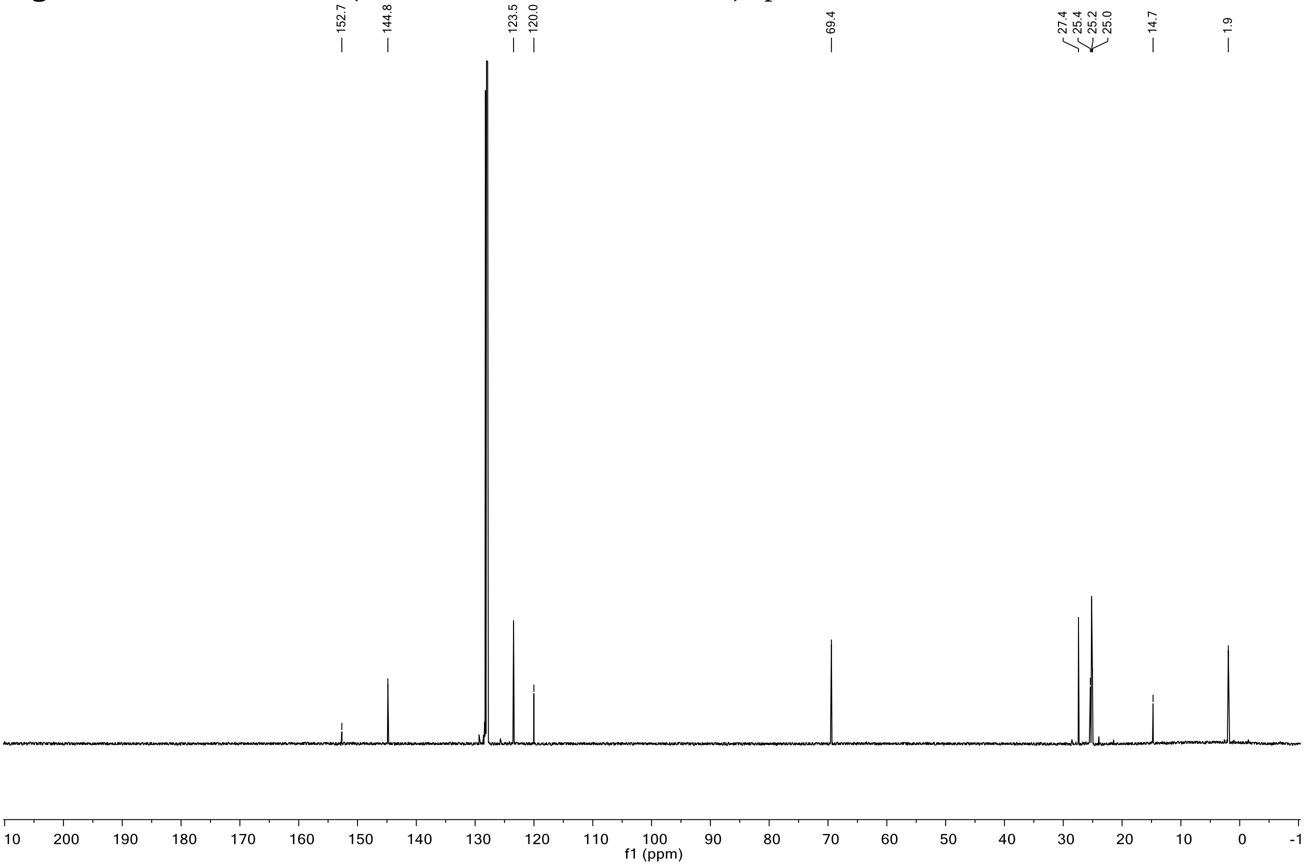

Reaction of [{SiN^{Dipp}}MgNa]$_2$ (6) towards various bulky bases: Isolation of [{SiN^{Dipp}}$_2$Mg$_2$] (8).

In J Young's NMR tubes, [{SiN^{Dipp}}MgNa]$_2$ (**6**, 21.6 mg, 0.02 mmol) was dissolved in 0.4 mL of d^6-benzene. The following bulky bases were then added to each tube of the bright yellow solution.

a. Quinuclidine (4.4 mg, 0.04 mmol)
b. 1,3-diisopropyl-4,5-dimethyl-2-ylidene (7.2 mg, 0.04 mmol)
c. 1,3-bis(2,6-diisopropylphenyl)-2-ylidene (15.5 mg, 0.04 mmol)
d. [{(SiN^{Dipp})Al}K]$_2$ (22.4 mg, 0.02 mmol)

All reaction mixtures were kept at 40 ^{o}C and monitored by ^{1}H NMR spectroscopy over a long reaction period (*c.a.*1 month). Whilst no significant change was observed in the ^{1}H NMR spectra, in each case a grey powder and colourless crystals were observed to form from the reaction mixture. The identity of the colourless single crystals was then confirmed to be **8** by X-ray diffraction analysis.

Synthesis of [{SiN^{Dipp}}$_2$Mg$_2$-(NPh$_2$)$_2$]Na$_2$ (10)

In a J Young's tube, [{SiN^{Dipp}}MgNa]$_2$ (**6**, 21.6 mg, 0.02 mmol) was dissolved in 0.4 mL of d$_6$-benzene before the addition of NaNPh$_2$ (7.6 mg, 0.04 mmol) to the bright yellow solution. No significant change was observed by ^{1}H NMR spectroscopy after the reaction mixture was kept at room temperature for 12 hours, the reaction mixture was then kept at 40 ^{o}C for 3 days before the complete conversion of the starting material was observed. At this point, the reaction mixture was colourless and a grey powder was observed to have deposited. The reaction mixture was then filtered, and kept under vacuum to remove all volatiles, washed with hexane (0.5ml x 2) affording **10** as a pale-yellow powder. Yield 24.3 mg, 64 %, Anal. Calc'd for $C_{120}H_{156}Mg_2N_6Na_2Si_4$ (**10**, 1889.46): C, 71.01; H, 9.89. Found: C, 71.78; H, 7.91 %. A colourless single crystal suitable for X-ray crystallography was obtained by gradually cooling of a saturated solution of **10** in benzene from 60 ^{o}C. ^{1}H NMR (500 MHz, 298 K, Benzene-*d*$_6$) δ 7.12 – 6.93 (m, 4H, *m*-C$_6$*H*$_3$), 6.86 – 6.76 (m, 6H, Ar*H* on NPh$_2$), 6.54 – 6.50 (m, 2H, *p*-C$_6$*H*$_3$), 6.44 – 6.33 (m, 4H, Ar*H* on NPh$_2$), 4.17 (sept, J = 6.8 Hz, 4H, CHMe$_2$), 1.38 (s, 4H, SiC*H*$_2$), 1.35 (d, J = 6.8 Hz, 12H, CH*Me*$_2$), 1.03 (d, J = 6.8 Hz, 12H, CH*Me*$_2$), 0.41 (s, 12H, Si*Me*$_2$). ^{13}C NMR (126 MHz, 298 K, Benzene-*d*$_6$) δ 153.7 (*i*-Ar*C* on NPh$_2$), 146.2 (*i*-*C*$_6$H$_3$), 144.4 (*o*-*C*$_6$H$_3$), 130.4 (Ar*C* on NPh$_2$), 123.4 (*m*-*C*$_6$H$_3$), 122.7 (Ar*C* on NPh$_2$), 118.7 (Ar*C* on NPh$_2$), 117.5 (*p*-*C*$_6$H$_3$), 27.9 (*C*HMe$_2$), 25.9 (CH*Me*$_2$), 24.8 (CH*Me*$_2$), 15.9 (Si*C*H$_2$), 2.1 (Si*Me*$_2$). *Minor amount free {SiN^{Dipp}}H$_2$ was also observed by both ^{1}H and ^{13}C NMR spectroscopy in the crude product.

Figure S3. ^{1}H NMR (500 MHz, 298 K, d$_6$-benzene) spectrum of **10**. *free {SiN^{Dipp}}H$_2$.

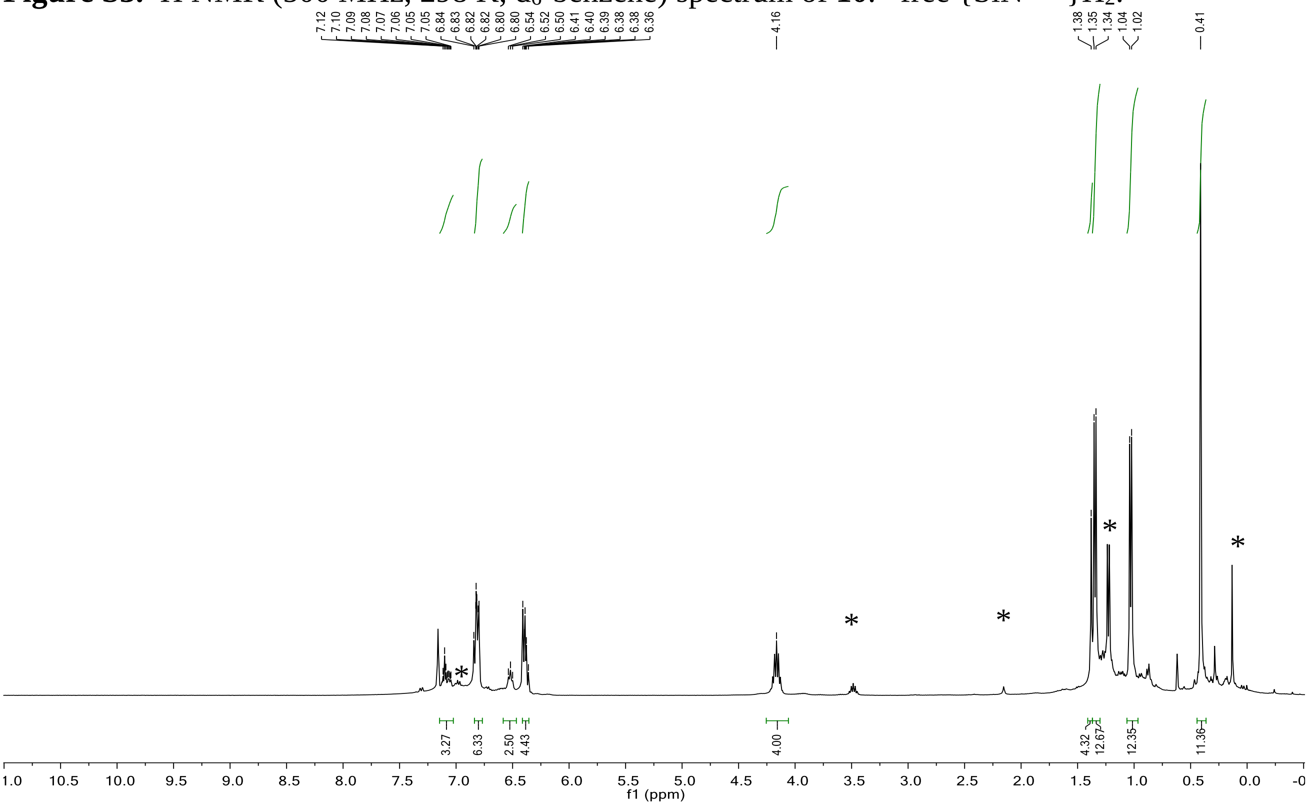

Figure S4. ^{13}C{^{1}H} NMR (126 MHz, 298 K, d$_6$-benzene) spectrum of **10**. *free {SiN^{Dipp}}H$_2$.

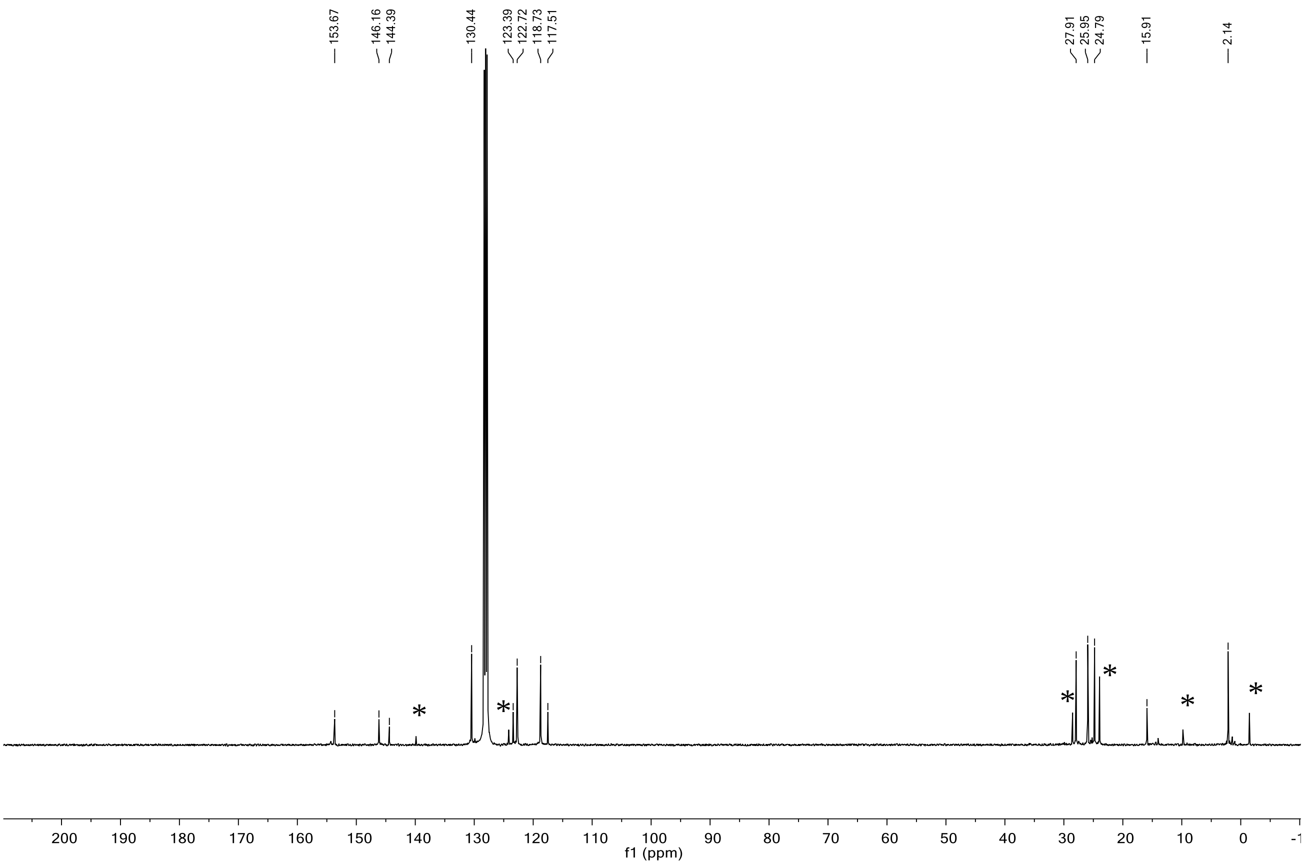

Figure S5. ¹H-¹³C HSQC spectrum of **10**.

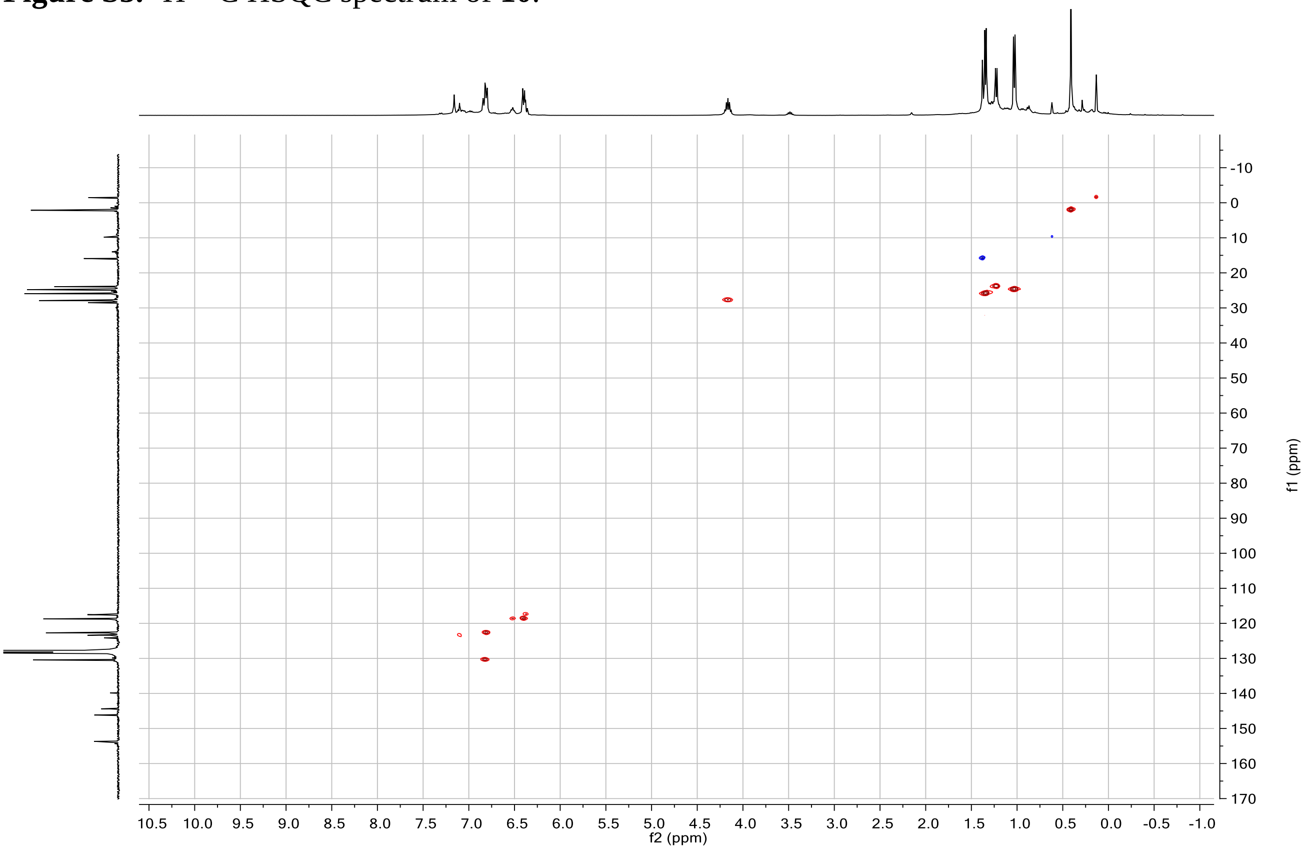

Figure S6. ¹H-¹³C HMBC spectrum of **10**.

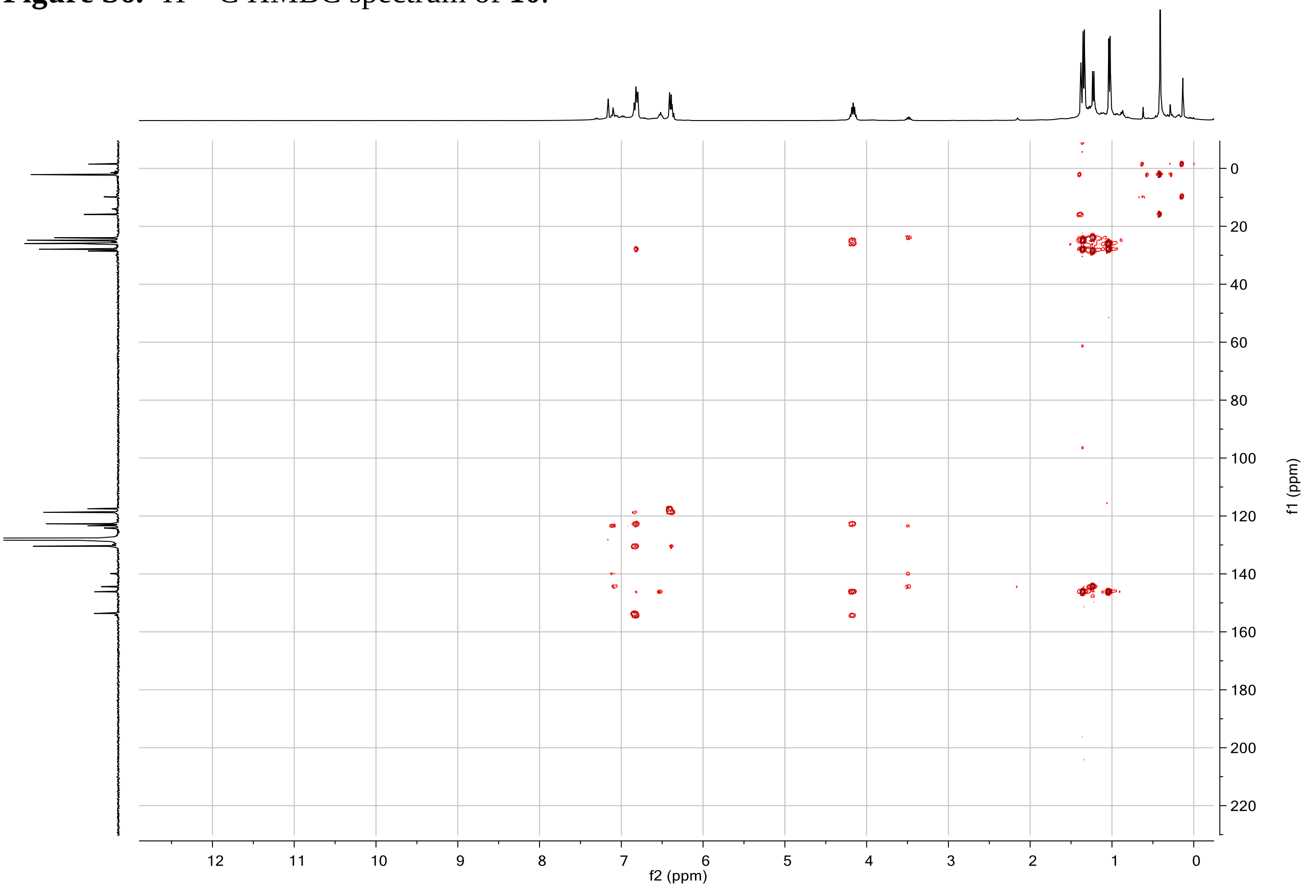

1.3. UV/Vis. spectroscopy of [{SiN^{Dipp}}MgNa]$_2$ (6) in benzene

The sample suitable for analysis by UV/Vis spectroscopy was prepared by dissolving 1.0 mg of {SiN^{Dipp}}MgNa]$_2$ (**6**) in a minimal amount of benzene, before diluting the solution to precisely 10mL. The concentration of the sample used for optical spectroscopy was 8.5 x 10^{-8} M.

Figure S7. Plotted UV/Vis spectrum of 8.5 x 10^{-8} M solution of [{SiN^{Dipp}}MgNa]$_2$ (**6**) in benzene. (Wavelength: 250 – 500 nm)

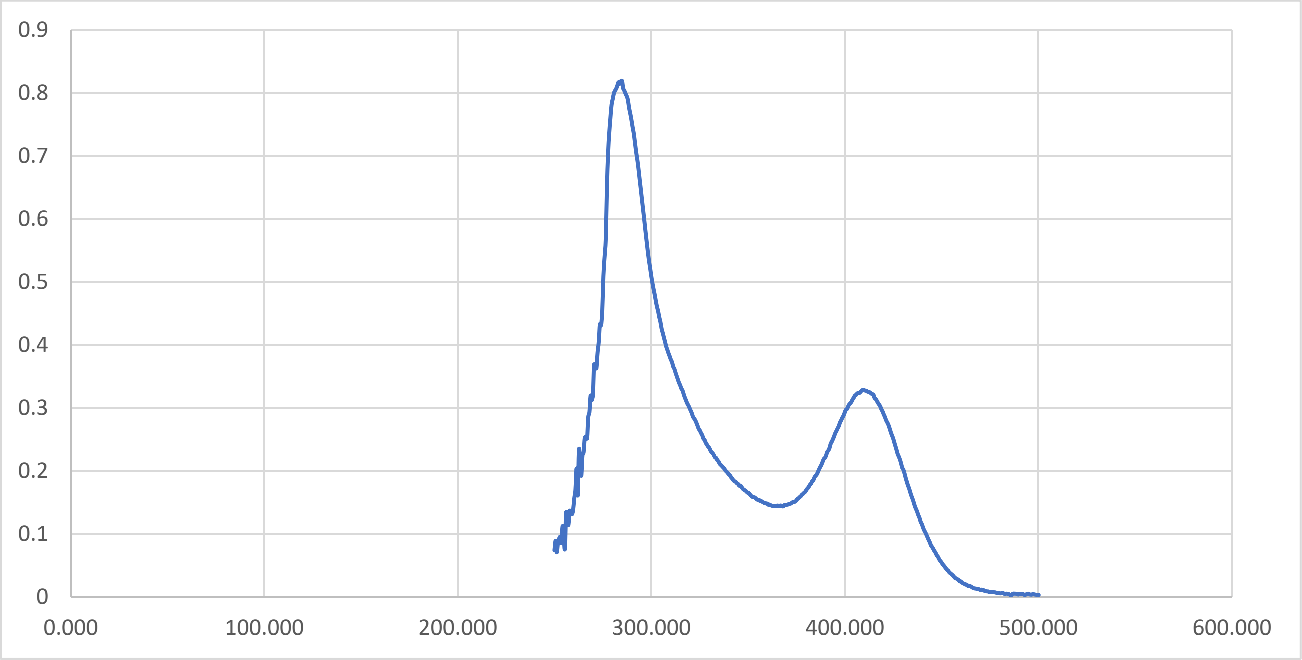

Table S1. Raw data points of UV/Vis Measurement of the 8.5 x 10^{-8} M solution of [{SiN^{Dipp}}MgNa]$_2$ (**6**) in benzene.

**Wavelength (nm)**	**Absorbance**	**Wavelength (nm)**	**Absorbance**	**Wavelength (nm)**	**Absorbance**	**Wavelength (nm)**	**Absorbance**
249.944	0.073787638	266.820	0.251443516	283.677	0.814547359	300.513	0.499559022
250.547	0.08886872	267.423	0.285378824	284.278	0.818629506	301.114	0.489594953
251.150	0.070666792	268.025	0.29313782	284.880	0.819149373	301.714	0.480476189
251.753	0.08544637	268.627	0.319272637	285.482	0.807263569	302.315	0.470354224
252.356	0.092357012	269.230	0.312182958	286.083	0.803487257	302.916	0.460786447
252.959	0.09522093	269.832	0.320441061	286.685	0.798879813	303.517	0.453726222
253.562	0.085636078	270.434	0.368798313	287.286	0.795057621	304.118	0.443978089
254.165	0.112356323	271.036	0.363734987	287.888	0.789073191	304.718	0.436455507
254.768	0.093258431	271.638	0.362778519	288.489	0.776872011	305.319	0.425855549
255.370	0.076606685	272.241	0.388134429	289.091	0.767832032	305.920	0.418973124
255.973	0.13365149	272.843	0.404516846	289.692	0.758147383	306.520	0.411782092
256.576	0.116430679	273.445	0.433000755	290.293	0.746640705	307.121	0.404730712
257.179	0.114250107	274.047	0.431050349	290.895	0.736627078	307.722	0.397364032
257.782	0.136716153	274.649	0.452584058	291.496	0.722778839	308.322	0.391954927
258.384	0.135561368	275.251	0.509192666	292.097	0.707797508	308.923	0.386479505
258.987	0.131054262	275.853	0.539415429	292.699	0.695576912	309.523	0.381758521
259.590	0.137618269	276.455	0.566570516	293.300	0.681708121	310.124	0.376405196
260.193	0.156326141	277.057	0.647194	293.901	0.665039843	310.724	0.372648853
260.795	0.169420018	277.659	0.703404586	294.502	0.649333277	311.325	0.365488249
261.398	0.20374036	278.261	0.735911289	295.103	0.632678735	311.925	0.361958571
262.000	0.161136087	278.863	0.760066916	295.705	0.616261521	312.526	0.355928363
262.603	0.233492843	279.464	0.781185544	296.306	0.600280131	313.126	0.350608157
263.206	0.21893875	280.066	0.790253628	296.907	0.582959996	313.726	0.345196206
263.808	0.192198768	280.668	0.799407305	297.508	0.566761133	314.327	0.339906382
264.411	0.22452088	281.270	0.803751201	298.109	0.5506151	314.927	0.335924673
265.013	0.229368128	281.872	0.806809733	298.710	0.535649681	315.527	0.330679127
265.615	0.252283975	282.473	0.811828104	299.311	0.523923233	316.127	0.327749852
266.218	0.254317844	283.075	0.817015229	299.912	0.510869147	316.728	0.321841178

Table S1. (continued) Raw data points of UV/Vis Measurement of the 8.5 x 10^{-8} M solution of [{SiN^{Dipp}}MgNa]$_2$ (**6**) in benzene.

Wavelength (nm)	Absorbance	Wavelength (nm)	Absorbance	Wavelength (nm)	Absorbance	Wavelength (nm)	Absorbance
317.328	0.316446362	334.122	0.217006492	350.894	0.162891307	367.644	0.143756247
317.928	0.31197642	334.721	0.215043836	351.493	0.160767971	368.242	0.143829406
318.528	0.307363436	335.320	0.211424216	352.091	0.158981405	368.840	0.145858304
319.128	0.304069845	335.920	0.209407676	352.690	0.158264442	369.438	0.145550355
319.728	0.300141574	336.519	0.207565549	353.288	0.158008851	370.035	0.14626041
320.328	0.295762885	337.118	0.205472311	353.887	0.156762533	370.633	0.146535089
320.928	0.291696132	337.718	0.203553584	354.485	0.154879565	371.231	0.147746978
321.528	0.286017763	338.317	0.200513332	355.084	0.154061543	371.829	0.147813309
322.128	0.283299865	338.916	0.198823813	355.682	0.153967037	372.426	0.148880528
322.728	0.280315882	339.515	0.196548971	356.280	0.152011881	373.024	0.150218462
323.328	0.27616221	340.114	0.194313893	356.879	0.151956066	373.621	0.150774669
323.928	0.271355988	340.713	0.192566136	357.477	0.150636892	374.219	0.151015175
324.528	0.266496491	341.313	0.189478724	358.075	0.149702955	374.816	0.152637809
325.128	0.263967769	341.912	0.187405226	358.674	0.148656074	375.414	0.155215957
325.727	0.259759472	342.511	0.184731754	359.272	0.148459177	376.011	0.156291144
326.327	0.256773386	343.110	0.18344344	359.870	0.148004968	376.609	0.158266408
326.927	0.251218733	343.709	0.182320381	360.468	0.14623983	377.206	0.159531003
327.527	0.249445949	344.307	0.180183611	361.067	0.146598092	377.803	0.162041681
328.126	0.244519663	344.906	0.179081024	361.665	0.145539098	378.401	0.163428634
328.726	0.241831863	345.505	0.176286657	362.263	0.144787743	378.998	0.165393482
329.326	0.238647076	346.104	0.176557215	362.861	0.144071165	379.595	0.16693087
329.925	0.236661316	346.703	0.174183149	363.459	0.143942888	380.193	0.170131473
330.525	0.232549061	347.302	0.171482596	364.057	0.144118044	380.790	0.172290662
331.124	0.229679999	347.901	0.17002182	364.655	0.144304125	381.387	0.17475844
331.724	0.228112746	348.499	0.168983721	365.253	0.145035034	381.984	0.177987752
332.323	0.225051717	349.098	0.167362646	365.851	0.144062219	382.581	0.179928737
332.923	0.221474299	349.697	0.165531298	366.449	0.144706804	383.178	0.18389742
333.522	0.220530545	350.295	0.165026462	367.047	0.14498961	383.775	0.185344405

Table S1. (continued) Raw data points of UV/Vis Measurement of the 8.5 x 10^{-8} M solution of [{SiN^{Dipp}}MgNa]$_2$ (**6**) in benzene.

**Wavelength (nm)**	**Absorbance**	**Wavelength (nm)**	**Absorbance**	**Wavelength (nm)**	**Absorbance**	**Wavelength (nm)**	**Absorbance**
384.372	0.190036036	401.674	0.30203978	414.187	0.321719007	431.446	0.189001451
384.969	0.19232492	402.270	0.305543469	414.783	0.321009722	432.041	0.182751364
385.566	0.194671208	402.866	0.307357234	415.378	0.315737813	432.635	0.176908674
386.163	0.198752058	403.462	0.309857816	415.974	0.314162816	433.230	0.172120364
386.760	0.202835305	404.058	0.313578846	416.569	0.311137661	433.824	0.166036872
387.357	0.206539402	404.654	0.316246285	417.165	0.307829559	434.419	0.160365349
387.954	0.209921874	405.251	0.319599883	417.760	0.30514952	435.013	0.155662718
388.551	0.214587733	405.847	0.320744704	418.355	0.302051761	435.608	0.150096614
389.148	0.21897166	406.442	0.323030561	418.951	0.297488343	436.202	0.144036533
389.744	0.220593598	407.038	0.323488466	419.546	0.294252344	436.797	0.139542074
390.341	0.224527425	407.634	0.323800041	420.141	0.28953284	437.391	0.135178904
390.938	0.230120916	408.230	0.325815293	420.737	0.285570963	437.986	0.129778418
391.535	0.232458372	408.826	0.327577021	421.332	0.28048671	438.580	0.12562695
392.131	0.23673822	409.422	0.328755505	421.927	0.27697508	439.174	0.119252423
392.728	0.243267884	410.018	0.328092765	422.522	0.273196803	439.768	0.115813823
393.325	0.24661827	410.613	0.327418621	423.117	0.267916173	440.363	0.110708118
393.921	0.250766302	411.209	0.327141446	423.712	0.262040949	440.957	0.106037579
394.518	0.255225501	411.805	0.325683439	424.307	0.256804293	441.551	0.102741313
395.114	0.260252217	412.400	0.325399993	424.902	0.252407467	442.145	0.098756296
395.711	0.263648044	412.996	0.324504864	425.497	0.246045361	442.739	0.094534179
396.307	0.267673554	413.592	0.32297687	426.092	0.23990597	443.334	0.090399388
396.904	0.27117287	414.187	0.321719007	426.687	0.233795033	443.928	0.086674814
397.500	0.276690016	414.783	0.321009722	427.282	0.227111919	444.522	0.081729989
398.096	0.280092072	415.378	0.315737813	427.877	0.222891537	445.116	0.079186436
398.693	0.284208572	415.974	0.314162816	428.472	0.218140874	445.710	0.076129241
399.289	0.287425802	416.569	0.311137661	429.067	0.212178852	446.304	0.072662563
399.885	0.292003691	417.165	0.307829559	429.662	0.205042376	446.897	0.070122563
400.482	0.296717113	417.760	0.30514952	430.256	0.201803924	447.491	0.066117356
401.078	0.29802057	418.355	0.302051761	430.851	0.196140145	448.085	0.063980092

Table S1. (continued) Raw data points of UV/Vis Measurement of the 8.5 x 10^{-8} M solution of [{SiN^{Dipp}}MgNa]$_2$ (**6**) in benzene.

**Wavelength (nm)**	**Absorbance**	**Wavelength (nm)**	**Absorbance**	**Wavelength (nm)**	**Absorbance**	**Wavelength (nm)**	**Absorbance**
448.679	0.060306395	465.886	0.014929147	483.067	0.004924648	500.222	0.002993524
449.273	0.057325862	466.479	0.013946133	483.659	0.0050769	500.813	0.002372078
449.867	0.055209082	467.072	0.013383425	484.251	0.004726146	501.404	0.00342044
450.460	0.052287217	467.665	0.013132219	484.843	0.004212296	501.995	0.003078948
451.054	0.050031025	468.258	0.012693599	485.435	0.00306395	502.586	0.00352845
451.648	0.0477348	468.850	0.012356727	486.027	0.002555704	503.176	0.002782341
452.241	0.045210602	469.443	0.011980006	486.619	0.004469495	503.767	0.002717492
452.835	0.042890308	470.036	0.010968993	487.210	0.005210972	504.358	0.003397004
453.428	0.041597742	470.628	0.011458629	487.802	0.005036081	504.949	0.002790536
454.022	0.038712204	471.221	0.010448112	488.394	0.004991378	505.540	0.002037015
454.615	0.037398354	471.814	0.010618603	488.986	0.004635443	506.131	0.002756877
455.209	0.035737151	472.406	0.009068805	489.577	0.003693696	506.721	0.002861545
455.802	0.034221179	472.999	0.008948253	490.169	0.004539572	507.312	0.003647812
456.396	0.032096944	473.591	0.008659539	490.760	0.00436974	507.903	0.003994249
456.989	0.029965763	474.184	0.008543136	491.352	0.004390501	508.493	0.002551035
457.583	0.029329174	474.776	0.007386553	491.944	0.004716864	509.084	0.002807066
458.176	0.028033429	475.369	0.007886524	492.535	0.003544121	509.674	0.002958955
458.769	0.026917751	475.961	0.007375393	493.127	0.003077621	510.265	0.002899423
459.362	0.024640397	476.553	0.007769226	493.718	0.003880216	510.855	0.002748393
459.956	0.024527665	477.146	0.007273436	494.309	0.004722284	511.446	0.003298286
460.549	0.022742687	477.738	0.007113992	494.901	0.005011722	512.036	0.002607302
461.142	0.021477466	478.330	0.006497289	495.492	0.003975498	512.627	0.002527457
461.735	0.02073447	478.923	0.006426216	496.083	0.003536001	513.217	0.002752202
462.328	0.019557453	479.515	0.005978354	496.675	0.003601089	513.807	0.001822424
462.921	0.019469962	480.107	0.005636071	497.266	0.004601075	514.398	0.002489358
463.514	0.017706921	480.699	0.005602824	497.857	0.003852352	514.988	0.002876177
464.107	0.01697003	481.291	0.005955484	498.448	0.003607674	515.578	0.002787975
464.700	0.01698633	481.883	0.005848467	499.039	0.003192447	516.168	0.002233869
465.293	0.0161109	482.475	0.004731993	499.630	0.002826344	516.759	0.002467749

Table S1. (continued) Raw data points of UV/Vis Measurement of the 8.5 x 10^{-8} M solution of [{SiN^{Dipp}}MgNa]$_2$ (**6**) in benzene.

Wavelength (nm)	Absorbance	Wavelength (nm)	Absorbance	Wavelength (nm)	Absorbance	Wavelength (nm)	Absorbance
522.659	0.001892246	539.750	0.001867205	556.813	0.001426911	573.847	0.000819932
523.248	0.002833237	540.338	0.001672503	557.401	0.000856681	574.434	0.001143914
523.838	0.001794385	540.927	0.001996748	557.988	0.001339198	575.021	0.00073973
524.428	0.002716215	541.516	0.001457194	558.576	0.001011824	575.608	0.001082704
525.018	0.002684908	542.105	0.001354657	559.164	0.001733945	576.195	0.001432491
525.607	0.003320955	542.694	0.001943187	559.752	0.001314849	576.781	0.001370626
526.197	0.002449011	543.282	0.001698422	560.339	0.001320543	577.368	0.001008925
526.787	0.003090944	543.871	0.001502305	560.927	0.001113142	577.955	0.000716084
527.376	0.00279624	544.460	0.002416294	561.515	0.000799784	578.541	0.001464685
527.966	0.002303785	545.048	0.001396332	562.102	0.001303966	579.128	0.001234736
528.555	0.001533644	545.637	0.001786951	562.690	0.00168392	579.715	0.001453065
529.145	0.002166706	546.225	0.001747564	563.277	0.00228379	580.301	0.001840673
529.734	0.002113355	546.814	0.001065921	563.865	0.002040302	580.888	0.001687654
530.324	0.002643335	547.402	0.001215884	564.452	0.001643442	581.474	0.001125238
530.913	0.00226848	547.990	0.001684311	565.040	0.000710435	582.061	0.001243371
531.502	0.002068665	548.579	0.001004439	565.627	0.001541812	582.647	0.001293061
532.092	0.001291893	549.167	0.001683747	566.215	0.001413078	583.233	0.001201647
532.681	0.002365512	549.756	0.00156474	566.802	0.001293422	583.820	0.001248793
533.270	0.001519831	550.344	0.001522366	567.389	0.001121732	584.406	0.000628471
533.859	0.001185428	550.932	0.001036891	567.977	0.001215921	584.992	0.00100374
534.448	0.001937768	551.520	0.001774024	568.564	0.000915978	585.578	0.0007429
535.038	0.001714513	552.108	0.001630284	569.151	0.001089017	586.165	0.00130089
535.627	0.001520016	552.697	0.001836549	569.738	0.001836942	586.751	0.000931314
536.216	0.00201205	553.285	0.000957608	570.325	0.001009686	587.337	0.00135047
536.805	0.001986878	553.873	0.001439387	570.912	0.000812565	587.923	0.000491102
537.394	0.002131207	554.461	0.001046078	571.499	0.000647579	588.509	0.000988514
537.983	0.002000772	555.049	0.001761732	572.086	0.001978817	589.095	0.000993745
538.572	0.002307014	555.637	0.00140754	572.673	0.001302475	589.681	0.001272648
539.161	0.001394176	556.225	0.001702074	573.260	0.001245525	590.267	0.000521241

Table S1. (continued) Raw data points of UV/Vis Measurement of the 8.5 x 10^{-8} M solution of [{SiN^{Dipp}}$MgNa]_2$ (**6**) in benzene.

**Wavelength (nm)**	**Absorbance**	**Wavelength (nm)**	**Absorbance**	**Wavelength (nm)**	**Absorbance**	**Wavelength (nm)**	**Absorbance**
590.853	0.000426462	607.829	0.000803214	624.776	0.000523175	641.693	0.000589309
591.439	0.000629709	608.414	0.000339881	625.360	0.001070539	642.276	-0.000132219
592.025	-7.29368E-05	608.999	0.000377664	625.944	0.0006818	642.858	0.000254524
592.611	0.000705168	609.584	0.000564779	626.528	0.0007648	643.441	0.000395692
593.196	0.000176821	610.169	0.000498658	627.111	0.0011996	644.024	-0.000182937
593.782	0.001118073	610.753	0.001055641	627.695	0.000362719	644.606	0.000491141
594.368	0.001459506	611.338	0.000394023	628.279	0.000936951	645.189	0.000294774
594.953	0.000932735	611.923	0.000973524	628.862	0.000977172	645.772	3.53239E-05
595.539	0.001122059	612.507	0.000824087	629.446	0.001744721	646.354	0.000656877
596.125	0.001047756	613.092	0.000966076	630.029	0.001385229	646.937	0.000121313
596.710	0.001245649	613.677	0.000939586	630.613	0.000582646	647.519	5.06085E-05
597.296	0.000320368	614.261	0.000651105	631.196	0.000777784	648.102	0.000604356
597.881	0.000335842	614.846	0.001575599	631.780	0.000855679	648.684	1.40509E-05
598.467	0.000199864	615.430	0.001117858	632.363	0.00065619	649.266	-1.42745E-05
599.052	0.000117599	616.014	0.000559544	632.947	0.001067722	649.849	2.74233E-05
599.638	0.00065126	616.599	0.000622501	633.530	0.000756435	650.431	-0.000357539
600.223	0.000602411	617.183	0.000420078	634.113	0.001191292	651.013	0.000394097
600.808	0.000928294	617.767	0.000810602	634.697	0.000887903	651.595	0.00044821
601.394	0.000696354	618.352	-7.50293E-06	635.280	0.001007847	652.178	-4.52317E-05
601.979	0.001314907	618.936	3.7212E-05	635.863	0.00073059	652.760	0.000736036
602.564	0.001311396	619.520	0.000216828	636.446	0.000602595	653.342	0.000302246
603.149	0.001356682	620.104	0.0002285	637.029	0.000915322	653.924	-0.000227551
603.734	0.001159918	620.688	0.000619887	637.612	0.000219601	654.506	0.000851466
604.319	0.000962052	621.272	0.000498032	638.195	-0.000116242	655.088	0.000101571
604.905	0.001648805	621.856	0.00068885	638.778	0.000565031	655.670	-0.000177545
605.490	0.000835227	622.440	0.000935536	639.361	-0.000120525	656.252	0.000424012
606.075	0.000775397	623.024	-0.000462213	639.944	0.000586446	656.834	0.000496787
606.660	0.001069318	623.608	0.000782765	640.527	0.000254339	657.415	8.97966E-05
607.245	0.000678485	624.192	0.000248502	641.110	-0.000406146	657.997	0.000614899

Table S1. (continued) Raw data points of UV/Vis Measurement of the 8.5 x 10^{-8} M solution of [{SiN^{Dipp}}MgNa]$_2$ (**6**) in benzene.

Wavelength (nm)	Absorbance	Wavelength (nm)	Absorbance	Wavelength (nm)	Absorbance	Wavelength (nm)	Absorbance
658.579	0.000333404	675.434	-0.000671554	692.258	0.001046501	709.050	0.00092727
659.161	0.00046668	676.015	0.000455636	692.838	0.000650631	709.629	0.000376526
659.742	0.000178458	676.596	0.000888923	693.417	-0.000522049	710.207	0.000186789
660.324	-0.000579955	677.176	3.88567E-05	693.997	0.000463341	710.786	0.001119114
660.906	0.000626878	677.757	0.000653242	694.576	0.00052373	711.364	0.000958538
661.487	-0.000222972	678.337	0.001366048	695.156	-0.000300196	711.942	-0.000476762
662.069	-0.00063869	678.918	-0.000616615	695.735	0.000558926	712.521	-0.00100959
662.650	0.000848859	679.498	7.80663E-05	696.314	0.000617002	713.099	0.000414566
663.232	0.00058278	680.079	0.000805019	696.894	-0.000154835	713.677	-0.001332326
663.813	0.000179209	680.659	-0.000156438	697.473	0.000469657	714.255	-0.001422834
664.395	0.000873374	681.239	0.000435427	698.052	0.001023273	714.833	0.000153664
664.976	-2.28098E-05	681.819	0.00077758	698.631	-0.00123362	715.411	-0.000256186
665.557	-0.000251895	682.400	-0.000427626	699.211	-0.000824173	715.990	-0.001036586
666.139	0.000461104	682.980	-0.000137649	699.790	0.00043359	716.568	-7.50398E-05
666.720	-0.000662179	683.560	0.000616853	700.369	-0.000590011	717.146	-0.000373501
667.301	8.43365E-05	684.140	-0.000678253	700.948	-0.000727967	717.723	-0.001670132
667.882	0.000667597	684.720	0.000260938	701.527	0.000996273	718.301	-0.00086036
668.463	-0.000251497	685.300	0.000904244	702.106	-0.000650771	718.879	0.000631967
669.045	0.000570491	685.880	-0.000246389	702.685	-0.001367957	719.457	-0.000631622
669.626	0.000838655	686.460	4.71803E-05	703.264	0.000469082	720.035	-0.000652756
670.207	-0.000539087	687.040	0.001392932	703.842	0.000344023	720.613	0.00020056
670.788	0.000156198	687.620	-0.000167757	704.421	-0.001085781	721.190	-0.000223219
671.369	0.000212654	688.200	-0.000347692	705.000	0.0006323	721.768	-0.0013332
671.950	-0.001093896	688.780	0.000854417	705.579	0.000396418	722.346	-0.000326036
672.530	0.000123407	689.360	-0.000564262	706.157	-0.000942492	722.923	0.000343326
673.111	0.000823723	689.940	-0.000545769	706.736	-0.000475753	723.501	-0.001490972
673.692	-0.000407116	690.519	-5.14301E-05	707.315	9.67889E-05	724.078	-0.001717674
674.273	-1.30887E-05	691.099	-0.000141972	707.893	-0.000620274	724.656	0.000439459
674.854	0.000252282	691.679	-0.000485854	708.472	-0.001267335	725.233	0.000103551

Table S1. (continued) Raw data points of UV/Vis Measurement of the 8.5 x 10^{-8} M solution of [{SiN^{Dipp}}MgNa]$_2$ (**6**) in benzene.

**Wavelength (nm)**	**Absorbance**	**Wavelength (nm)**	**Absorbance**	**Wavelength (nm)**	**Absorbance**	**Wavelength (nm)**	**Absorbance**
725.810	-0.000867511	742.538	-0.000942187	759.233	-0.001500683	775.894	0.000207515
726.388	0.00017692	743.114	0.000282911	759.808	0.000381297	776.468	0.000341212
726.965	0.000907678	743.690	-0.000424953	760.383	0.001425622	777.041	-0.001462433
727.542	-0.000442865	744.267	-0.001454055	760.958	0.000335094	777.615	-0.001047879
728.120	-0.000296067	744.843	-0.000251466	761.533	-0.001075654	778.189	0.001191863
728.697	0.001389609	745.419	0.000216211	762.108	-0.000203513	778.763	-9.72571E-05
729.274	0.000373196	745.995	-0.00126126	762.682	0.001369048	779.337	-0.002033097
729.851	-0.000396586	746.571	-0.001772951	763.257	-0.001033833	779.910	-0.001411329
730.428	3.91603E-05	747.147	0.000486302	763.832	-0.001597926	780.484	0.001225878
731.005	0.000169111	747.723	0.000435503	764.407	0.00092836	781.058	0.000138656
731.582	-0.001892588	748.298	-0.001049096	764.982	0.001243184	781.631	-0.001642116
732.159	-0.001666164	748.874	-0.000447227	765.556	-5.49851E-05	782.205	-0.001169607
732.736	-9.62574E-05	749.450	0.000996398	766.131	-0.000778265	782.778	0.000162965
733.313	-0.000934284	750.026	0.000366204	766.705	0.001250696	783.352	0.000548148
733.890	-0.002154943	750.602	-0.000919181	767.280	0.001112533	783.925	-0.000536035
734.467	-0.000890895	751.177	-2.49765E-06	767.855	-0.001631775	784.498	0.00027242
735.043	-0.000274575	751.753	0.000386845	768.429	-0.000985217	785.072	0.001746316
735.620	-9.87128E-05	752.328	-0.000291206	769.003	0.000865768	785.645	0.000609526
736.197	-0.001057561	752.904	-0.000352024	769.578	0.001397873	786.218	-0.001547531
736.774	0.000538995	753.480	0.001270295	770.152	-0.001374008	786.791	-0.001466022
737.350	0.000193286	754.055	0.001494891	770.727	-0.001374117	787.364	0.000659628
737.927	-0.001006666	754.630	-0.000571106	771.301	0.001154907	787.938	0.000848527
738.503	-0.000220347	755.206	-0.000656737	771.875	0.000992524	788.511	-0.001252185
739.080	3.15674E-05	755.781	0.000530783	772.449	-0.001260012	789.084	-0.002054994
739.656	-0.000391937	756.357	0.00034825	773.023	-0.002052696	789.657	0.000220186
740.233	-0.001073719	756.932	-0.001125774	773.598	-0.000347948	790.230	0.000495925
740.809	-0.00016477	757.507	-0.000376685	774.172	-9.01638E-05	790.803	-0.001283406
741.385	-0.000195888	758.082	0.00056566	774.746	-0.001402882	791.375	-0.001245852
741.962	-0.001426473	758.657	-0.000477812	775.320	-0.00146216	791.948	0.000209846

Table S1. (continued) Raw data points of UV/Vis Measurement of the 8.5 x 10^{-8} M solution of [{SiN^{Dipp}}MgNa]$_2$ (**6**) in benzene.

Wavelength (nm)	Absorbance	Wavelength (nm)	Absorbance	Wavelength (nm)	Absorbance	Wavelength (nm)	Absorbance
792.521	0.0003517	809.114	0.000887049	825.673	-0.000987503	842.196	-0.002896371
793.094	-0.00126951	809.686	0.001604262	826.243	-0.00122933	842.766	-0.000635958
793.667	-0.002800512	810.257	-0.000352019	826.814	0.000541615	843.335	-3.70827E-05
794.239	-0.000980744	810.829	-0.002283885	827.384	0.001128642	843.904	-0.001598595
794.812	0.001885366	811.400	2.03378E-05	827.954	-0.000927944	844.473	-0.00216515
795.384	-0.000155728	811.972	0.001279428	828.524	-0.002947843	845.042	-0.001000694
795.957	-0.001506794	812.543	-0.000521267	829.094	-0.00163937	845.611	0.000167251
796.529	-0.000443051	813.114	-0.002030931	829.665	0.001151137	846.180	-0.000645733
797.102	0.001020398	813.686	-0.000224681	830.235	-0.000450017	846.748	-0.002528105
797.674	-0.000491539	814.257	0.001202913	830.805	-0.002221739	847.317	-0.003342713
798.247	-0.002238659	814.828	0.000247319	831.375	-0.000744508	847.886	-0.00073547
798.819	-0.001867149	815.399	-0.000463455	831.945	0.000520212	848.455	0.000955266
799.391	0.000167751	815.970	-0.001494826	832.514	0.000543935	849.023	-3.67531E-05
799.964	-0.000730706	816.541	0.001304187	833.084	-0.000826602	849.592	-0.001863436
800.536	-0.001971375	817.112	0.00145189	833.654	-0.001871426	850.161	-0.002356531
801.108	-0.002929711	817.683	-0.001112552	834.224	-0.001380595	850.729	1.44606E-05
801.680	-0.000252902	818.254	-0.002441384	834.794	0.000597779	851.298	0.000634464
802.252	0.000588815	818.825	-0.001675969	835.363	0.00029851	851.866	0.000480456
802.824	-0.000852086	819.396	0.00061796	835.933	-0.002440694	852.435	-0.001466759
803.396	-0.001634899	819.967	-0.000591422	836.503	-0.003046418	853.003	-0.001764566
803.968	-0.000491468	820.538	-0.002655016	837.072	-0.001103689	853.571	-0.000777112
804.540	0.000947773	821.108	-0.003246191	837.642	0.001117638	854.140	7.11119E-05
805.112	-0.000788001	821.679	0.000336243	838.211	0.000448829	854.708	-0.000630841
805.684	-0.002412567	822.250	0.001702288	838.781	-0.001870829	855.276	-0.002472123
806.256	-0.000665428	822.820	-0.000463473	839.350	-0.001689953	855.844	-0.00207984
806.828	0.001708385	823.391	-0.002224482	839.919	-0.000699638	856.412	0.000512565
807.399	0.000508078	823.962	-0.000397895	840.489	0.001157975	856.981	0.00066578
807.971	-0.001952768	824.532	0.000771577	841.058	-0.000560677	857.549	7.14478E-05
808.543	-0.000733004	825.102	-0.000385059	841.627	-0.002445975	858.117	-0.002790192

Table S1. (continued) Raw data points of UV/Vis Measurement of the 8.5 x 10^{-8} M solution of [{SiN^{Dipp}}MgNa]$_2$ (**6**) in benzene.

Wavelength (nm)	Absorbance	Wavelength (nm)	Absorbance	Wavelength (nm)	Absorbance	Wavelength (nm)	Absorbance
858.685	-0.001215575	875.137	-0.001980394	891.553	0.001160587	907.932	-7.37382E-05
859.253	0.000787197	875.704	-0.002522561	892.118	0.000807589	908.497	-0.00185747
859.820	0.001514641	876.270	-0.000728279	892.684	-0.001169712	909.061	-0.001125278
860.388	-0.001203845	876.837	0.000276732	893.249	-0.003301181	909.625	-0.000276526
860.956	-0.002604799	877.403	-0.000623909	893.814	-0.002010695	910.189	0.000789082
861.524	-0.001329655	877.970	-0.002180322	894.380	0.000473637	910.753	-0.001002516
862.091	0.001511406	878.536	-0.002253687	894.945	0.000994391	911.317	-0.002526593
862.659	-0.000222364	879.103	-0.002075778	895.510	4.18635E-05	911.881	-0.00234352
863.227	-0.001676786	879.669	0.000150652	896.075	-0.002080804	912.444	-0.00070591
863.794	-0.003032043	880.235	-0.000321021	896.640	-0.002093468	913.008	0.000151603
864.362	-0.002166391	880.802	-0.002784221	897.205	-0.000270039	913.572	-0.000102104
864.929	-0.000104628	881.368	-0.003887408	897.770	0.000371512	914.136	-0.001493787
865.497	-0.001190778	881.934	-0.001506285	898.335	-0.001318462	914.699	-0.001455894
866.064	-0.002688622	882.500	1.26347E-05	898.900	-0.002496304	915.263	-0.002375707
866.632	-0.003980725	883.067	4.03149E-05	899.465	-0.002711135	915.827	-0.000132097
867.199	-0.002927023	883.633	-0.002004208	900.030	-0.001876119	916.390	0.00115416
867.766	0.000604223	884.199	-0.003519995	900.595	0.000510889	916.954	-0.000318887
868.333	0.000805204	884.765	-0.001803169	901.159	0.001198254	917.517	-0.002555398
868.901	-0.001956647	885.331	5.77711E-05	901.724	-0.001179473	918.080	-0.003987843
869.468	-0.003848155	885.896	0.000190861	902.289	-0.002735852	918.644	-0.001422803
870.035	-0.002248919	886.462	-0.000577507	902.853	-0.002162583	919.207	-0.000702114
870.602	0.000614419	887.028	-0.002828076	903.418	0.000645191	919.770	0.000660156
871.169	0.000328372	887.594	-0.002976903	903.982	0.001099835	920.334	-0.001493027
871.736	-0.001293605	888.160	-0.000471494	904.547	0.000552368	920.897	-0.003236299
872.303	-0.002782532	888.725	0.001848315	905.111	-0.0019054	921.460	-0.003428441
872.870	-0.001848495	889.291	-1.41897E-05	905.675	-0.002632971	922.023	-0.001042243
873.437	0.000666022	889.857	-0.00216442	906.240	-0.001652935	922.586	0.001562929
874.003	0.001376157	890.422	-0.002908883	906.804	0.001646722	923.149	0.002393913
874.570	0.000187219	890.988	-0.000995271	907.368	0.002338092	923.712	-0.000928031

Table S1. (continued) Raw data points of UV/Vis Measurement of the 8.5 x 10^{-8} M solution of [{SiN^{Dipp}}MgNa]$_2$ (**6**) in benzene.

Wavelength (nm)	Absorbance	Wavelength (nm)	Absorbance	Wavelength (nm)	Absorbance	Wavelength (nm)	Absorbance
924.275	-0.001497318	940.580	-0.001213685	956.847	0.001623934	973.076	-0.001273055
924.838	-0.002633265	941.141	-0.002478008	957.407	-0.001268624	973.635	0.000577652
925.401	-0.000419092	941.703	-0.000666439	957.967	-0.003436087	974.193	0.001911866
925.963	0.000824701	942.264	0.001659805	958.527	-0.00330939	974.752	0.00051742
926.526	0.000643589	942.826	0.000335184	959.088	-0.001197357	975.311	-0.002030851
927.089	-0.000978279	943.387	-0.002186822	959.648	0.00059296	975.870	-0.003542804
927.651	-0.001701029	943.949	-0.001871964	960.208	0.001141148	976.428	-0.003694527
928.214	-0.00209355	944.510	-0.003742282	960.768	0.000475765	976.987	-0.001507779
928.777	-0.000416683	945.071	-0.00032716	961.328	-0.001252004	977.546	0.00157517
929.339	-1.29341E-05	945.632	-0.001702313	961.887	-0.002246418	978.104	0.003082207
929.902	0.000420469	946.193	0.000768766	962.447	-0.000911381	978.663	-0.001715285
930.464	-0.001839646	946.755	-0.000873592	963.007	0.000817383	979.221	-0.002471315
931.026	-0.00278642	947.316	-0.003292073	963.567	0.000939382	979.780	-0.004042772
931.589	-0.001850281	947.877	-0.001438545	964.127	-0.00048973	980.338	-0.001225085
932.151	0.000847651	948.438	-0.000563447	964.686	-0.001652686	980.896	0.000939429
932.713	0.002013148	948.999	0.001155371	965.246	-0.003493577	981.455	0.000470762
933.275	0.000473588	949.559	0.002005354	965.805	-0.003037451	982.013	-0.000944163
933.837	-0.00205406	950.120	-0.001306165	966.365	0.000126689	982.571	-0.00358881
934.400	-0.002056658	950.681	-0.003126266	966.924	0.001662419	983.129	-0.003950993
934.962	-0.002505767	951.242	-0.001917526	967.484	-4.65188E-05	983.687	-0.003412264
935.524	0.000562398	951.803	-0.002991177	968.043	-0.002229896	984.245	-0.000737397
936.086	0.000986198	952.363	-0.000894334	968.603	-0.004036274	984.803	0.001593525
936.648	-0.000382656	952.924	0.001240279	969.162	-0.003021829	985.361	-4.81492E-05
937.209	-0.001227332	953.484	9.21723E-05	969.721	-0.000118324	985.919	-0.001531018
937.771	-0.003680379	954.045	-0.000140019	970.280	0.001346066	986.477	-0.004732037
938.333	-0.002358329	954.605	-0.001063585	970.839	0.002503583	987.035	-0.004434991
938.895	0.000347752	955.166	-0.001877905	971.399	-8.43186E-05	987.593	-0.002206313
939.457	0.00218183	955.726	-0.000589975	971.958	-0.00420957	988.150	-0.000471613
940.018	-0.001146772	956.287	0.001377629	972.517	-0.003788772	988.708	0.000731341

Table S1. (continued) Raw data points of UV/Vis Measurement of the 8.5 x 10^{-8} M solution of [{SiN^{Dipp}}MgNa]$_2$ (**6**) in benzene.

**Wavelength (nm)**	**Absorbance**	**Wavelength (nm)**	**Absorbance**	**Wavelength (nm)**	**Absorbance**	**Wavelength (nm)**	**Absorbance**
989.266	0.000943891	992.053	0.003148771	994.839	-0.002463763	997.624	-0.003534015
989.823	-0.001096579	992.610	0.001853683	995.396	-0.001604604	998.181	-0.004974385
990.381	-0.00342335	993.168	0.000477883	995.954	0.000697618	998.738	-0.002316209
990.938	-0.001932477	993.725	-0.000639646	996.511	0.001075267	999.295	-0.000337665
991.496	-0.000268299	994.282	-0.002887548	997.068	-0.000952165	999.852	0.001347163

1.4. EPR Spectroscopy

The EPR spectrum of **6** consistently yielded evidence of multiple paramagnetic impurities, including one that appears to be characterised by a 1:2:1 triplet pattern, that were present in different relative amounts in each batch of starting material prepared (see Fig. S8). Upon addition of THF to these solutions, a grey powdered precipitate was immediately observed to form accompanied by loss of the yellow colour to the solution phase. The resulting EPR spectrum shows significant changes from the precursor **6**, including loss of the "triplet" pattern and the concomitant appearance of a new broad singlet characterised by g_{iso} = 2.0068, which persisted at room temperature for several days, which is tentatively assigned to atomic sodium. This assignment is based upon consideration of the viable SET processes and by comparison to literature reports on the free radical of Na, which lists g_{iso} = 2.0015.[5]

Figure S8. CW EPR spectra (T = 298 K) of benzene solutions of (a,b) [{SiN^{Dipp}}MgNa]$_2$ (**6**) prepared as different batches, and (c) following THF addition to (b). Experimental parameters: MW power = 10 mW; modulation amplitude = 0.4 mT; modulation frequency = 100 kHz. It should be noted that no evidence of hyperfine coupling was resolved using 0.1 mT modulation amplitude.

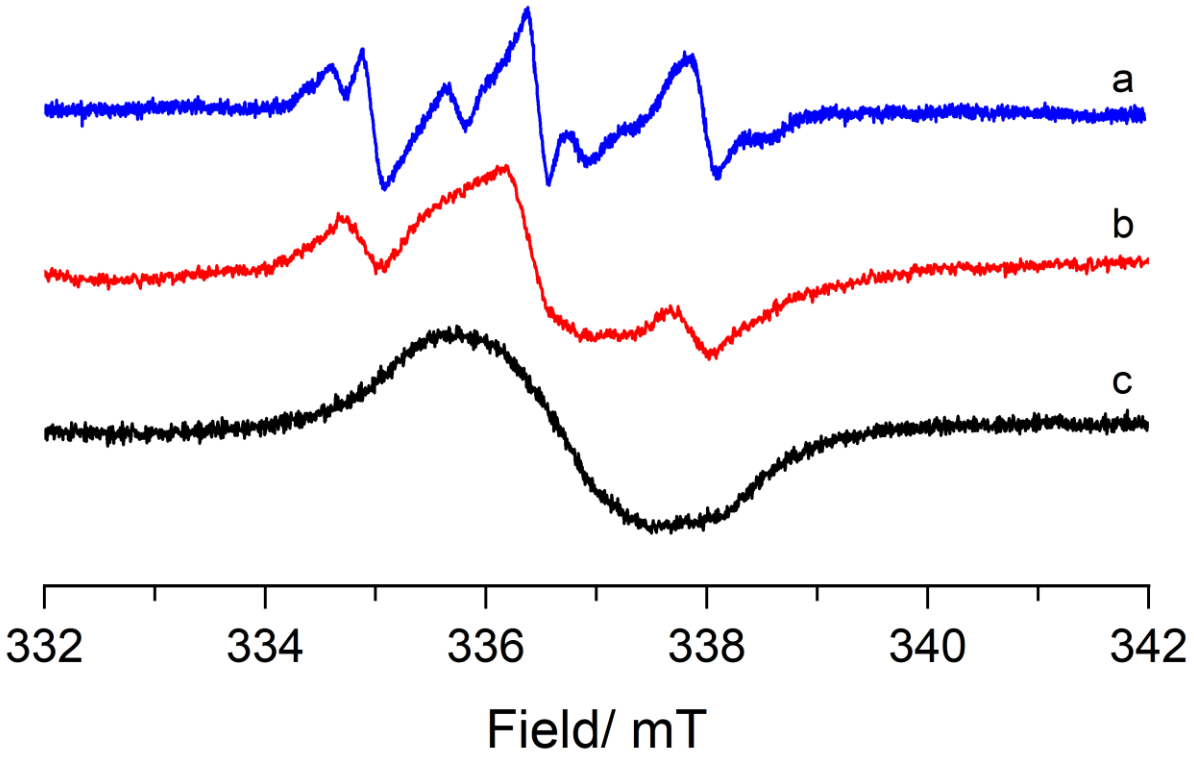

1.5. ICP-OES Details (Conducted at Butterworth Lab.)

In the glove box, [{SiN^{Dipp}}MgNa]$_2$ (**6**, 117.6mg, 0.1088 mmol) was dissolved in 5mL of toluene in a vial. A slight excess amount of tetrahydrofuran (20.0 μL, 17.78 mg, 0.2466 mmol) was then added to the bright yellow solution *via* a micropipette. The resultant solution was then carefully decanted and all of the resultant metallic powder/mirror was washed with toluene (0.5mL x2) and hexane (0.5mL x2). The powder was then collected, and all volatiles were removed under vacuum. The vial was then removed from the glovebox, 1 mL of deionised water was slowly added to the powder, bubbles were observed during the process. After a few minutes, several drops of HCl(aq.) were added before the solution was diluted with deionised water to 5 mL in volume. The sample was then stored under ambient conditions and shipped to Butterworth Lab. Ltd., Teddington, for ICPOES analysis.

BUTTERWORTH
LABORATORIES

Certificate of Analysis

Department of Chemistry
University of Bath
Bath
UK
BA2 7AY

**Job Ref:**	**2204-0037**
Report Ref:	RN-01090-22
Date Issued:	25 April 2022
Order Ref:	27504553

For attention of H. Liu

Sample of:	Acidified Aqueous Solution of Metallic Mirror		
**BLL Ref:**	**04-0134-22**	Date Received:	6 April 2022
Analysis Started:	12 April 2022	Analysis Completed:	25 April 2022
Sample Reference:	HYDL714		

Test	Result	Units
Magnesium expressed as Mg	<1	mg/L
	<1	mg/L
Sodium expressed as Na	1010	mg/L
	940	mg/L

Results relate to sample(s) as received.

Sample(s) analysed in accordance with in house method BLM 537G (revision 7).

Tim Goddard
Laboratory Manager
General & Inorganic Chemistry

Craig McConville
Analytical Operations Manager
Issued for and on behalf of Butterworth Laboratories

Encl. Raw Data Pack

1.6. Single Crystal X-ray Diffraction Analysis

Data were collected for compounds **7**, **8** and **10** on a SuperNova, Dual Cu at zero, EosS2 diffractometer (Cu Kα; λ = 1.54184 Å). The crystals were all maintained at 150(2) K during data collection. Using Olex2,[6] the structures were solved with the olex2.solve[7] structure solution program or ShelXT and refined with the ShelXL[8] refinement package using Least Squares minimization.

The asymmetric unit in **7** contains 2 molecules of benzene as well as one molecule of the cyclic magnesium-containing complex. C62, C63, C66 and C67 were successfully treated for 72:28 disorder, with the inclusion of distance and ADP restraints.

Half of a dimer unit constitutes the asymmetric unit in the structure of **8**, with the remainder of the molecule being generated by virtue of a crystallographic inversion centre.

The asymmetric unit in the extraordinary, 2-D, polymeric structure of **10** contains a motif based on two magnesium centres, four silicons and two sodium atoms and enough benzene to execute Xmas ablutions. Na2 was modelled to take account of 90:10 disorder, which is chemically sensible given the propensity for the alkali metals to 'slip' around rings to which they exhibit hapticity governed bonding. The solvent content manifested as two ordered benzene molecules plus four disordered moieties. In particular, the molecules respectively containing C91, C97, C103 and C109 were modelled to take account of each being split over proximate sites in disorder ratios of 50:50, 60:40, 60:40, and 60:40. Distance and ADP restraints were used in disordered regions, to assist convergence. The sample was twinned and this was addressed at the point of integration. While this twinning refined to approximately 8% of the diffraction, modelling same helped to improve convergence and reduce the residual electron-density in the difference Fourier electron-density map.

Table S2: Crystal data and structure refinement for compounds **7**, **8** and **10**.

Compound	**7**	**8**	**10**
Empirical formula	$C_{86}H_{134}Mg_2N_4O_2Si_4$	$C_{30}H_{50}MgN_2Si_2$	$C_{120}H_{156}Mg_2N_6Na_2Si_4$
Formula weight	1416.94	519.21	1889.46
Crystal system	monoclinic	monoclinic	monoclinic
Space group	$P2_1/c$	$P2_1/n$	$P2_1/n$
a/Å	18.4111(1)	17.1366(2)	19.88519(15)
b/Å	24.2048(1)	9.8013(1)	20.70181(18)
c/Å	20.3155(1)	20.3055(2)	27.6936(2)
α/°	90	90	90
β/°	103.599(1)	113.258(1)	95.8273(8)
γ/°	90	90	90
Volume/Å^3	8799.53(8)	3133.38(6)	11341.44(16)
Z	4	4	4
ρ_{calc} g/cm^3	1.070	1.101	1.107
μ/mm^{-1}	1.101	1.356	1.034
F(000)	3096.0	1136.0	4080.0
Crystal size/mm^3	0.2 × 0.17 × 0.115	0.165 × 0.156 ×	0.284 × 0.102 ×
2θ range /°	7.304 to 146.006	8.662 to 146.002	7.17 to 151.052
Index ranges	$-22 \le h \le 16$, $-30 \le k \le 29$, $-25 \le l \le 25$	$-21 \le h \le 21$, $-10 \le k \le 12$, $-25 \le l \le 25$	$-24 \le h \le 24$, $-25 \le k \le 25$, $-34 \le l \le 33$
Reflections collected	123836	42736	26999
Independent reflections	17517, 0.0500	6253, 0.0437	2699, 0.0664
Data/restraints/parameters	17517/154/943	6253/0/328	26999/368/1372
Goodness−of−fit on F^2	1.017	1.033	0.916
Final R indexes [$I \ge 2\sigma$] (I)]	R_1 = 0.0365,	R_1 = 0.0354,	R_1 = 0.0474,
Final R indexes [all data]	R_1 = 0.0439,	R_1 = 0.0396,	R_1 = 0.0724,
Largest diff. peak/hole (e Å^{-3})	0.32/–0.22	0.30/–0.23	0.24/–0.25

1.7 Computational Details

DFT calculations were run with Gaussian 16 (C.01).[9] The Na, Mg, Al and Si centres were described with the Stuttgart RECPs and associated basis sets,[10] and the 6-31G** basis set was used for all other atoms (BS1).[11] A polarisation function was also added to Al (ζ_d = 0.190) and Si (ζ_d = 0.284). Initial BP86 optimisations were performed using the 'grid = ultrafine' option,[12] with all stationary points being fully characterized via analytical frequency calculations as minima or transition states (all positive eigenvalues or one imaginary eigenvalue respectively). All energies were recomputed with a larger basis set featuring 6-311++G** basis sets on all atoms (BS2). Corrections for the effect of benzene (ε = 2.2706) solvent were run using the polarisable continuum model and BS1.[13] Single-point dispersion corrections to the BP86 results employed Grimme's D3 parameter set with Becke-Johnson damping as implemented in Gaussian.[14]

Quantum Theory of Atoms in Molecules (QTAIM) topological analysis of the electron densities of structures **7**, **10** and **B** were computed with AIMALL professional (version 19.10.12)[15] using wavefunction files obtained with Gaussian 16 (C.01) at the BP86/6-311++G** level. Natural Bonding Orbital (NBO7[16]) analyses were also performed on the BP86/6-311++G**-optimized geometries. Contour plots were generated in the AIMStudio package, with the critical point (CP) visualisation (denoted by solid lines) threshold set to 0.015 and the weak-CP threshold (denoted by dashed lines) set to 0.01.

The NBO energies of donor-acceptor interactions ("$\Delta E^{(2)}$") between the various molecular fragments (automatically designated during the NBO procedure itself) of the structures were estimated with second-order perturbation theory analysis of the Fock matrix in the NBO basis, as is performed in NBO7. Donor-acceptor NBO interactions above 1.5 kcal mol^{-1} between each designated fragment as part of the analyses are provided in Tables S11, S12 and S13.

TD-DFT calculations (time-dependent DFT) of **6** were carried out with ORCA 4.1.0 and 5.0.1.[17] The structure was pre-optimized with DFT at the BP86/def2-SVP level with the RI approximation, and subsequent single-point TD-DFT calculations were run at the CAM-B3LYP-D3BJ/def2-TZVP level in benzene (C_6H_6), for which the RIJCOX approximation was invoked, with 50 roots (nroots 50) and a Davidson Expansion setting of 5 (maxdim 5) employed.[18] When generating the simulated UV-Vis spectrum from the raw TD-DFT data via *map_spc*, a line width of 3000 was employed.

Breakdown of Energy Contributions

The following tables detail the evolution of the relative energies as the successive corrections to the initial SCF energy are included. Terms used are:

ΔE_{BSI}	SCF energy computed with the BP86 functional with BS1
ΔH_{BSI}	Enthalpy at 0 K with BS1
ΔG_{BSI}	Free energy at 298.15 K and 1 atm with BS1
$\Delta G_{BSI/bnz}$	Free energy corrected for benzene solvent with BS1
$\Delta G_{BSI/bnz+D3BJ}$	Free energy corrected for benzene and dispersion effects with BS1
ΔE_{BS2}	SCF energy computed with the BP86 functional with BS2
ΔG_{bnz}	Free energy corrected for basis set (BS2), dispersion effects and benzene solvent

In each case the final data used in the main article are highlighted in bold.

Table S3. Relative energies for computed structures. Data in bold are those used in the main text. Free energies are quoted relative to complex **6** at 0.0 kcal mol^{-1} and using a layer of $^{2}Na_{9}$ atoms to represent the metallic mirror precipitate and $[Na(THF)_4]^+$ to represent THF-solvated Na^+ in the calculation of the relative free energy of **F**.

	ΔE_{BSI}	ΔH_{BSI}	ΔG_{BSI}	$\Delta G_{BSI/bnz}$	$\Delta G_{BSI/bnz+D3BJ}$	ΔE_{BS2}	ΔG_{bnz}
**6**	0.0	0.0	0.0	0.0	0.0	0.0	**0.0**
**7**	−8.5	−9.1	0.7	−19.3	−23.6	−29.3	**−44.4**
**8**	31.6	27.7	7.7	−15.4	9.2	4.3	**−18.1**
**10**	−28.9	−25.8	−4.6	21.4	−7.2	−23.8	**−2.0**
**A**	0.8	0.4	3.6	4.5	17.2	−3.8	**12.5**
**TS(6-A)**	20.9	22.9	37.2	40.6	23.8	22.8	**25.7**
**B**	−1.3	−1.3	8.8	11.2	−1.3	1.5	**1.5**
**TS(6-B)**	−0.5	−0.7	9.1	11.6	2.6	2.1	**5.1**
**C**	2.8	3.6	19.8	23.8	−6.0	5.3	**−3.6**
**D** (or **8′**)	32.2	27.1	3.2	−20.5	16.7	4.3	**−11.2**
**E**	42.6	41.2	33.6	33.9	41.4	37.1	**35.9**
**F**	30.8	31.5	56.9	29.7	20.1	33.7	**23.0**

Additional Discussion on Computational Intermediates

To better understand the reactivity of **6**, we explored a range of different electronic "computational" scenarios. In the first, removal of both Na atoms from **6** to form the neutral {Mg_2} dimer (**C**) and reduced elemental Na to balance. Significant reorganisation of **C** was observed, with the 3.341 Å Mg–Mg bond of **6** increasing to 6.594 Å, affording an exergonic adduct with Mg···^{i}Pr interactions between a methyl group of the Dipp substituent on the other [Mg] monomer unit at −3.6 kcal mol^{-1} (relative to **6**).

Figure S9. Computed free energy of Na^0 abstraction of **6** to form **C**.

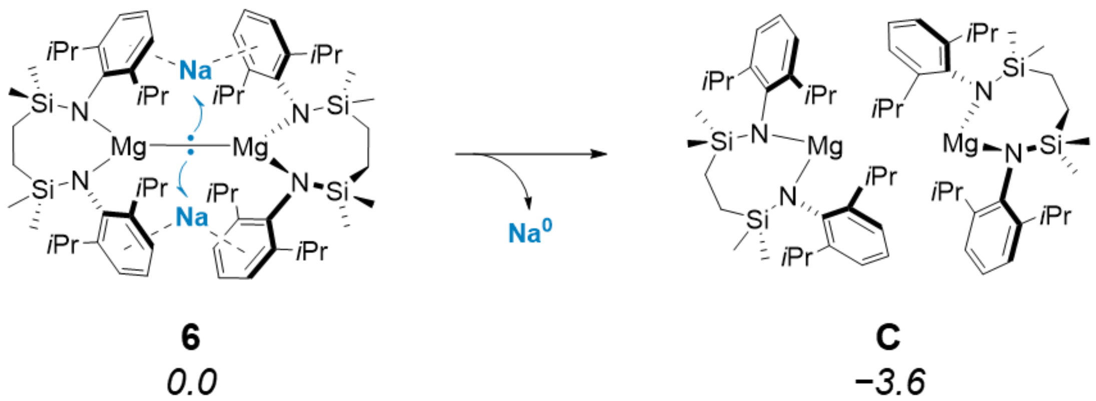

Comparison of this to the neutral 14-atom macrocycles formed by the addition of THF (complex **7**, here computationally referred to as **D** or **8′** after deletion of the THF molecules coordinated at the Mg centres) or a bulky base (**8**), sees increased Mg···Mg distances, of 7.765 and 6.122 Å respectively. Both macrocycles are exergonically formed, with free energies relative to **6** of −11.2 (**D**) and −18.1 (**8**) kcal mol^{-1}, with the two coordinate Mg(II) centres stabilised by Mg···H interactions of the disilazide carbon backbone and Si methyl groups. These interactions support the conformations of both macrocycles, which in **D** sees the backbone compressed between the Mg centres to facilitate four Mg···H_{SiMe} contacts of ~ 2.82 Å and two Mg···$H_{C\alpha}$ contacts of ~ 3.74 Å, and in **8** the unusual "slanted" off-set nature of the Mg centres is aided by a close Mg···$H_{C\beta}$ contact at each end of the macrocycle (Mg···$H_{C\beta}$ = 2.38-2.39 Å).

Figure S10. Computed free energy of rearrangement of **6** after Na^0 loss to form **D** or **8**.

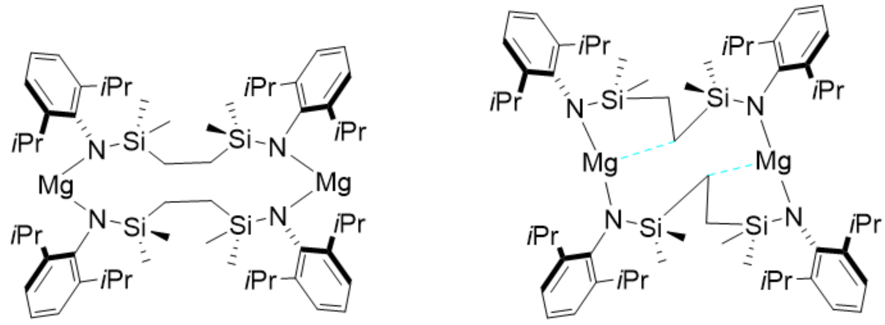

D *−11.2* **8** *−18.1*

Structures **C**, **D** and **8** consider the energetics and geometries when Na is reduced and Na(0) is lost concertedly. If this was a stepwise process, and only one Na(0) atom is lost initially from **6**, a neutral doublet dimer, $\{Mg_2Na\}^{\bullet}$ (**E**), is endergonically formed (ΔG_{bnz} = +35.9 kcal mol^{-1}). The Mg–Mg distance increases to 4.487 Å, and the remaining Na···Mg "triangle" distances also elongated from 3.950 to 4.180 Å. Equal distribution of spin density is observed, divided between the two Mg atoms with 0.47 e^- each, and 0.02 e^- at the remaining Na atom. Similarly, the stepwise loss of a Na(I) cation instead from **6**, gives the anionic $\{Mg_2Na\}^-$ structure, **F** (ΔG_{bnz} = +23.0 kcal mol^{-1}). Here the Mg–Mg distance is shortened to 3.256 Å by 0.07 Å, with the Mg···Na distances also reduced to 3.650 Å.

Figure S11. Computed free energies of Na^0 removal from **6** to form radical species **E**, and removal of $[Na(THF)_4]^+$ cation from **6** to form **F**.

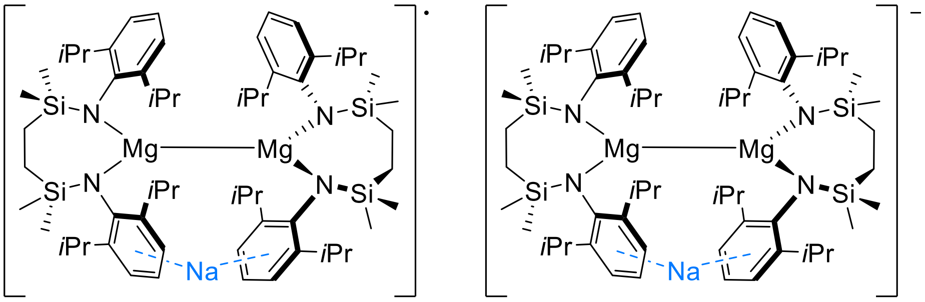

E *+35.9* **F** *+23.0*

Computational Description of a Na(0) Mirror

The formation of a Na(0) mirror in several of the reactions explored in the manuscript led us to explore different ways to model this by-product species. A group 1 (sodium) metallic mirror is formed after the reaction of compound **6** with different reagents. Whilst including a neutral computed metal atom balances the atomic and electronic equations for relative energies, it does fail to capture the implied solid and structural characteristics of a metal mirror.

Without wanting to completely change the computational methodology and introduce periodic boundary conditions, a range of different neutral sodium oligomers (Na_N) were computed and compared for sodium, [Ne]3s^1. These oligomers are atomic (N = 1), dimeric (N = 2), a rhombus (N = 4), a rhombohedron (N = 8) and a layer (N = 9) – see page S30. As sodium is a group 1 metal, it has one unpaired electron, therefore the singlet and N+1 electronic states were computed for each oligomer i.e. a doublet, triplet, quintet and nonet multiplicity respectively.

Table S4. Relative free energies (**ΔG_{bnz}**) for computed structures. Data in bold are those used in the main text. Free energies are quoted relative to complex **6** at 0.0 kcal mol^{-1} and using a layer of $^{X}Na_N$ atoms to represent the metallic mirror precipitate.

	^{2}Na	$^{1}Na_2$	$^{3}Na_2$-t	$^{1}Na_4$	$^{5}Na_4$	$^{1}Na_8$	$^{9}Na_8$	$^{2}Na_9$	$^{10}Na_9$
**7**	−16.5	−36.7	−16.8	−39.9	−24.1	−43.6	−22.5	**−44.4**	−14.2
**8**	9.8	−10.4	9.6	−13.6	2.2	−17.3	3.8	**−18.1**	12.1
**10**	11.9	1.9	11.8	0.2	8.2	−1.6	8.9	**−2.0**	13.1
**C**	16.6	−3.5	16.4	−6.8	9.1	−10.5	10.6	**−11.2**	18.9

Synthetically we know that complexes **7**, **8** and **10** crystallise, and therefore must be thermodynamically more stable than **6**. The free energies presented in Table S4 above suggest only the $^{1}Na_8$ and $^{2}Na_9$ oligomers are accurately capturing the reaction energetics of the sodium mirror. There is less than 1 kcal mol^{-1} difference between the inclusion of either an eight and nine atom oligomer, and so the latter was chosen for reporting free energies as it was felt the doublet nature of the nine atom (2D) layer of sodiums better represented the group 1 metal mirror, than the singlet eight atom (3D) rhombohedron.

Figure S12. Optimized structures of various ${}^{x}\mathbf{Na_y}$ clusters, along with selected interatomic distances.

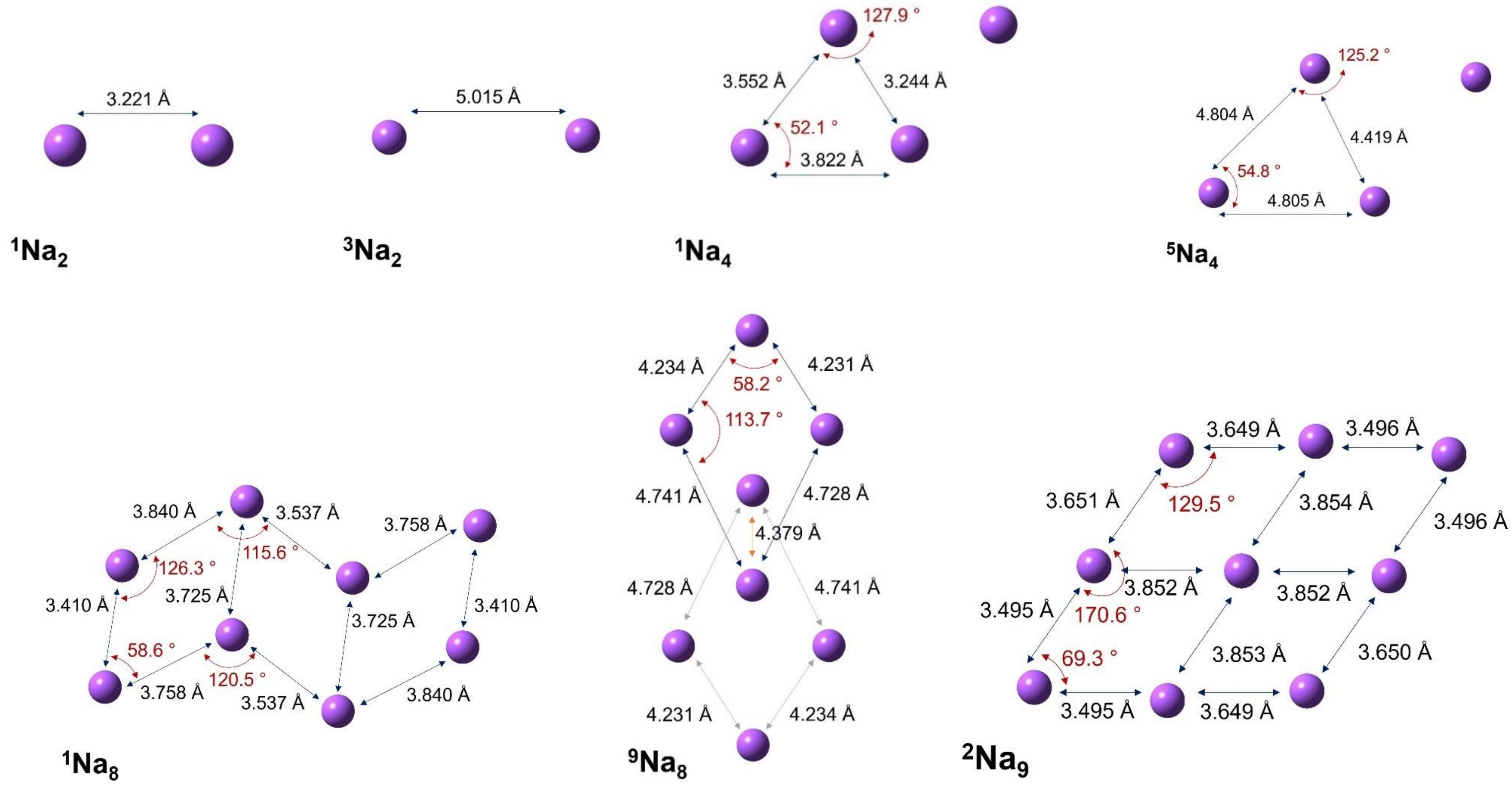

QTAIM Contour Plots & Tabulated Data

7

Figure S13. Contour plot of ρ(r) of the DFT-optimized structure of **7**

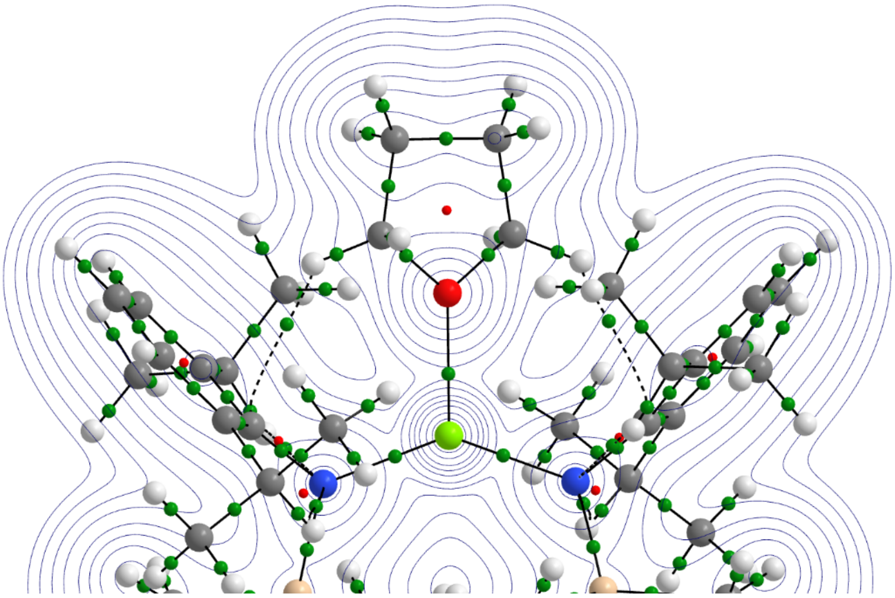

Figure S14. Contour plot of $\nabla^2\rho(r)$ of the DFT-optimized structure of **7**

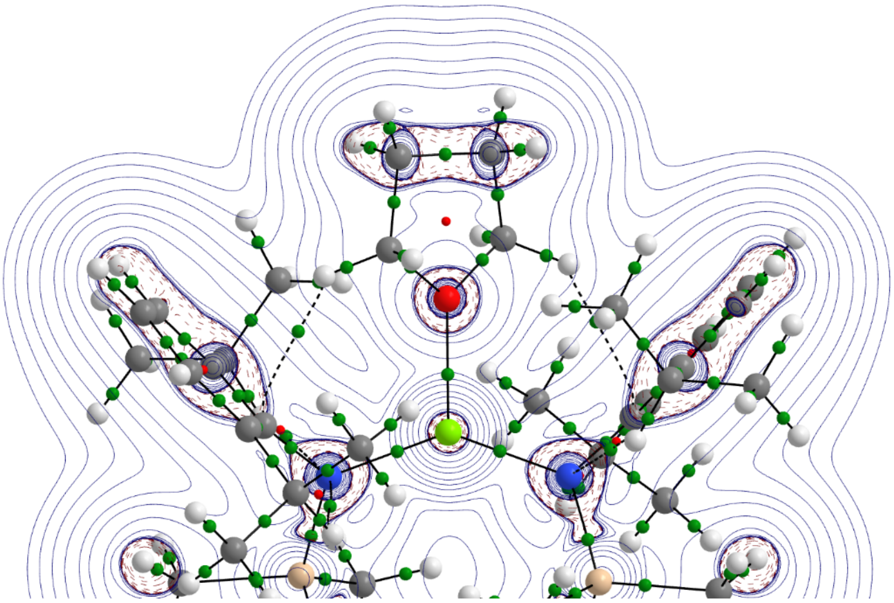

Table S5. BCP data for the DFT-optimised structure of **7**

BCP	ρ(r)	$\nabla^2\rho(r)$	ε	G(r)	V(r)	H(r)
Mg5 - O7	0.03463	0.23922	0.11733	-0.00879	-0.04222	-0.05101
Mg5 - N9	0.05377	0.33722	0.12850	-0.00519	-0.07393	-0.07912
Mg5 - N11	0.05378	0.33725	0.12851	-0.00519	-0.07394	-0.07913
Mg6 - O8	0.035409	0.247051	0.118583	-0.00907	-0.04362	-0.05269
Mg6 - N10	0.05413	0.340077	0.128925	-0.00514	-0.07474	-0.07988
Mg6 - N12	0.054133	0.340106	0.128926	-0.00514	-0.07475	-0.07989

Table S6. Selected AIM and NBO atomic data for **7**

Atom	L(r)	N(r)	Vol(r)	Loc(r)	$q_{AIM}(r)$	$q_{NBO}(r)$
Mg5	0.00012	10.31439	52.84352	96.38766	1.68561	1.84878
Mg6	-0.00013	10.31520	53.26394	96.37319	1.68480	1.84958
O7	0.00011	9.05751	91.98986	86.48695	-1.05751	-0.74039
O8	-0.00007	9.05985	91.21162	86.47067	-1.05985	-0.74210
N9	-0.00014	8.733952	126.3661	83.34771	-1.73395	-1.47637
N10	-0.0005	8.736251	126.6447	83.34408	-1.73625	-1.47218
N11	-0.00011	8.733936	126.3565	83.34836	-1.73394	-1.47171
N12	0.000009	8.735665	126.4634	83.34676	-1.73567	-1.47627

10

Figure S15. Contour plot of ρ(r) of the DFT-optimized structure of **10**.

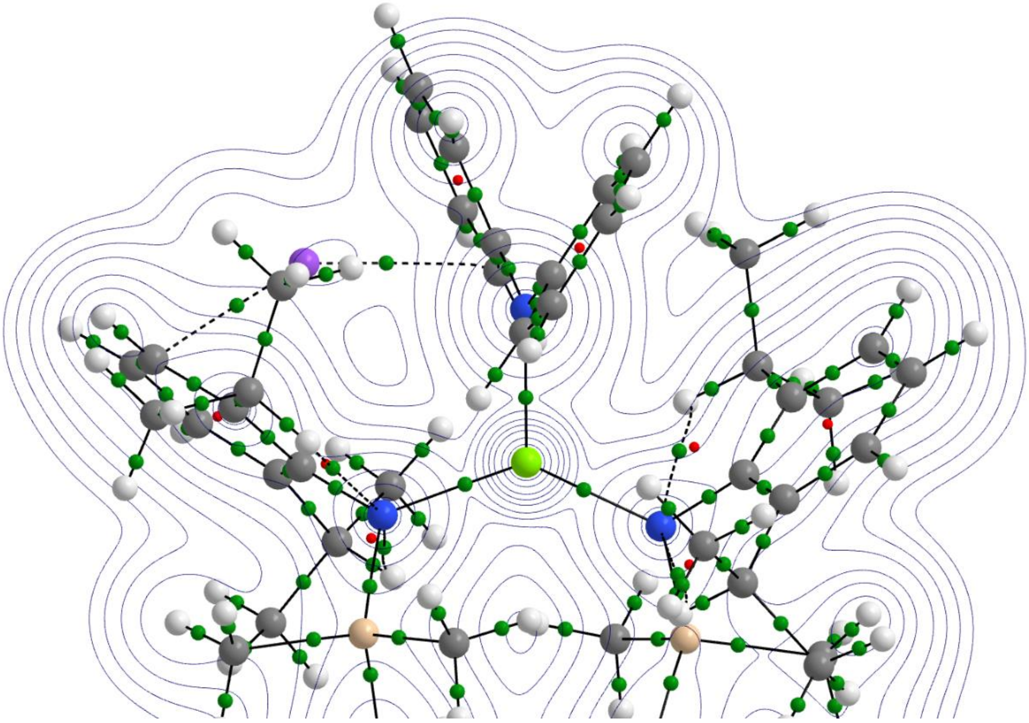

Figure S16. Contour plot of $\nabla^2\rho(r)$ of the DFT-optimized structure of **10**.

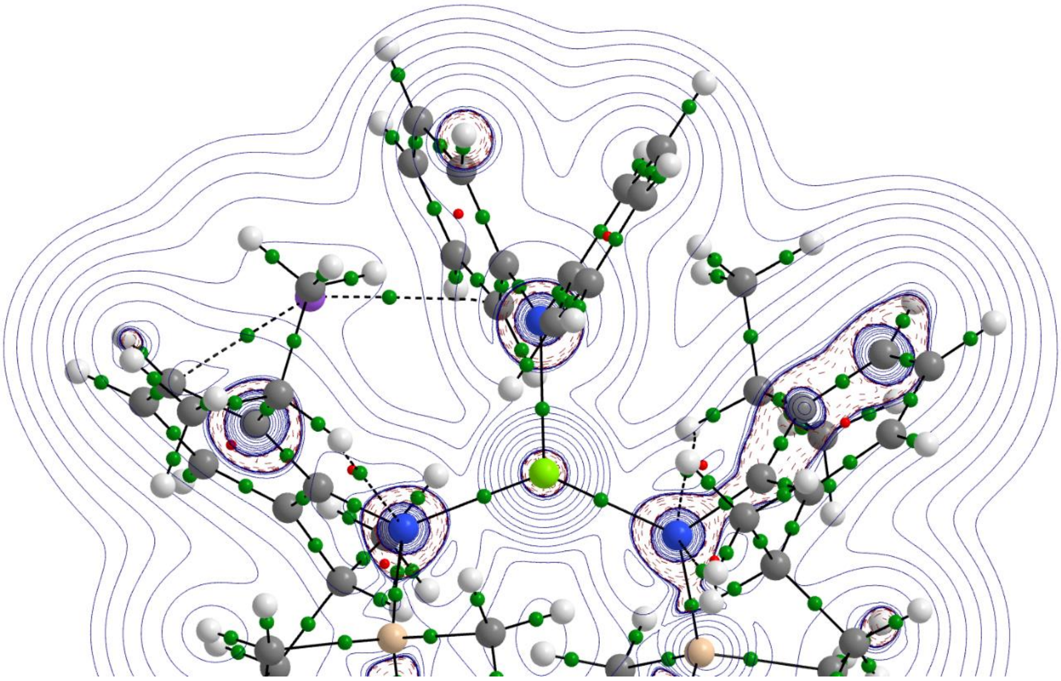

Table S7. BCP data for the DFT-optimized structure of **10**

BCP	ρ(r)	∇^2ρ(r)	ε	G(r)	V(r)	H(r)
Mg6 - N8	0.049809	0.305754	0.136059	0.071034	-0.06563	0.005404
Mg5 - N9	0.04981	0.305761	0.136062	0.071036	-0.06563	0.005405
Mg6 - N10	0.043293	0.25685	0.121653	0.058879	-0.05355	0.005334
Na7 - C105	0.012988	0.061194	3.635926	0.012755	-0.01021	0.002544
Mg6 - N13	0.042966	0.256449	0.096148	0.058247	-0.05238	0.005865
Mg5 - N11	0.042963	0.256422	0.096145	0.058241	-0.05238	0.005864
Mg5 - N12	0.043296	0.256875	0.121655	0.058885	-0.05355	0.005334
Na7 - C17	0.01271	0.05916	2.042744	0.012364	-0.00994	0.002426
C84 - Na218	0.012699	0.059104	2.048465	0.012352	-0.00993	0.002424
C92 - Na218	0.012988	0.061194	3.641136	0.012754	-0.01021	0.002544

Table S8. Selected AIM and NBO atomic data for **10**

Atom	L(r)	N(r)	Vol(r)	Loc(r)	q_{AIM}(r)	q_{NBO}(r)
Mg5	0.000148	10.32125	51.16312	96.36459	1.678752	1.81442
Mg6	-0.00012	10.32166	51.23772	96.36104	1.678341	1.80798
Na7	0.000007	10.11882	75.8575	98.17609	0.881178	0.92201
N8	-0.00005	8.733441	125.5933	83.37225	-1.73344	-1.45476
N9	0.000061	8.73329	125.5324	83.37308	-1.73329	-1.45506
N10	-0.00032	8.693876	123.7728	83.14561	-1.69388	-1.39657
N11	-0.00023	8.215932	100.7718	79.54197	-1.21593	-0.85085
N12	-0.00027	8.69387	123.7915	83.14717	-1.69387	-1.39482
N13	0.000447	8.21541	100.7305	79.54476	-1.21541	-0.84997
Na218	0.000045	10.11874	75.82453	98.17712	0.881259	0.92162

B

Figure S17. Contour plot of ρ(r) of the DFT-optimized structure of **B**

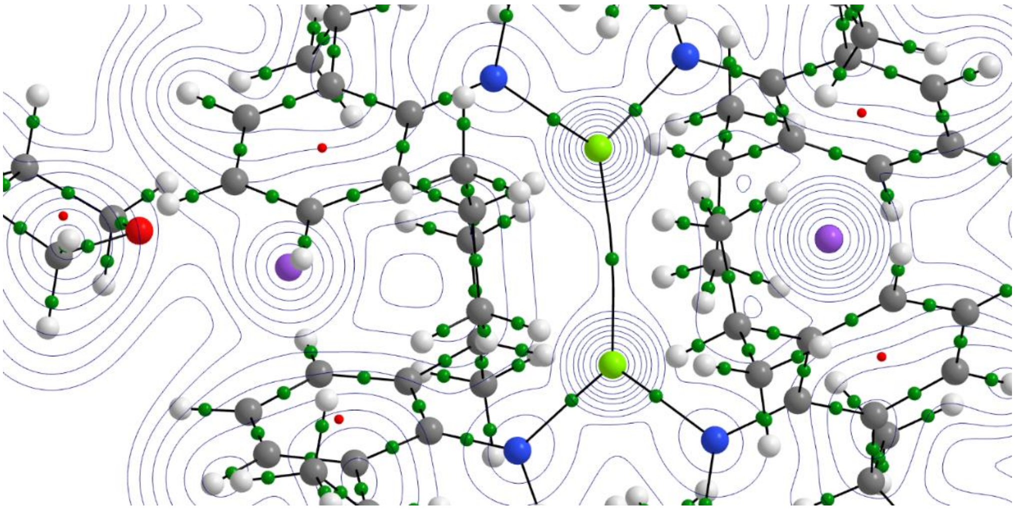

Figure S18. Contour plot of $\nabla^2\rho(r)$ of the DFT-optimized structure of **B**

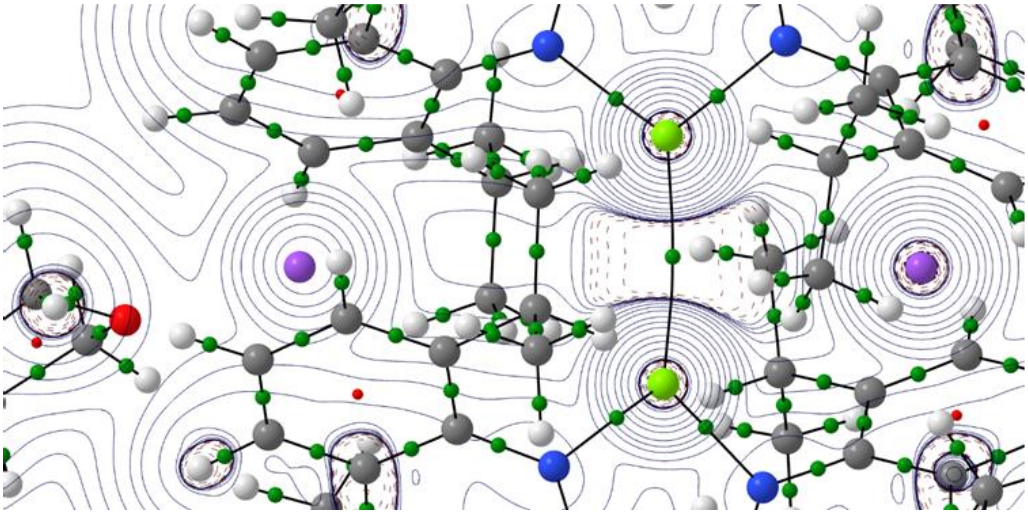

Table S9. BCP data for the DFT-optimised structure of **B**

BCP	ρ(r)	∇^2ρ(r)	ε	G(r)	V(r)	H(r)
Mg5 - Mg6	0.020607	-0.01545	0.051901	-0.00437	0.000254	-0.00412
Mg5 - Na8	0.005043	0.007405	0.376248	-0.00166	0.001753	0.000098
Na8 - C30	0.010958	0.047405	9.929499	-0.00798	0.009916	0.001935
Na7 - C31	0.011255	0.050215	0.989806	-0.00823	0.010393	0.002161
Na8 - C22	0.011058	0.048033	5.567988	-0.0081	0.010056	0.001952
Na7 - C69	0.010633	0.048922	1.718001	-0.00791	0.010071	0.00216
Na7 - O173	0.013188	0.074161	0.148728	-0.01226	0.015399	0.003141

Table S10. Selected AIM and NBO atomic data for **B**

Atom	L(r)	N(r)	Vol(r)	Loc(r)	q_{AIM}(r)	q_{NBO}(r)
Mg5	-0.00048	11.08187	158.024178	93.14101	0.918135	1.01210
Mg6	0.000183	11.0012	146.35007	93.30444	0.998801	1.02466
Na7	0.000254	10.10047	77.641329	98.43838	0.899527	0.88869
Na8	0.000023	10.14276	75.370937	98.02768	0.857241	0.80396
N9	0.000015	8.700899	124.4659	82.94369	-1.7009	-1.38472
N10	-0.00012	8.70645	123.0499	82.88146	-1.70645	-1.38446
N11	-8.2E-05	8.704331	123.3283	82.91374	-1.70433	-1.38527
N12	0.000198	8.694635	125.1646	82.95665	-1.69464	-1.37980
O173	-0.000038	8.992612	96.937191	86.28798	-0.99261	-0.62230

Second Order Perturbation Theory Analysis of Donor Acceptor NBO interactions between molecular fragments

Table S11. Selected donor acceptor interaction energies, $\Delta E^{(2)}$, for **7.**

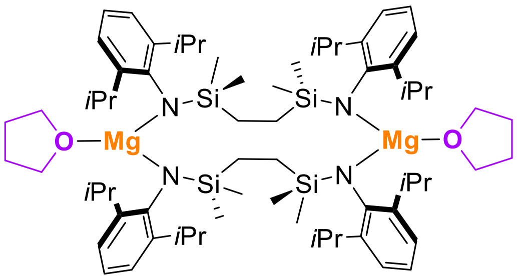

*Top & bottom {SiN^{Dipp}} (**1**&**2**) = Black*
*Left & right Mg (**3**&**4**) = Orange*
*Left & right H$^-$ (**5**&**6**) = Purple*

Donor NBO (Unit)	Acceptor NBO (Unit)	$\Delta E^{(2)}$ (kcal/mol)
n_{N11} (1)	n^*_{Mg5} (3)	5.2
$\Omega_{Si4\text{-}N11}$ (1)	n^*_{Mg5} (3)	2.4
$\Omega_{C21\text{-}C27}$ (1)	n^*_{Mg5} (3)	1.7
$\Omega_{C21\text{-}C29}$ (1)	n^*_{Mg5} (3)	1.6
$\Omega_{C141\text{-}H142}$ (1)	n^*_{Mg5} (3)	1.6
n_{N9} (2)	n^*_{Mg5} (3)	5.2
$\Omega_{Si2\text{-}N 9}$ (2)	n^*_{Mg5} (3)	2.5
$\Omega_{C13\text{-}C28}$ (2)	n^*_{Mg5} (3)	1.8
$\Omega_{C13\text{-}C35}$ (2)	n^*_{Mg5} (3)	1.7
$\Omega_{C178\text{-}H180}$ (2)	n^*_{Mg5} (3)	1.6
$\Omega_{N9\text{-}C13}$ (2)	n^*_{Mg5} (3)	1.6
n_{O7} (5)	n^*_{Mg5} (3)	6.6

Donor NBO (Unit)	Acceptor NBO (Unit)	$\Delta E^{(2)}$ (kcal/mol)
n_{N12} (1)	n^*_{Mg6} (4)	5.2
$\Omega_{Si1\text{-}N12}$ (1)	n^*_{Mg6} (4)	2.5
$\Omega_{C17\text{-}C22}$ (1)	n^*_{Mg6} (4)	1.6
$\Omega_{C17\text{-}C34}$ (1)	n^*_{Mg6} (4)	1.6
$\Omega_{N12\text{-}C17}$ (1)	n^*_{Mg6} (4)	1.5
n_{N10} (2)	n^*_{Mg6} (4)	5.2
$\Omega_{Si3\text{-}N10}$ (2)	n^*_{Mg6} (4)	2.4
$\Omega_{C23\text{-}C74}$ (2)	n^*_{Mg6} (4)	1.6
$\Omega_{C23\text{-}C56}$ (2)	n^*_{Mg6} (4)	1.5
$\Omega_{N10\text{-}C23}$ (2)	n^*_{Mg6} (4)	1.5
n_{O8} (6)	n^*_{Mg6} (4)	6.5
$\Omega_{C129\text{-}H130}$ (6)	n^*_{Mg6} (4)	1.7
$\Omega_{C138\text{-}H139}$ (6)	n^*_{Mg6} (4)	1.7

Table S12. Selected donor acceptor interaction energies, $\Delta E^{(2)}$, for **10.**

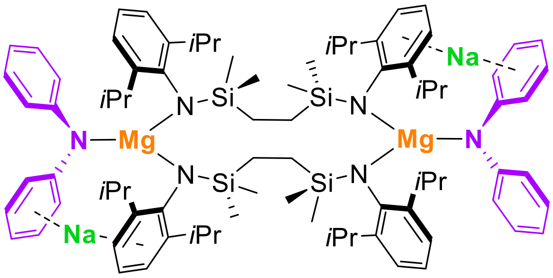

*Top & bottom {SiN^{Dipp}} (**1**&**2**) = Black*
*Left & right Mg (**3**&**4**) = Orange*
*Left & right Na (**5**&**8**) = Green*
*Left & right NPh_2 (**6**&**7**) = Purple*

Donor NBO (Unit)	Acceptor NBO (Unit)	$\Delta E^{(2)}$ (kcal/mol)
n_{N12} (1)	n^*_{Mg5} (3)	8.2
n_{N12} (1)	n^*_{Mg5} (3)	3.4
$\Omega_{Si2\text{-}N12}$ (1)	n^*_{Mg5} (3)	2.6
$\Omega_{N12\text{-}C19}$ (1)	n^*_{Mg5} (3)	1.5
n_{N9} (2)	n^*_{Mg5} (3)	9.5
$\Omega_{Si3\text{-}N9}$ (2)	n^*_{Mg5} (3)	3.3
$\Omega_{N9\text{-}C16}$ (2)	n^*_{Mg5} (3)	1.5
n_{N11} (6)	n^*_{Mg5} (3)	10.6
$\Omega_{C86\text{-}H87}$ (6)	n^*_{Mg5} (3)	2.0
$\Omega_{C84\text{-}H85}$ (6)	n^*_{Mg5} (3)	1.6
$\Omega_{N11\text{-}C38}$ (6)	n^*_{Mg5} (3)	1.6
$\Omega_{C64\text{-}C105}$ (2)	n^*_{Na7} (5)	1.7
$\Omega_{C25\text{-}C129}$ (2)	n^*_{Na7} (5)	1.6
$\Omega_{C17\text{-}C21}$ (7)	n^*_{Na7} (5)	1.7

Donor NBO (Unit)	Acceptor NBO (Unit)	$\Delta E^{(2)}$ (kcal/mol)
n_{N10} (2)	n^*_{Mg6} (4)	8.1
n_{N10} (2)	n^*_{Mg6} (4)	3.4
$\Omega_{Si4\text{-}N10}$ (2)	n^*_{Mg6} (4)	2.6
$\Omega_{N10\text{-}C33}$ (2)	n^*_{Mg6} (4)	1.5
n_{N8} (1)	n^*_{Mg6} (4)	9.5
$\Omega_{Si1\text{-}N8}$ (1)	n^*_{Mg6} (4)	3.3
$\Omega_{N8\text{-}C15}$ (1)	n^*_{Mg6} (4)	1.6
n_{N13} (7)	n^*_{Mg6} (4)	10.6
$\Omega_{C67\text{-}H68}$ (7)	n^*_{Mg6} (4)	2.0
$\Omega_{C17\text{-}H18}$ (7)	n^*_{Mg6} (4)	1.6
$\Omega_{N13\text{-}C24}$ (7)	n^*_{Mg6} (4)	1.6
$\Omega_{C31\text{-}C92}$ (1)	n^*_{Na218} (8)	1.7
$\Omega_{C26\text{-}C111}$ (1)	n^*_{Na218} (8)	1.6
$\Omega_{C47\text{-}C84}$ (6)	n^*_{Na218} (8)	1.7

Table S13. Selected donor acceptor interaction energies, $\Delta E^{(2)}$, for **B.**

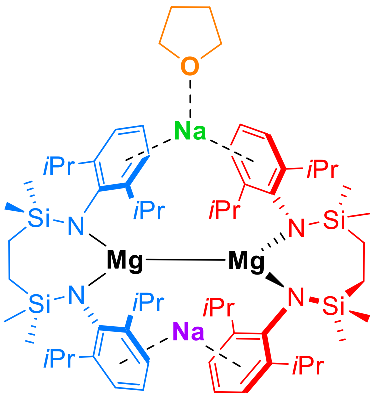

*Mg-Mg bond (**1**) = Black*
*Left {SiN^{Dipp}} (**2**) = Blue*
*Top Na (**3**) = Green*
*Right {SiN^{Dipp}} (**4**) = Red*
*Bottom Na (**5**) = Purple*
*THF (**6**) = orange*

**Donor NBO (Unit)**	**Acceptor NBO (Unit)**	**$\Delta E^{(2)}$ (kcal/mol)**
$\Omega_{Mg5\text{-}Mg6}$ (1)	n^*_{Na7} (3)	5.7
n_{O173} (6)	n^*_{Na7} (3)	2.2
$\Omega_{C21\text{-}C59}$ (2)	n^*_{Na7} (3)	1.9
$\Omega_{C20\text{-}C47}$ (2)	n^*_{Na7} (3)	1.8
$\Omega_{C14\text{-}C20}$ (2)	n^*_{Na7} (3)	1.7
$\Omega_{C14\text{-}C21}$ (2)	n^*_{Na7} (3)	1.7
$\Omega_{C69\text{-}H70}$ (2)	n^*_{Na7} (3)	1.7
$\Omega_{C59\text{-}H60}$ (2)	n^*_{Na7} (3)	1.6
$\Omega_{C47\text{-}H48}$ (2)	n^*_{Na7} (3)	1.6
$\Omega_{C16\text{-}C31}$ (4)	n^*_{Na7} (3)	2.0
$\Omega_{C49\text{-}H503}$ (4)	n^*_{Na7} (3)	1.9
$\Omega_{C31\text{-}H32}$ (4)	n^*_{Na7} (3)	1.4
$\Omega_{C13\text{-}C16}$ (4)	n^*_{Na7} (3)	1.6
$\Omega_{C18\text{-}C23}$ (4)	n^*_{Na7} (3)	1.5

**Donor NBO (Unit)**	**Acceptor NBO (Unit)**	**$\Delta E^{(2)}$ (kcal/mol)**
$\Omega_{Mg5\text{-}Mg6}$ (1)	n^*_{Na8} (5)	31.6
$\Omega_{C30\text{-}C55}$ (4)	n^*_{Na8} (5)	2.4
$\Omega_{C15\text{-}C17}$ (4)	n^*_{Na8} (5)	2.4
$\Omega_{C17\text{-}C30}$ (4)	n^*_{Na8} (5)	2.3
$\Omega_{C15\text{-}C33}$ (4)	n^*_{Na8} (5)	2.1
$\Omega_{C75\text{-}H76}$ (4)	n^*_{Na8} (5)	1.9
$\Omega_{C30\text{-}C61}$ (4)	n^*_{Na8} (5)	1.8
$\Omega_{C55\text{-}H56}$ (4)	n^*_{Na8} (5)	1.8
$\Omega_{C33\text{-}H34}$ (4)	n^*_{Na8} (5)	1.6
$\Omega_{C15\text{-}C63}$ (4)	n^*_{Na8} (5)	1.6
$\Omega_{C55\text{-}C75}$ (4)	n^*_{Na8} (5)	1.5
$\Omega_{C22\text{-}C51}$ (2)	n^*_{Na8} (5)	2.4
$\Omega_{C19\text{-}C43}$ (2)	n^*_{Na8} (5)	2.4
$\Omega_{C19\text{-}C22}$ (2)	n^*_{Na8} (5)	2.3
$\Omega_{C43\text{-}C67}$ (2)	n^*_{Na8} (5)	2.1
$\Omega_{C57\text{-}H58}$ (2)	n^*_{Na8} (5)	1.9
$\Omega_{C22\text{-}C65}$ (2)	n^*_{Na8} (5)	1.8
$\Omega_{C51\text{-}H52}$ (2)	n^*_{Na8} (5)	1.8
$\Omega_{C67\text{-}H68}$ (2)	n^*_{Na8} (5)	1.6
$\Omega_{C43\text{-}C99}$ (2)	n^*_{Na8} (5)	1.5
$\Omega_{C51\text{-}C57}$ (2)	n^*_{Na8} (5)	1.5

Table S14. Relative free energies for computed structures related to the transformation of compound **5** to compound **8**. Data in bold are those used in the main text. Free energies are quoted relative to complex **5** at 0.0 kcal mol^{-1}.

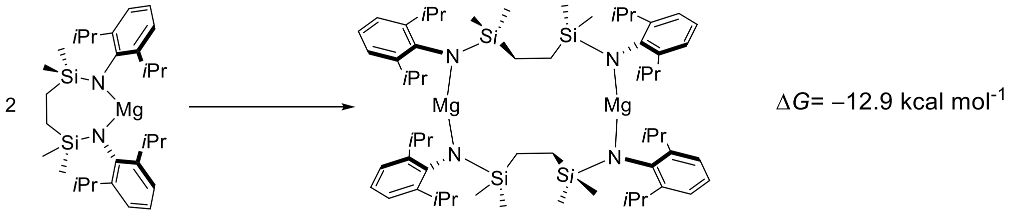

	ΔE_{BSI}	ΔH_{BSI}	ΔG_{BSI}	$\Delta G_{BSI/bnz}$	$\Delta G_{BSI/bnz+D3BJ}$	ΔE_{BS2}	ΔG_{bnz}
**5**	0.0	0.0	0.0	0.0	0.0	0.0	**0.0**
**8**	-2.9	-2.2	12.5	14.2	-15.8	-0.1	**-12.9**

References

[1] H. -Y. Liu, R. J. Schwamm, S. E. Neale, M. S. Hill, C. L. McMullin, M. F. Mahon, *J. Am. Chem. Soc.* **2021**, *143*, 17851–17856.

[2] C. Glock, H. Görls, M. Westerhausen, *Eur. J. Inorg. Chem*.**2011**, 5288–5298

[3] N. Kuhn, T. Kratz, *Synthesis* **1993**, *6*, 561.

[4] R. J. Schwamm, M. P. Coles, M. S. Hill, M. F. Mahon, C. L. McMullin, N. A. Rajabi, A. S. S. Wilson, *Angew. Chem. Int. Ed*. **2020**, *59*, 3928.

[5] G. Feher and A. F. Kip, *Phys. Rev.* (1955), 98, 337-348.

[6] Dolomanov, O. V.; Bourhis, L.J.; Gildea, R.J.; Howard, J. A. K.; Puschmann, H. *J. Appl. Cryst*. **2009**, *42*, 339-341.

[7] G. M. Sheldrick, *Acta Cryst*. **2015**, *A71*, 3-8.

[8] G. M. Sheldrick, *Acta Cryst*. **2015**, *C71*, 3-8.

[9] M. J. Frisch, G. W. Trucks, H. B. Schlegel, G. E. Scuseria, M. A. Robb, J. R. Cheeseman, G. Scalmani, V. Barone, G. A. Petersson, H. Nakatsuji, X. Li, M. Caricato, A. V. Marenich, J. Bloino, B. G. Janesko, R. Gomperts, B. Mennucci, H. P. Hratchian, J. V. Ortiz, A. F. Izmaylov, J. L. Sonnenberg, Williams, F. Ding, F. Lipparini, F. Egidi, J. Goings, B. Peng, A. Petrone, T. Henderson, D. Ranasinghe, V. G. Zakrzewski, J. Gao, N. Rega, G. Zheng, W. Liang, M. Hada, M. Ehara, K. Toyota, R. Fukuda, J. Hasegawa, M. Ishida, T. Nakajima, Y. Honda, O. Kitao, H. Nakai, T. Vreven, K. Throssell, J. A. Montgomery Jr., J. E. Peralta, F. Ogliaro, M. J. Bearpark, J. J. Heyd, E. N. Brothers, K. N. Kudin, V. N. Staroverov, T. A. Keith, R. Kobayashi, J. Normand, K. Raghavachari, A. P. Rendell, J. C. Burant, S. S. Iyengar, J. Tomasi, M. Cossi, J. M. Millam, M. Klene, C. Adamo, R. Cammi, J. W. Ochterski, R. L. Martin, K. Morokuma, O. Farkas, J. B. Foresman, D. J. Fox, Wallingford, CT, 2016.

[10] D. Andrae, U. Häußermann, M. Dolg, H. Stoll, H. Preuß, *Theor. Chim. Acta* 1990, 77, 123-141.

[11] (a) P. C. Hariharan, J. A. Pople, *Theor. Chim. Acta* 1973, 28, 213-222; (b) W. J. Hehre, R. Ditchfield, J. A. Pople, *J. Chem. Phys.* 1972, 56, 2257-2261.

[12] (a) A. D. Becke, *Phys. Rev. A* 1988, 38, 3098-3100; (b) J. P. Perdew, *Phys. Rev. B* 1986, 33, 8822-8824.

[13] J. Tomasi, B. Mennucci, R. Cammi, *Chem. Rev.* 2005, 105, 2999-3094.

[14] S. Grimme, S. Ehrlich, L. Goerigk, *J. Comp. Chem.* 2011, 32, 1456-1465.

[15] AIMAll (Version 19.10.12), T. A. Keith, TK Gristmill Software, Overland Park KS, USA, 2019 (aim.tkgristmill.com).

[16] NBO 7.0, E. D. Glendening, J. K. Badenhoop, A. E. Reed, J. E. Carpenter, J. A. Bohmann, C. M. Morales, P. Karafiloglou, C. R. Landis, and F. Weinhold, Theoretical Chemistry Institute, University of Wisconsin, Madison, USA, 2003.

[17] (a) The ORCA program system, F. Neese, *Wiley Interdiscip. Rev.: Comput. Mol. Sci.* 2012, 2, 73-78; (b) Software update: the ORCA program system, version 4.0, F. Nesse, *Wiley Interdiscip. Rev.: Comput. Mol. Sci.* 2017, 8, e1327; (c) Software update: the ORCA program system, version 5.0, F. Nesse, *Wiley Interdiscip. Rev.: Comput. Mol. Sci.* 2022, e1606.

[18] T. Yanai, D. P. Tew, N. C. Handy, *Chem. Phys. Lett.* 2004, 393 51-57.

Cartesian Coordinates and Computed Energies (in Hartrees) for Calculated Structures

Substrates

THF

SCF (BP86) Energy = -232.447574761
Enthalpy 0K = -232.333967
Enthalpy 298K = -232.327978
Free Energy 298K = -232.363078
Lowest Frequency = 23.1459 cm^{-1}
Second Frequency = 275.4150 cm^{-1}
SCF (BP86-D3BJ) Energy = -232.461052284
SCF (C6H6) Energy = -232.448881463
SCF (BS2) Energy = -232.511411715

```
C   1.11953  0.49016  0.18507
O  -0.01624  1.21238 -0.30202
C  -1.14796  0.45256  0.13889
C  -0.75507 -1.04217 -0.02233
C   0.80213 -0.99935 -0.08328
H   2.01008  0.86631 -0.34439
H   1.25822  0.66776  1.27604
H  -1.37418  0.67882  1.20507
H  -2.01389  0.75889 -0.47051
H  -1.12708 -1.64979  0.81904
H  -1.17361 -1.46775 -0.94858
H   1.27911 -1.66696  0.65304
H   1.15943 -1.29360 -1.08366
```

$NaNPh_2$

SCF (BP86) Energy = -518.261459390
Enthalpy 0K = -518.081561
Enthalpy 298K = -518.068454
Free Energy 298K = -518.121084
Lowest Frequency = 45.1418 cm^{-1}
Second Frequency = 59.6806 cm^{-1}
SCF (BP86-D3BJ) Energy = -518.309935068
SCF (C6H6) Energy = -518.285565747
SCF (BS2) Energy = -680.481195272

```
N  -0.05748  0.79707  0.29725
C   1.10532  0.05901  0.18814
C   2.30651  0.60146  0.77145
C   1.27984 -1.13787 -0.58714
C   3.57005  0.02349  0.56009
H   2.20032  1.41372  1.50961
C   2.54365 -1.69685 -0.78894
H   0.40220 -1.59356 -1.05502
C   3.70801 -1.12672 -0.23076
H   4.44856  0.46544  1.04619
H   2.62821 -2.59941 -1.40683
H   4.68923 -1.58239 -0.39396
C  -1.32135  0.23956  0.16844
C  -2.39320  1.07253 -0.26717
C  -1.66404 -1.09910  0.52709
C  -3.70621  0.60071 -0.36630
H  -2.16688  2.11877 -0.51955
C  -2.98056 -1.56502  0.42389
H  -0.88294 -1.76130  0.91386
C  -4.01666 -0.72894 -0.02883
H  -4.49711  1.27829 -0.70993
H  -3.20274 -2.59904  0.71415
H  -5.04315 -1.10087 -0.10673
Na  1.02168  2.62975 -0.39020
```

$^{-}NPh_2$

SCF (BP86) Energy = -518.067889993
Enthalpy 0K = -517.890201
Enthalpy 298K = -517.878935
Free Energy 298K = -517.926646
Lowest Frequency = 58.7496 cm^{-1}
Second Frequency = 64.4739 cm^{-1}
SCF (BP86-D3BJ) Energy = -518.110935879
SCF (C6H6) Energy = -518.109487273
SCF (BS2) Energy = -518.211476658

```
N  -0.00001 -1.12611  0.00034
C  -1.20294 -0.48276  0.00732
C  -2.35699 -1.20738 -0.45725
C  -1.48093  0.82965  0.53598
C  -3.64564 -0.67249 -0.41631
H  -2.17911 -2.21659 -0.84847
C  -2.77934  1.34973  0.57940
H  -0.65077  1.41688  0.94318
C  -3.88489  0.62014  0.09837
H  -4.48673 -1.27276 -0.79224
H  -2.93573  2.35166  1.00563
H  -4.89725  1.03989  0.12906
C   1.20291 -0.48275 -0.00705
C   2.35717 -1.20737  0.45715
C   1.48080  0.82972 -0.53570
C   3.64578 -0.67247  0.41592
H   2.17937 -2.21662  0.84831
C   2.77920  1.34981 -0.57939
H   0.65058  1.41699 -0.94270
C   3.88489  0.62023 -0.09869
H   4.48696 -1.27280  0.79156
H   2.93544  2.35178 -1.00557
H   4.89722  1.04003 -0.12960
```

Complexes

5

SCF (BP86) Energy = -1291.60521301
Enthalpy 0K = -1290.892021
Enthalpy 298K = -1290.845210
Free Energy 298K = -1290.973061
Lowest Frequency = 12.1095 cm^{-1}
Second Frequency = 15.8740 cm^{-1}
SCF (BP86-D3BJ) Energy = -1291.81348434
SCF (C6H6) Energy = -1291.61448887
SCF (BS2) Energy = -2062.42200384

Si -2.18872 -0.68198 2.04556
Si 2.18876 0.68155 2.04564
N -1.92507 -0.19623 0.36531
N 1.92506 0.19618 0.36530
C -0.39411 -0.67944 2.78276
H -0.51106 -1.04028 3.82079
H 0.19737 -1.49527 2.30677
C 0.39409 0.67931 2.78271
H 0.51099 1.04027 3.82070
H -0.19738 1.49514 2.30665
C -2.88985 -2.43728 2.29745
H -3.89590 -2.52922 1.85453
H -2.97884 -2.66727 3.37418
H -2.24882 -3.20705 1.83573
C -3.28930 0.52242 3.03082
H -2.79320 1.49232 3.20117
H -3.55666 0.09722 4.01375
H -4.22685 0.71769 2.48259
C 3.28886 -0.52332 3.03085
H 2.79262 -1.49323 3.20071
H 3.55592 -0.09846 4.01401
H 4.22659 -0.71849 2.48289
C 2.89051 2.43656 2.29783
H 3.89651 2.52823 1.85473
H 2.97979 2.66630 3.37459
H 2.24968 3.20666 1.83638
C -2.97824 0.14691 -0.53562
C -3.40535 1.51010 -0.66546
C -4.43437 1.83245 -1.56972
H -4.75908 2.87471 -1.66306
C -5.05078 0.85108 -2.35579
H -5.85297 1.12059 -3.05102
C -4.61784 -0.47501 -2.25089
H -5.08155 -1.24281 -2.88140
C -3.59356 -0.84887 -1.35942
C -2.69805 2.62343 0.10725
H -2.24712 2.15436 0.99921
C -3.63152 3.74979 0.59273
H -4.04747 4.33659 -0.24522
H -3.07811 4.45571 1.23664
H -4.47769 3.34711 1.17393
C -1.54219 3.20996 -0.73766
H -0.82581 2.42737 -1.05794
H -0.97517 3.97389 -0.17582
H -1.92734 3.67704 -1.66099
C -3.10675 -2.29752 -1.35155
H -2.43775 -2.40321 -0.48085
C -2.27129 -2.59381 -2.61958
H -2.88258 -2.48110 -3.53255
H -1.87278 -3.62403 -2.60282
H -1.41685 -1.89946 -2.71153
C -4.24989 -3.32368 -1.20575
H -4.86224 -3.12317 -0.31050
H -3.84361 -4.34706 -1.12049
H -4.92606 -3.31276 -2.07904
C 2.97824 -0.14680 -0.53567
C 3.40537 -1.50996 -0.66573
C 4.43447 -1.83213 -1.56998
H 4.75919 -2.87436 -1.66349
C 5.05091 -0.85060 -2.35583
H 5.85318 -1.11997 -3.05104
C 4.61794 0.47546 -2.25073
H 5.08168 1.24338 -2.88107
C 3.59359 0.84913 -1.35927
C 2.69806 -2.62346 0.10672
H 2.24685 -2.15453 0.99861
C 1.54247 -3.21008 -0.73852
H 0.82602 -2.42756 -1.05878
H 0.97550 -3.97420 -0.17689
H 1.92790 -3.67696 -1.66183
C 3.63159 -3.74972 0.59231
H 3.07814 -4.45580 1.23601
H 4.47756 -3.34697 1.17376
H 4.04784 -4.33636 -0.24560
C 3.10664 2.29774 -1.35123
H 2.43786 2.40335 -0.48035
C 2.27077 2.59389 -2.61904
H 2.88185 2.48124 -3.53216
H 1.87211 3.62405 -2.60220
H 1.41643 1.89941 -2.71075
C 4.24965 3.32408 -1.20575
H 4.86228 3.12366 -0.31067
H 3.84322 4.34738 -1.12036
H 4.92558 3.31329 -2.07922
Mg -0.00005 0.00043 0.18738

6

SCF (BP86) Energy = -2583.71039948
Enthalpy 0K = -2582.279802
Enthalpy 298K = -2582.183208
Free Energy 298K = -2582.408854
Lowest Frequency = 25.7364 cm^{-1}
Second Frequency = 28.7482 cm^{-1}
SCF (BP86-D3BJ) Energy = -2584.21464
SCF (C6H6) Energy = -2583.72981115
SCF (BS2) Energy = -4449.50396798

Si -4.45184 1.03190 -1.67121
Si -4.45163 -1.03146 1.67150
Si 4.45180 1.03159 1.67122
Si 4.45149 -1.03176 -1.67146
Mg -1.67056 0.00005 -0.00001
Mg 1.67047 0.00003 -0.00000
Na 0.00006 3.57971 -0.00018
Na -0.00005 -3.58080 -0.00021
N -2.87354 1.46130 -0.96293
N -2.87352 -1.46113 0.96294
N 2.87338 -1.46138 -0.96285
N 2.87361 1.46121 0.96280
C -2.38599 2.75195 -1.23361
C 2.38620 2.75191 1.23356
C -1.49473 -3.00862 2.35218
C -2.74038 3.89500 -0.41100
C -2.38612 -2.75182 1.23371
C -1.49480 3.00877 -2.35223
C 2.38596 -2.75204 -1.23364
C 1.49501 3.00877 2.35216
C 2.74074 3.89497 0.41102
C 2.74058 -3.89514 -0.41119
C -0.99752 4.30977 -2.58654
H -0.34223 4.47888 -3.44896
C -1.14036 1.90911 -3.35358

H	-1.47784	0.96016	-2.90055
C	5.83195	-0.66617	-0.38945
H	5.85852	-1.52031	0.31493
H	6.77321	-0.75224	-0.97342
C	-2.74090	-3.89495	0.41137
C	-2.21246	5.17458	-0.69148
H	-2.51012	6.01981	-0.05906
C	-0.99762	-4.30967	2.58661
H	-0.34218	-4.47873	3.44893
C	5.83207	0.66584	0.38905
H	5.85872	1.52000	-0.31530
H	6.77340	0.75176	0.97296
C	-5.83209	0.66640	-0.38892
H	-5.85854	1.52051	0.31550
H	-6.77346	0.75248	-0.97272
C	1.14047	1.90913	3.35349
H	1.47796	0.96017	2.90051
C	1.49473	-3.00886	-2.35223
C	-5.83206	-0.66567	0.38949
H	-5.85874	-1.51976	-0.31495
H	-6.77331	-0.75170	0.97349
C	0.99781	4.30980	2.58650
H	0.34250	4.47892	3.44889
C	-1.33890	5.39826	-1.76752
H	-0.94971	6.40013	-1.97579
C	2.21290	-5.17477	-0.69184
H	2.51082	-6.02006	-0.05961
C	3.72430	3.76481	-0.75086
H	3.98977	2.69557	-0.80357
C	-2.21314	-5.17458	0.69198
H	-2.51112	-6.01987	0.05978
C	1.33932	-5.39846	-1.76787
H	0.95040	-6.40041	-1.97631
C	2.21293	5.17457	0.69154
H	2.51070	6.01980	0.05916
C	-3.72460	-3.76491	-0.75040
H	-3.99020	-2.69571	-0.80309
C	-1.13984	-1.90888	3.35328
H	-1.47749	-0.95996	2.90033
C	3.72408	-3.76502	0.75074
H	3.98943	-2.69575	0.80359
C	0.99766	-4.30992	-2.58669
H	0.34235	-4.47902	-3.44910
C	1.33932	5.39828	1.76753
H	0.95025	6.40019	1.97584
C	4.26324	0.48766	-2.81657
H	3.70870	0.21706	-3.73127
H	5.24900	0.88291	-3.12151
H	3.71380	1.30452	-2.32096
C	-1.33939	-5.39824	1.76787
H	-0.95040	-6.40017	1.97626
C	-4.26396	-0.48761	-2.81625
H	-3.70910	-0.21719	-3.73081
H	-5.24981	-0.88245	-3.12145
H	-3.71496	-1.30471	-2.32053
C	-5.17458	2.45537	-2.73884
H	-5.86558	3.08610	-2.15432
H	-5.75547	2.02896	-3.57588
H	-4.40360	3.11699	-3.16460
C	4.26368	-0.48772	2.81649
H	3.70889	-0.21702	3.73101
H	5.24946	-0.88268	3.12175
H	3.71452	-1.30482	2.32096
C	-3.72394	3.76491	0.75087
H	-3.98928	2.69565	0.80372
C	5.17472	2.45508	2.73868
H	5.86546	3.08592	2.15395
H	5.75594	2.02873	3.57551
H	4.40380	3.11659	3.16470
C	-1.89971	2.10500	-4.68787
H	-2.99064	2.12758	-4.54025
H	-1.66715	1.28390	-5.38959
H	-1.60674	3.05321	-5.17419
C	1.13995	-1.90914	-3.35340
H	1.47756	-0.96022	-2.90044
C	-4.26332	0.48786	2.81673
H	-3.70844	0.21722	3.73121
H	-5.24905	0.88289	3.12206
H	-3.71418	1.30490	2.32108
C	0.37175	1.80689	-3.62903
H	0.77893	2.73105	-4.07654
H	0.58378	0.98767	-4.33702
H	0.93477	1.59551	-2.70170
C	1.89970	2.10504	4.68785
H	2.99064	2.12764	4.54033
H	1.66710	1.28393	5.38955
H	1.60667	3.05324	5.17415
C	0.37240	-1.80671	3.62804
H	0.77970	-2.73082	4.07550
H	0.58480	-0.98738	4.33579
H	0.93500	-1.59550	2.70040
C	-0.37167	1.80696	3.62883
H	-0.77885	2.73114	4.07628
H	-0.58378	0.98776	4.33682
H	-0.93462	1.59556	2.70145
C	-5.17449	-2.45484	2.73908
H	-5.86351	-3.08713	2.15392
H	-5.75752	-2.02834	3.57457
H	-4.40341	-3.11497	3.16697
C	-0.37227	-1.80698	-3.62829
H	-0.77957	-2.73117	-4.07561
H	-0.58460	-0.98778	-4.33621
H	-0.93493	-1.59559	-2.70075
C	5.17425	-2.45512	-2.73913
H	5.86331	-3.08744	-2.15405
H	5.75724	-2.02859	-3.57464
H	4.40315	-3.11523	-3.16699
C	3.10209	-4.17901	2.10181
H	2.19766	-3.59212	2.33575
H	3.82034	-4.01923	2.92494
H	2.82277	-5.24852	2.10903
C	5.01744	4.57462	-0.50291
H	4.81099	5.65986	-0.46925
H	5.74414	4.39925	-1.31564
H	5.49466	4.29711	0.44989
C	-5.01717	4.57452	0.50272
H	-4.81082	5.65978	0.46879
H	-5.74385	4.39928	1.31550
H	-5.49436	4.29671	-0.45000
C	3.10231	4.17855	-2.10201
H	2.19798	3.59150	-2.33592
H	3.82062	4.01875	-2.92509
H	2.82284	5.24801	-2.10938
C	-3.10209	4.17895	2.10199
H	-2.19762	3.59214	2.33600
H	-3.82040	4.01908	2.92505
H	-2.82287	5.24848	2.10927
C	-3.10264	-4.17856	-2.10159
H	-2.19840	-3.59138	-2.33554
H	-3.82101	-4.01887	-2.92463
H	-2.82303	-5.24799	-2.10896
C	-1.89853	-2.10463	4.68796
H	-2.98954	-2.12708	4.54092
H	-1.66553	-1.28352	5.38952
H	-1.60542	-3.05284	5.17419
C	1.89874	-2.10494	-4.68803

```
H   2.98974 -2.12729 -4.54092
H   1.66571 -1.28389 -5.38965
H   1.60573 -3.05321 -5.17420
C   5.01733 -4.57467  0.50282
H   4.81097 -5.65993  0.46897
H   5.74390 -4.39937  1.31568
H   5.49468 -4.29697 -0.44985
C  -5.01763 -4.57490 -0.50245
H  -4.81105 -5.66013 -0.46885
H  -5.74436 -4.39958 -1.31516
H  -5.49490 -4.29750  0.45036
```

7

SCF (BP86) Energy = -3048.17407086
Enthalpy 0K = -3046.514154
Enthalpy 298K = -3046.407557
Free Energy 298K = -3046.663397
Lowest Frequency = 8.7168 cm^{-1}
Second Frequency = 12.7275 cm^{-1}
SCF (BP86-D3BJ) Energy = -3048.71141400
SCF (C6H6) Energy = -3048.18772206
SCF (BS2) Energy = -4589.92041907

```
Si  2.12715  2.25342 -0.98618
Si -2.13576 -2.24346 -0.98757
Si  2.12727 -2.25334  0.98625
Si -2.13592  2.24356  0.98749
Mg -4.43250 -0.00007 -0.00012
Mg  4.41179  0.00002 -0.00012
O  -6.52523 -0.00008 -0.00052
O   6.49536  0.00018 -0.00031
N  -3.74182 -1.85146 -0.32666
N   3.73121 -1.85158  0.32970
N  -3.74187  1.85135  0.32647
N   3.73117  1.85164 -0.32982
C  -4.60430 -2.90266  0.10436
C   0.71514  1.97148  0.29402
H   0.89090  1.02513  0.83731
H   0.83029  2.77108  1.05458
C   4.61912  2.88635  0.08871
C  -0.72230 -1.96949  0.29284
H  -0.89387 -1.02444  0.83945
H  -0.84024 -2.77138  1.05069
C  -4.60461  2.90252 -0.10416
C   5.47943  3.55306 -0.85371
C   4.61916 -2.88639 -0.08862
C  -0.72233  1.96951 -0.29275
H  -0.89383  1.02445 -0.83937
H  -0.84024  2.77138 -1.05062
C  -4.69283  3.26554 -1.49243
C  -4.69276 -3.26494  1.49281
C  -5.45809  3.59185  0.82842
C   1.81702 -1.16477  2.52645
H   2.44029 -1.50865  3.37005
H   0.76158 -1.20332  2.84574
H   2.06600 -0.10525  2.33993
C   4.72431  3.25508  1.47402
C  -5.45737 -3.59275 -0.82804
C  -2.00650 -4.07578 -1.50665
H  -2.42776 -4.73974 -0.73314
H  -0.94673 -4.35164 -1.64596
H  -2.53096 -4.28184 -2.45332
C  -3.77639  2.62780 -2.53659
H  -3.05429  2.00635 -1.97764
C   1.81697  1.16487 -2.52641
H   2.44102  1.50833 -3.36962
H   0.76177  1.20409 -2.84640
H   2.06516  0.10520 -2.33970
C  -5.41196 -3.28487 -2.32597
H  -4.48927 -2.70564 -2.50121
C   2.00553 -4.08951  1.49238
H   2.42254 -4.74582  0.70999
H   0.94770 -4.36962  1.63783
H   2.53856 -4.30211  2.43283
C  -1.83216 -1.14708 -2.52305
H  -2.45585 -1.49106 -3.36634
H  -0.77714 -1.18167 -2.84421
H  -2.08406 -0.08903 -2.33343
C   5.47959 -3.55278  0.85390
C   0.71518 -1.97140 -0.29390
H   0.89089 -1.02504 -0.83717
H   0.83036 -2.77098 -1.05448
C  -5.41276  3.28340  2.32623
H  -4.49011  2.70405  2.50127
C   5.40923  3.24474 -2.35079
H   4.47326  2.68344 -2.51338
C   2.00542  4.08958 -1.49240
H   2.42240  4.74593 -0.71004
H   0.94761  4.36974 -1.63795
H   2.53853  4.30209 -2.43283
C   6.43571  4.47905 -0.39393
H   7.09563  4.96827 -1.12057
C  -1.83213  1.14766  2.52326
H  -2.45641  1.49125  3.36627
H  -0.77725  1.18319  2.84479
H  -2.08306  0.08936  2.33372
C   4.72426 -3.25547 -1.47384
C   6.56621  4.79123  0.96658
H   7.31943  5.51204  1.30258
C   3.78042  2.66759  2.52431
H   3.06023  2.03467  1.97573
C  -5.64307 -4.21582  1.91437
H  -5.70717 -4.46874  2.97993
C   5.70132  4.18386  1.88395
H   5.77649  4.44377  2.94726
C  -7.38082  0.96050 -0.73692
H  -7.32560  0.69923 -1.80918
H  -6.97105  1.96737 -0.57409
C  -2.00690  4.07599  1.50616
H  -2.42681  4.73984  0.73182
H  -0.94723  4.35153  1.64691
H  -2.53265  4.28256  2.45201
C   5.40956 -3.24406  2.35091
H   4.47369 -2.68257  2.51342
C  -5.64330  4.21638 -1.91371
H  -5.70720  4.46987 -2.97914
C  -6.38961  4.53694  0.35677
H  -7.04659  5.04126  1.07558
C  -6.38867 -4.53791 -0.35613
H  -7.04531 -5.04284 -1.07481
C  -7.38113 -0.96119  0.73498
H  -7.32545 -0.70109  1.80747
H  -6.97185 -1.96809  0.57097
C   3.78032 -2.66823 -2.52422
H   3.06019 -2.03513 -1.97576
C  -3.77670 -2.62646  2.53684
H  -3.05459 -2.00516  1.97772
C  -2.97261  3.68181 -3.32900
H  -3.63152  4.33021 -3.93378
H  -2.26460  3.19168 -4.02039
H  -2.39536  4.33414 -2.65248
C  -6.50013  4.84857 -1.00535
H  -7.23438  5.58450 -1.35028
C  -6.49944 -4.84882  1.00615
H  -7.23353 -5.58480  1.35129
C  -6.59601 -2.38681 -2.75060
```

```
H  -7.56109 -2.89771 -2.57762
H  -6.53829 -2.13065 -3.82382
H  -6.60198 -1.44770 -2.17210
C   5.37567  4.51424 -3.22823
H   4.57614  5.20441 -2.91255
H   5.20296  4.24683 -4.28583
H   6.32997  5.06930 -3.18597
C  -6.59689  2.38534  2.75059
H  -7.56190  2.89650  2.57800
H  -6.53908  2.12866  3.82367
H  -6.60314  1.44652  2.17162
C  -5.36787  4.55169  3.20493
H  -4.55179  5.22750  2.90098
H  -5.21456  4.28033  4.26450
H  -6.31137  5.12388  3.15033
C   7.35060  0.95154  0.74705
H   6.94353  1.03472  1.76658
H   7.28033  1.93201  0.24880
C   6.56977  2.32424 -2.79237
H   7.54696  2.81530 -2.63067
H   6.49299  2.07218 -3.86531
H   6.56406  1.38305 -2.21689
C   6.43589 -4.47883  0.39428
H   7.09591 -4.96779  1.12101
C   7.35042 -0.95157 -0.74733
H   6.94292 -1.03550 -1.76661
H   7.28059 -1.93175 -0.24843
C  -4.55142  1.70155 -3.50090
H  -5.07007  0.88607 -2.96211
H  -3.87107  1.23120 -4.23244
H  -5.32086  2.26052 -4.06321
C   5.37595 -4.51334  3.22866
H   6.33015 -5.06856  3.18632
H   4.57627 -5.20347  2.91328
H   5.20348 -4.24568  4.28623
C   4.51606  1.77163  3.54580
H   5.29147  2.33615  4.09425
H   5.01667  0.91417  3.05762
H   3.81179  1.35734  4.28859
C   5.70127 -4.18431 -1.88360
H   5.77638 -4.44449 -2.94685
C   6.57031 -2.32363  2.79214
H   6.49373 -2.07136  3.86505
H   6.56465 -1.38254  2.21651
H   7.54741 -2.81486  2.63038
C   6.56628 -4.79139 -0.96614
H   7.31950 -5.51225 -1.30202
C  -5.36717 -4.55344 -3.20424
H  -4.55117 -5.22922 -2.90003
H  -5.21378 -4.28244 -4.26389
H  -6.31074 -5.12551 -3.14953
C   2.97672  3.76774  3.25248
H   2.24710  3.32022  3.95037
H   2.42407  4.39770  2.53542
H   3.63487  4.43265  3.83989
C  -8.78581  0.75062 -0.17471
H  -8.93029  1.36184  0.73265
H  -9.56251  1.02763 -0.90527
C  -8.78621 -0.75023  0.17328
H  -8.93150 -1.36132 -0.73404
H  -9.56282 -1.02675  0.90413
C   8.75495 -0.35151 -0.68631
H   9.53159 -1.12777 -0.77454
H   8.90281  0.38292 -1.49665
C  -4.55211 -1.69989  3.50052
H  -5.32156 -2.25872  4.06294
H  -5.07080 -0.88476  2.96124
H  -3.87199 -1.22904  4.23197
C   4.51590 -1.77259 -3.54602
H   5.01669 -0.91507 -3.05813
H   3.81156 -1.35838 -4.28880
H   5.29116 -2.33731 -4.09448
C   2.97653 -3.76856 -3.25202
H   3.63463 -4.43368 -3.83926
H   2.24689 -3.32122 -3.95002
H   2.42389 -4.39827 -2.53474
C   8.75524  0.35177  0.68515
H   9.53174  1.12822  0.77294
H   8.90377 -0.38263  1.49540
C  -2.97285 -3.67991  3.32994
H  -2.26510 -3.18929  4.02123
H  -2.39532 -4.33243  2.65384
H  -3.63174 -4.32817  3.93489
```

8

SCF (BP86) Energy = -2583.21506930
Enthalpy 0K = -2581.787560
Enthalpy 298K = -2581.692939
Free Energy 298K = -2581.926206
Lowest Frequency = 5.2480 cm^{-1}
Second Frequency = 11.9552 cm^{-1}
SCF (BP86-D3BJ) Energy = -2583.67942344
SCF (C6H6) Energy = -2583.23093528
SCF (BS2) Energy = -4124.84406324

```
Si -3.62705  2.25092  1.85148
Si  1.00069  1.74564  1.67274
Mg -3.02588 -0.08830 -0.00172
N  -4.33793  1.22620  0.60451
N   2.28040  1.72918  0.46380
C  -5.59776  1.50282 -0.01517
C  -6.82005  1.02118  0.55870
C  -8.03857  1.26399 -0.10450
H  -8.96731  0.88437  0.33755
C  -8.09296  1.96829 -1.31202
H  -9.05208  2.14571 -1.81016
C  -6.90197  2.43935 -1.87584
H  -6.93725  2.98695 -2.82484
C  -5.65778  2.22590 -1.25301
C  -6.84086  0.20919  1.85475
H  -5.82163  0.24894  2.27326
C  -7.81678  0.79309  2.90016
H  -7.62390  1.86259  3.08875
H  -7.72570  0.25322  3.85924
H  -8.86735  0.70097  2.57165
C  -7.17409 -1.27713  1.58838
H  -8.17128 -1.38299  1.12449
H  -7.17991 -1.85118  2.53219
H  -6.43971 -1.74747  0.91378
C  -4.38900  2.71878 -1.94931
H  -3.57692  2.67510 -1.20148
C  -4.48527  4.17674 -2.44434
H  -5.23575  4.29248 -3.24604
H  -3.51565  4.50899 -2.85529
H  -4.76244  4.86010 -1.62477
C  -4.00514  1.77674 -3.11460
H  -3.87647  0.73094 -2.77568
H  -3.06401  2.09520 -3.59838
H  -4.79565  1.76259 -3.88565
C  -4.46680  2.22325  3.56428
H  -5.50008  2.60415  3.51482
H  -3.90908  2.87086  4.26451
H  -4.50290  1.20862  3.99510
C  -3.47876  4.08350  1.34654
H  -2.80684  4.23322  0.48516
H  -3.10557  4.69815  2.18401
```

H	-4.47159	4.47373	1.06322
C	-1.86402	1.50592	2.11683
H	-1.49514	2.04141	3.01412
H	-1.94067	0.45560	2.47184
C	-0.80838	1.62211	0.97304
H	-1.00121	2.49881	0.32692
H	-0.79808	0.73136	0.30025
C	1.25411	0.15492	2.70589
H	1.22326	-0.76235	2.08459
H	0.45284	0.03588	3.45517
H	2.21473	0.17421	3.24864
C	1.05803	3.30428	2.76319
H	1.88379	3.24279	3.49018
H	0.12466	3.46590	3.32679
H	1.23137	4.19182	2.13148
C	2.80992	2.92649	-0.12659
C	2.30735	3.41456	-1.37542
C	2.87269	4.56817	-1.95223
H	2.48254	4.93082	-2.91070
C	3.92210	5.25571	-1.33426
H	4.34955	6.15132	-1.79730
C	4.42338	4.77665	-0.11876
H	5.25410	5.30465	0.36339
C	3.89230	3.62955	0.50195
C	1.19777	2.68671	-2.13230
H	0.84572	1.87203	-1.47386
C	1.74082	2.04502	-3.43022
H	0.95066	1.48103	-3.95636
H	2.57515	1.35193	-3.21951
H	2.12628	2.81391	-4.12273
C	-0.00517	3.60376	-2.44123
H	0.27377	4.42531	-3.12427
H	-0.40941	4.06292	-1.52285
H	-0.81659	3.03370	-2.92882
C	4.52883	3.14346	1.80614
H	3.86172	2.36612	2.21997
C	4.66760	4.26161	2.86250
H	5.03412	3.84436	3.81704
H	3.70597	4.76589	3.05292
H	5.39204	5.03313	2.54723
C	5.90542	2.48891	1.54322
H	6.60626	3.21641	1.09703
H	5.84073	1.63349	0.84801
H	6.35337	2.11968	2.48231
Si	3.72155	-2.92290	-1.02579
Si	-0.88739	-2.13034	-1.65945
Mg	3.09502	-0.00120	0.04722
N	4.35615	-1.43914	-0.30363
N	-2.22723	-1.79611	-0.55570
C	5.74786	-1.25588	-0.01115
C	6.61981	-0.67181	-0.99038
C	7.97291	-0.45190	-0.66892
H	8.63124	0.00200	-1.41866
C	8.49569	-0.79476	0.58339
H	9.55197	-0.61711	0.81181
C	7.64774	-1.36240	1.54049
H	8.04952	-1.62425	2.52662
C	6.28621	-1.60085	1.27126
C	6.10345	-0.23099	-2.36172
H	5.10515	-0.68609	-2.48611
C	6.99213	-0.71464	-3.52754
H	7.13848	-1.80697	-3.49830
H	6.52968	-0.45499	-4.49637
H	7.99003	-0.24195	-3.50803
C	5.92800	1.30459	-2.42384
H	6.89242	1.81858	-2.26504
H	5.53464	1.61940	-3.40681
H	5.23372	1.68188	-1.65084
C	5.40446	-2.17339	2.38098
H	4.42623	-2.40230	1.92444
C	5.96798	-3.48014	2.97937
H	6.92564	-3.31031	3.50272
H	5.26305	-3.90755	3.71423
H	6.14916	-4.23694	2.19768
C	5.17022	-1.12796	3.49658
H	4.73292	-0.19633	3.09377
H	4.48939	-1.52013	4.27361
H	6.11976	-0.85174	3.98853
C	4.83778	-3.62180	-2.40245
H	5.87552	-3.72769	-2.04295
H	4.48813	-4.62075	-2.71599
H	4.85474	-2.97044	-3.29149
C	3.41821	-4.34291	0.21684
H	2.73599	-4.04935	1.03313
H	2.97368	-5.21437	-0.29646
H	4.36294	-4.67665	0.67770
C	2.01796	-2.46334	-1.79287
H	1.68097	-3.40084	-2.27559
H	2.17904	-1.75656	-2.63045
C	0.88203	-1.94565	-0.86022
H	0.88142	-2.47698	0.11072
H	0.98138	-0.85479	-0.63175
C	-0.93203	-0.86070	-3.08288
H	-0.95481	0.17497	-2.69645
H	-0.03892	-0.94790	-3.72559
H	-1.82166	-1.00034	-3.71955
C	-1.00008	-3.90841	-2.32984
H	-1.99360	-4.09143	-2.76762
H	-0.24971	-4.09446	-3.11560
H	-0.85041	-4.65055	-1.52781
C	-3.03918	-2.87106	-0.04192
C	-2.77202	-3.44369	1.24478
C	-3.61192	-4.45772	1.74534
H	-3.39629	-4.89078	2.72949
C	-4.71143	-4.92277	1.01725
H	-5.35561	-5.70928	1.42415
C	-4.97794	-4.36729	-0.23988
H	-5.83971	-4.72842	-0.81254
C	-4.16485	-3.35862	-0.79100
C	-1.59033	-2.98416	2.09829
H	-1.10909	-2.15375	1.54962
C	-2.04213	-2.45102	3.47656
H	-1.18132	-2.05996	4.04790
H	-2.78622	-1.64086	3.37649
H	-2.50896	-3.24519	4.08536
C	-0.54236	-4.10665	2.27146
H	-0.96915	-4.97225	2.80851
H	-0.17679	-4.47204	1.29682
H	0.32596	-3.74908	2.85402
C	-4.52703	-2.78736	-2.16456
H	-3.64920	-2.21592	-2.51482
C	-4.83632	-3.87727	-3.21453
H	-4.96698	-3.42144	-4.21151
H	-4.02985	-4.62681	-3.28286
H	-5.77029	-4.41785	-2.98044
C	-5.71621	-1.80371	-2.07092
H	-6.62221	-2.31717	-1.70430
H	-5.52667	-0.96338	-1.37847
H	-5.94967	-1.36891	-3.05872

10

SCF (BP86) Energy = -3619.86336997
Enthalpy 0K = -3618.068663
Enthalpy 298K = -3617.948950
Free Energy 298K = -3618.228688
Lowest Frequency = 7.6685 cm^{-1}

Second Frequency = 11.1865 cm^{-1}
SCF (BP86-D3BJ) Energy = -3620.50432189
SCF (C6H6) Energy = -3619.88691699
SCF (BS2) Energy = -5485.90799346

```
Si -2.07291  2.39150 -0.65770
Si  2.34087  2.07714  0.76259
Si  2.07281 -2.39153 -0.65760
Si -2.34099 -2.07711  0.76265
Mg  4.57763 -0.22715  0.08335
Mg -4.57768  0.22723  0.08321
Na -7.22394 -2.63862 -1.84952
N  -3.66244  2.04526  0.06176
N   3.66239 -2.04517  0.06173
N  -3.89238 -1.74093 -0.06746
N   6.67617 -0.24749  0.20746
N   3.89226  1.74094 -0.06750
N  -6.67619  0.24755  0.20723
C  -5.36539  3.87183 -0.15044
C  -4.37804  3.16148  0.61920
C   4.37797 -3.16144  0.61911
C  -7.03512  0.02890 -2.18543
H  -6.06336  0.50774 -2.35452
C   4.52874  2.80037 -0.74313
C  -4.12297  3.62468  1.95647
C  -7.51279 -0.07931 -0.83275
C  -3.06239  2.98398  2.85159
H  -2.61577  2.15767  2.27329
C  -7.21314  0.53220  1.48572
C  -4.33659 -3.02810 -2.16531
C   4.33680  3.02775 -2.16551
C   5.69957 -3.50057 -1.59660
H   5.21966 -2.52225 -1.78785
C  -5.79311  5.40368  1.71912
H  -6.33317  6.25996  2.13758
C   5.40754  3.73580 -0.05623
C   5.36533 -3.87176 -0.15055
C  -4.52882 -2.80044 -0.74302
C  -5.69947  3.50082 -1.59657
H  -5.21960  2.52249 -1.78786
C  -3.34095 -2.19954 -2.97487
H  -2.88820 -1.48959 -2.26213
C   7.21316 -0.53222  1.48593
C  -1.77105  4.26785 -0.91326
H  -2.01182  4.59648 -1.93765
H  -0.70897  4.50974 -0.73236
H  -2.37765  4.86906 -0.21627
C   4.12286 -3.62473  1.95635
C  -0.56378  1.81617  0.40547
H  -0.53241  2.50446  1.27272
H  -0.74284  0.81580  0.83454
C   7.51274  0.07937 -0.83254
C  -8.83713 -0.63300 -0.68781
H  -9.26260 -0.72653  0.31504
C   1.77098 -4.26788 -0.91291
H   2.01348 -4.59708 -1.93670
H   0.70851 -4.50938 -0.73380
H   2.37618 -4.86894 -0.21457
C  -6.04299  4.97031  0.41352
H  -6.78278  5.50516 -0.19408
C   2.29121  0.93270  2.28686
H   2.56136 -0.11131  2.04681
H   1.28062  0.90750  2.72934
H   2.98850  1.29901  3.06054
C   3.06239 -2.98396  2.85156
H   2.61572 -2.15767  2.27326
C  -4.83551  4.72283  2.47567
H  -4.62296  5.05520  3.49931
C  -5.40789 -3.73569 -0.05615
C  -7.79919 -0.38249 -3.28723
H  -7.38981 -0.24996 -4.29505
C  -6.52415  0.08475  2.64093
H  -5.63359 -0.54744  2.51997
C  -1.97037  1.53343 -2.36496
H  -2.21138  0.45832 -2.30214
H  -0.96127  1.61880 -2.80393
H  -2.68779  2.00052 -3.06183
C   0.56369 -1.81614  0.40557
H   0.53231 -2.50436  1.27287
H   0.74283 -0.81575  0.83457
C  -8.37749  1.32035  1.68079
H  -8.90648  1.71668  0.80839
C   5.71673  3.62098  1.43764
H   5.03781  2.84855  1.83878
C   6.04284 -4.97034  0.41332
H   6.78263 -5.50516 -0.19429
C   4.83530 -4.72298  2.47546
H   4.62271 -5.05544  3.49906
C   7.03500 -0.02860 -2.18521
H   6.06313 -0.50720 -2.35432
C   6.52423 -0.08476  2.64117
H   5.63370  0.54748  2.52024
C   1.97013 -1.53364 -2.36496
H   2.21114 -0.45852 -2.30222
H   0.96101 -1.61905 -2.80388
H   2.68754 -2.00076 -3.06182
C   6.03674  4.78483 -0.76612
H   6.67180  5.48745 -0.21320
C  -0.79279 -1.82399 -0.35238
H  -0.82296 -2.65650 -1.08408
H  -0.89105 -0.90317 -0.95517
C   7.21893 -3.36970 -1.83696
H   7.68396 -2.68592 -1.10996
H   7.42335 -2.98541 -2.85197
H   7.72559 -4.34756 -1.75194
C  -9.08325 -0.94718 -3.11493
H  -9.68745 -1.24757 -3.97553
C   3.34125  2.19912 -2.97509
H   2.88851  1.48915 -2.26235
C  -6.03702 -4.78476 -0.76604
H  -6.67229 -5.48723 -0.21316
C   5.79286 -5.40383  1.71887
H   6.33285 -6.26020  2.13725
C   8.37747 -1.32044  1.68094
H   8.90641 -1.71677  0.80851
C   5.01358  4.07397 -2.83039
H   4.83636  4.21884 -3.90298
C  -9.58788 -1.04804 -1.79747
H  -10.58932 -1.46232 -1.63065
C   0.79270  1.82406 -0.35248
H   0.82287  2.65663 -1.08411
H   0.89095  0.90328 -0.95534
C  -8.83526  1.62071  2.96974
H  -9.73147  2.23992  3.08757
C  -1.93463  3.98183  3.20171
H  -1.48071  4.41603  2.29458
H  -1.13736  3.48635  3.78506
H  -2.31832  4.82084  3.80959
C  -7.21880  3.37008 -1.83718
H  -7.68399  2.68623 -1.11034
H  -7.42308  2.98594 -2.85228
H  -7.72541  4.34796 -1.75212
C   7.17083  3.14558  1.65908
C  -5.01327 -4.07440 -2.83017
H  -4.83581 -4.21947 -3.90270
C   7.79915  0.38266 -3.28699
```

H 7.38973 0.25026 -4.29481
C 5.86183 4.96442 -2.14980
H 6.34328 5.79649 -2.67488
C 2.19554 3.88085 1.37329
H 2.76904 4.05454 2.29730
H 1.13827 4.10874 1.59312
H 2.53821 4.60153 0.61134
C 1.93465 -3.98173 3.20195
H 1.48054 -4.41599 2.29494
H 1.13750 -3.48615 3.78539
H 2.31839 -4.82070 3.80985
C -2.29137 -0.93259 2.28686
H -2.56136 0.11144 2.04665
H -1.28083 -0.90745 2.72946
H -2.98880 -1.29872 3.06050
C 8.83526 -1.62086 2.96987
H 9.73143 -2.24012 3.08765
C -5.86178 -4.96463 -2.14963
H -6.34322 -5.79672 -2.67468
C -6.98379 0.39374 3.92992
H -6.43049 0.02548 4.80059
C 5.10969 -4.51459 -2.60426
H 5.53320 -5.52117 -2.43582
H 5.34663 -4.22024 -3.64335
H 4.01623 -4.59517 -2.51001
C -5.10932 4.51491 -2.60401
H -5.53283 5.52148 -2.43556
H -5.34604 4.22067 -3.64318
H -4.01589 4.59542 -2.50949
C 8.83718 0.63284 -0.68760
H 9.26268 0.72625 0.31526
C -5.71731 -3.62080 1.43768
H -5.03857 -2.84819 1.83881
C -2.21633 -3.08767 -3.55591
H -1.73779 -3.70423 -2.77797
H -1.43664 -2.46245 -4.02449
H -2.60387 -3.77221 -4.33213
C -3.66430 2.38855 4.14302
H -4.10252 3.17611 4.78211
H -2.88714 1.87278 4.73574
H -4.46676 1.66827 3.91558
C 8.14755 -1.15859 4.10539
H 8.50785 -1.39980 5.11016
C -7.17153 -3.14579 1.65916
H -7.35982 -2.17930 1.16463
H -7.38558 -3.00672 2.73266
H -7.89062 -3.89339 1.27244
C -8.14748 1.15846 4.10524
H -8.50777 1.39962 5.11002
C 6.98390 -0.39379 3.93013
H 6.43065 -0.02552 4.80084
C -4.00545 -1.39145 -4.11019
H -4.47151 -2.05738 -4.85986
H -3.25602 -0.77400 -4.63442
H -4.78761 -0.71571 -3.72751
C 9.08335 0.94703 -3.11471
H 9.68762 1.24728 -3.97531
C -2.19565 -3.88080 1.37338
H -2.76912 -4.05445 2.29742
H -1.13838 -4.10869 1.59318
H -2.53836 -4.60150 0.61146
C 9.58802 1.04776 -1.79724
H 10.58956 1.46180 -1.63042
C 4.00575 1.39109 -4.11044
H 3.25630 0.77367 -4.63470
H 4.78791 0.71534 -3.72781
H 4.47179 2.05705 -4.86009
C 3.66452 -2.38844 4.14285
H 4.10289 -3.17597 4.78189
H 2.88746 -1.87266 4.73569
H 4.46692 -1.66816 3.91523
C 2.21660 3.08724 -3.55610
H 2.60412 3.77179 -4.33231
H 1.73805 3.70378 -2.77814
H 1.43691 2.46201 -4.02468
C 5.48751 4.94098 2.20854
H 5.59225 4.76787 3.29380
H 4.48860 5.36354 2.02226
H 6.23419 5.70734 1.93193
C -5.48776 -4.94071 2.20865
H -6.23414 -5.70732 1.93195
H -5.59269 -4.76761 3.29389
H -4.48869 -5.36295 2.02251
H 7.38501 3.00674 2.73259
H 7.35872 2.17889 1.16480
H 7.89010 3.89288 1.27207
Na 7.22513 2.63927 -1.84916

A

SCF (BP86) Energy = -2816.15663414
Enthalpy 0K = -2814.613065
Enthalpy 298K = -2814.508048
Free Energy 298K = -2814.766222
Lowest Frequency = 6.5778 cm^{-1}
Second Frequency = 6.8836 cm^{-1}
SCF (BP86-D3BJ) Energy = -2816.65407271
SCF (C6H6) Energy = -2816.17597893
SCF (BS2) Energy = -4682.02144974

Si 6.98925 -1.15356 -1.61250
Si 5.63135 2.80077 -1.56933
Si -6.67562 -2.72363 0.64638
Si -7.31046 1.44510 0.69240
Mg 4.52250 0.22531 0.05946
Mg -4.44493 -0.23123 -0.01750
Na -1.96293 -1.48721 -1.60830
Na -1.90258 1.92874 -0.01352
N 5.93345 -1.13018 -0.18686
N 4.24099 1.97479 -0.82127
N -5.54610 1.38967 0.60165
N -5.21932 -2.14075 -0.16715
C 6.07238 -2.08151 0.86375
C -4.37405 -2.93375 -0.96024
C 2.50170 3.46964 0.15461
C 6.86646 -1.77362 2.02228
C 2.95070 2.56106 -0.86751
C 5.37510 -3.33712 0.83460
C -4.70237 2.48129 0.86535
C -3.30757 -3.73170 -0.39421
C -4.45399 -2.87010 -2.40460
C -4.01063 2.58017 2.13291
C 5.44951 -4.20555 1.94198
H 4.90731 -5.15883 1.90858
C 4.53380 -3.74809 -0.37334
H 4.60431 -2.91859 -1.09801
C -8.03058 -0.16811 1.43936
H -7.52393 -0.37547 2.40160
H -9.06087 0.13248 1.72365
C 2.03841 2.23612 -1.93337
C 6.90148 -2.67046 3.10680
H 7.50002 -2.41780 3.99029
C 1.20136 4.00761 0.09349
H 0.87872 4.71384 0.86854
C -8.10049 -1.44467 0.55563
H -8.27893 -1.18431 -0.50538
H -8.98761 -2.04322 0.85159

C	7.63239	0.59959	-2.06288
H	8.14245	1.02571	-1.17725
H	8.45071	0.38590	-2.78228
C	-3.18247	-3.91404	1.11715
H	-3.91838	-3.22745	1.56867
C	-4.38852	3.47665	-0.13659
C	6.66828	1.64003	-2.69577
H	5.99154	1.15629	-3.42678
H	7.26208	2.35908	-3.29893
C	-2.36424	-4.35931	-1.23611
H	-1.56449	-4.95885	-0.78389
C	6.19728	-3.88152	3.08101
H	6.24007	-4.56835	3.93303
C	-3.04278	3.58448	2.34095
H	-2.53199	3.63730	3.31061
C	-5.60170	-2.13215	-3.09388
H	-6.24119	-1.74423	-2.28107
C	0.74999	2.80159	-1.95374
H	0.07534	2.56582	-2.78564
C	-2.73937	4.53616	1.35357
H	-2.00079	5.32274	1.54146
C	-3.47775	-3.50431	-3.20149
H	-3.55449	-3.43160	-4.29347
C	2.45309	1.27259	-3.04547
H	3.55153	1.17936	-2.97885
C	3.42031	3.88604	1.30356
H	4.37065	3.34536	1.15090
C	-4.33315	1.62025	3.27831
H	-5.12932	0.95520	2.89870
C	-3.42673	4.47480	0.12993
H	-3.21735	5.22824	-0.63997
C	-2.42394	-4.24211	-2.63563
H	-1.68306	-4.73890	-3.27132
C	-8.18391	1.65638	-1.00286
H	-8.06719	2.67495	-1.40823
H	-9.26674	1.46117	-0.89898
H	-7.79071	0.94910	-1.75399
C	0.32206	3.69040	-0.95323
H	-0.65665	4.18260	-1.02049
C	6.09858	-1.81333	-3.17568
H	5.89004	-2.89366	-3.09914
H	6.71759	-1.65427	-4.07689
H	5.13546	-1.29831	-3.34069
C	8.51662	-2.24684	-1.28523
H	9.16915	-1.80522	-0.51318
H	9.11394	-2.36651	-2.20552
H	8.22371	-3.25143	-0.93692
C	-6.43429	-3.08605	2.51556
H	-5.83657	-3.99578	2.69123
H	-7.41411	-3.23142	3.00553
H	-5.92923	-2.24838	3.02806
C	7.65171	-0.46408	2.10557
H	7.67251	-0.04943	1.08201
C	-7.31037	-4.32522	-0.18192
H	-7.72508	-4.11644	-1.18285
H	-8.10734	-4.79274	0.42221
H	-6.50090	-5.06497	-0.30465
C	5.08340	-5.02403	-1.04884
H	6.13713	-4.89692	-1.34881
H	4.49788	-5.27399	-1.95187
H	5.03533	-5.89351	-0.36899
C	-5.09285	3.48113	-1.49254
H	-5.70623	2.56484	-1.52181
C	6.83668	3.49769	-0.25568
H	6.38986	4.33751	0.30229
H	7.76496	3.86407	-0.72949
H	7.12468	2.72426	0.47843
C	3.04220	-3.92654	-0.01109

H	2.89657	-4.72882	0.73433
H	2.45015	-4.19165	-0.90564
H	2.62136	-2.99728	0.41231
C	-3.54371	-5.35868	1.53228
H	-4.55347	-5.63634	1.18687
H	-3.51468	-5.47008	2.63102
H	-2.83266	-6.08699	1.10162
C	2.85935	3.47364	2.68268
H	1.88399	3.95136	2.88630
H	3.55009	3.77172	3.49130
H	2.71756	2.38069	2.74599
C	-1.79027	-3.52820	1.66145
H	-0.99146	-4.17422	1.25330
H	-1.76502	-3.62830	2.76126
H	-1.54764	-2.47903	1.40803
C	5.02231	4.23716	-2.66567
H	5.87776	4.82726	-3.03713
H	4.35463	4.91766	-2.11144
H	4.46386	3.86492	-3.54121
C	-4.10949	3.42274	-2.68203
H	-3.44462	4.30547	-2.71411
H	-4.66021	3.39303	-3.63891
H	-3.47758	2.51600	-2.63247
C	-7.87548	2.88439	1.81678
H	-7.63997	2.67937	2.87507
H	-8.96546	3.04109	1.73841
H	-7.37716	3.82966	1.54145
C	-3.12546	0.74084	3.67037
H	-2.76409	0.15150	2.80587
H	-3.39792	0.03516	4.47551
H	-2.28159	1.35481	4.03685
C	-6.44816	-3.09063	-3.96171
H	-5.86496	-3.49192	-4.81049
H	-7.32301	-2.56254	-4.38039
H	-6.81332	-3.94846	-3.37295
C	9.10918	-0.66131	2.57245
H	9.16637	-1.00757	3.61982
H	9.66571	0.29053	2.51258
H	9.63163	-1.40463	1.94748
C	-5.11555	-0.92890	-3.93132
H	-4.55844	-0.20414	-3.30800
H	-5.96985	-0.39865	-4.38853
H	-4.44579	-1.24894	-4.75111
C	6.93485	0.56845	3.00638
H	5.91560	0.79754	2.63922
H	7.49119	1.52190	3.04642
H	6.83104	0.19142	4.03977
C	1.85828	-0.13578	-2.81281
H	2.17390	-0.56010	-1.84120
H	2.17300	-0.84032	-3.60325
H	0.75142	-0.09546	-2.80633
C	3.72226	5.40126	1.27516
H	4.15474	5.70777	0.30813
H	4.43785	5.67224	2.07170
H	2.80639	5.99875	1.43344
C	-6.03228	4.70139	-1.62741
H	-6.76300	4.74094	-0.80234
H	-6.59107	4.66212	-2.57945
H	-5.46222	5.64819	-1.61238
C	-4.87852	2.37229	4.51311
H	-4.11387	3.03728	4.95435
H	-5.18920	1.65803	5.29609
H	-5.74966	2.99499	4.25046
C	2.09734	1.77749	-4.45924
H	1.00568	1.81356	-4.62600
H	2.52140	1.10449	-5.22482
H	2.49535	2.79067	-4.63593
O	3.08220	-0.27653	1.43314

C 1.63653 -0.00681 1.26195
H 1.24635 -0.70629 0.49948
H 1.53540 1.02839 0.89790
C 3.31121 -0.97011 2.72483
H 3.77440 -0.24161 3.41341
H 4.00599 -1.80231 2.53656
C 1.91896 -1.39262 3.19051
H 1.64208 -2.36470 2.74749
H 1.86582 -1.48642 4.28669
C 1.02201 -0.26839 2.63587
H 1.09078 0.63310 3.26953
H -0.03868 -0.55602 2.55675

TS(6-A)
SCF (BP86) Energy = -2816.12467484
Enthalpy 0K = -2814.577285
Enthalpy 298K = -2814.475564
Free Energy 298K = -2814.712583
Lowest Frequency = -42.4725 cm^{-1}
Second Frequency = 18.7826 cm^{-1}
SCF (BP86-D3BJ) Energy = -2816.66911984
SCF (C6H6) Energy = -2816.14011946
grep: mg2na2_thf_to_mg_ts_opt1_BB*.out: No such file or directory
SCF (BS2) Energy =

Si 4.15054 -0.48396 -2.33491
Si 4.87089 1.34602 1.04014
Si -5.10379 -1.21874 1.28168
Si -4.82446 1.48439 -1.60478
Mg 1.93785 -0.08087 0.19232
Mg -2.16097 0.05451 0.03541
Na -0.16495 -3.16536 -0.32411
Na -0.07981 3.34724 0.29490
N 2.93527 -1.27883 -1.29512
N 3.11002 1.57478 0.85016
N -3.24167 1.65945 -0.79904
N -3.46037 -1.51041 0.65652
C 2.40315 -2.51607 -1.71734
C -2.89906 -2.79126 0.80729
C 1.78731 3.00372 2.40258
C 2.92926 -3.78224 -1.22891
C 2.56102 2.83205 1.18457
C 1.29723 -2.59814 -2.65823
C -2.68019 2.95123 -0.83944
C -2.22646 -3.18710 2.02843
C -2.94988 -3.77128 -0.26224
C -2.88132 3.89217 0.24386
C 0.73592 -3.84992 -2.99843
H -0.07270 -3.88294 -3.73736
C 0.76330 -1.36729 -3.39526
H 1.16634 -0.48344 -2.87075
C -6.25026 0.90016 -0.46549
H -6.26509 1.55770 0.42548
H -7.16387 1.17325 -1.03564
C 2.71742 4.01950 0.36046
C 2.30124 -4.99732 -1.57725
H 2.70364 -5.93599 -1.18311
C 1.19716 4.24509 2.72187
H 0.63468 4.33696 3.65822
C -6.32949 -0.58131 -0.04525
H -6.25649 -1.24120 -0.93088
H -7.33000 -0.78994 0.38987
C 5.78312 -0.04173 -1.41530
H 6.05495 -0.91368 -0.79048
H 6.54385 -0.01976 -2.22394
C -2.28374 -2.30242 3.27351
H -2.74551 -1.35353 2.95172
C -1.87871 3.40438 -1.95911
C 5.89331 1.25818 -0.58503
H 5.68712 2.13607 -1.22282
H 6.94534 1.38814 -0.25194
C -1.57543 -4.43562 2.11171
H -1.08263 -4.71517 3.05142
C 1.20232 -5.04948 -2.44636
H 0.74543 -6.00735 -2.71558
C -2.28509 5.17076 0.20418
H -2.46114 5.86411 1.03556
C -3.79797 -3.51877 -1.50904
H -3.88955 -2.42095 -1.59950
C 2.11344 5.24035 0.73559
H 2.26107 6.11637 0.09337
C -1.50038 5.58763 -0.88289
H -1.05626 6.58789 -0.90242
C -2.28189 -5.00908 -0.12541
H -2.33445 -5.73557 -0.94410
C 3.55661 4.02867 -0.91697
H 3.83837 2.98291 -1.11234
C 1.70015 1.88998 3.44053
H 2.07897 0.98539 2.93814
C -3.78531 3.54525 1.42473
H -4.19403 2.54527 1.19973
C -1.31293 4.69822 -1.95293
H -0.72241 5.02233 -2.81814
C -1.57501 -5.34613 1.04208
H -1.07230 -6.31517 1.12972
C -4.73513 0.26278 -3.07508
H -4.14678 0.67891 -3.91013
H -5.74636 0.03231 -3.45585
H -4.26612 -0.69046 -2.77696
C 1.34389 5.37132 1.89930
H 0.88688 6.32900 2.16787
C 3.47603 1.11979 -3.13111
H 2.82870 0.88944 -3.99338
H 4.30390 1.75645 -3.49325
H 2.88484 1.71216 -2.41590
C 4.70482 -1.58811 -3.81907
H 5.76998 -1.86567 -3.74396
H 4.59020 -1.02033 -4.75927
H 4.12462 -2.52008 -3.91463
C -5.11501 0.06287 2.70609
H -4.71278 -0.37080 3.63739
H -6.14742 0.39900 2.91294
H -4.51620 0.95598 2.46453
C 4.21875 -3.85264 -0.40044
H 4.25911 -2.93464 0.21634
C -5.91342 -2.81897 1.98765
H -6.81068 -3.09609 1.40782
H -6.24376 -2.64939 3.02732
H -5.23449 -3.68706 1.98633
C 1.26127 -1.34785 -4.86146
H 2.35803 -1.38524 -4.92788
H 0.91545 -0.43376 -5.37688
H 0.86255 -2.21509 -5.41862
C -1.65611 2.52188 -3.18776
H -2.11444 1.54637 -2.94833
C 5.26268 -0.31105 1.91572
H 5.18354 -0.20370 3.01076
H 6.29935 -0.61769 1.68834
H 4.59040 -1.12376 1.59606
C -0.77392 -1.26903 -3.40092
H -1.24349 -2.09457 -3.96250
H -1.09890 -0.33061 -3.87845
H -1.18589 -1.26911 -2.37268
C -3.17102 -2.93839 4.36983
H -4.18561 -3.15820 4.00545

```
H  -3.25659 -2.25977  5.23745
H  -2.73200 -3.88643  4.73117
C   0.26940  1.61615  3.93990
H  -0.19244  2.50929  4.39684
H   0.27154  0.82966  4.71508
H  -0.37996  1.26926  3.11606
C  -0.90099 -1.97273  3.86048
H  -0.40064 -2.88024  4.24721
H  -1.00792 -1.27757  4.71206
H  -0.24439 -1.50185  3.10986
C   5.63419  2.72322  2.14068
H   6.67486  2.93617  1.83994
H   5.66009  2.39913  3.19553
H   5.06916  3.66815  2.10044
C  -0.15952  2.29401 -3.47461
H   0.35925  3.23844 -3.71601
H  -0.02283  1.62419 -4.34155
H   0.34777  1.82751 -2.61115
C  -5.45806  3.17119 -2.25769
H  -5.90379  3.76254 -1.43926
H  -6.24919  3.00355 -3.00988
H  -4.67394  3.79095 -2.71911
C  -3.00444  3.45745  2.75285
H  -2.20373  2.69841  2.70254
H  -3.67393  3.17769  3.58548
H  -2.53662  4.42530  3.01148
C  -5.22282 -4.09470 -1.32463
H  -5.18161 -5.19268 -1.20494
H  -5.84633 -3.87224 -2.20886
H  -5.72368 -3.67942 -0.43903
C   5.47175 -3.84543 -1.30930
H   5.45734 -4.71516 -1.99029
H   6.38880 -3.90768 -0.69638
H   5.53346 -2.93813 -1.92145
C  -3.20236 -4.08799 -2.81156
H  -2.15198 -3.79038 -2.95498
H  -3.77950 -3.72707 -3.68034
H  -3.24503 -5.19221 -2.83475
C   4.32331 -5.09905  0.50762
H   3.41500 -5.28796  1.10269
H   5.17192 -4.98639  1.20375
H   4.52279 -6.00963 -0.08539
C   2.77202  4.54685 -2.14157
H   1.83668  3.98478 -2.29713
H   3.37786  4.44163 -3.05819
H   2.51083  5.61558 -2.03916
C   2.63452  2.20465  4.63309
H   3.66916  2.38201  4.29808
H   2.64189  1.37093  5.35837
H   2.29743  3.10991  5.16953
C  -2.34334  3.09296 -4.45032
H  -3.42916  3.21349 -4.31292
H  -2.18292  2.42013 -5.31165
H  -1.92667  4.08056 -4.71924
C  -4.96766  4.53016  1.56082
H  -4.62355  5.54796  1.81987
H  -5.65331  4.19887  2.36036
H  -5.54380  4.60180  0.62377
C   4.85238  4.85805 -0.75365
H   4.61952  5.91810 -0.54530
H   5.44979  4.82414 -1.68213
H   5.48204  4.48559  0.06861
O   2.17662 -1.66604  2.40318
C   2.49073 -1.37850  3.80582
H   1.63811 -0.85341  4.26534
H   3.36616 -0.71226  3.80835
C   2.06206 -3.10141  2.25578
H   2.33758 -3.34309  1.21766
H   1.01723 -3.42317  2.44673
C   3.00819 -3.68595  3.30826
H   2.78704 -4.73876  3.54827
H   4.05057 -3.62081  2.95503
C   2.77342 -2.73070  4.49351
H   3.63724 -2.67502  5.17532
H   1.89938 -3.05651  5.08278
```

B

SCF (BP86) Energy = -2816.16009457
Enthalpy 0K = -2814.615754
Enthalpy 298K = -2814.511844
Free Energy 298K = -2814.757898
Lowest Frequency = 8.1388 cm^{-1}
Second Frequency = 17.6123 cm^{-1}
SCF (BP86-D3BJ) Energy = -2816.6978609
SCF (C6H6) Energy = -2816.17694050
SCF (BS2) Energy = -4682.01291891

```
Si -1.03160  4.07948  2.03062
Si  0.50919  4.67208 -1.50457
Si  0.18195 -4.47457 -1.51629
Si  2.66297 -4.15977  1.49231
Mg  0.28731  1.64068  0.09816
Mg  0.88464 -1.57391  0.04621
Na -4.16537 -0.68809  0.04915
Na  3.87273  0.69624 -0.36882
N  -1.27747  2.49390  1.25353
N   1.31251  3.17810 -0.97071
N   2.65253 -2.52472  0.78996
N  -0.48252 -3.01995 -0.71562
C  -2.43644  1.80485  1.65134
C  -1.86861 -2.87952 -0.90554
C   2.86598  2.14609 -2.62652
C  -3.73605  2.09573  1.07578
C   2.61800  2.94365 -1.43831
C  -2.39950  0.79577  2.69137
C   3.84264 -1.78298  0.91541
C  -2.41277 -2.16558 -2.04589
C  -2.83083 -3.50532 -0.01757
C   4.90831 -1.86808 -0.06608
C  -3.58380  0.15995  3.11379
H  -3.52906 -0.57125  3.92931
C  -1.10651  0.47109  3.43857
H  -0.29404  0.97758  2.88823
C   2.35568 -5.56759  0.22738
H   3.07452 -5.43741 -0.60480
H   2.69071 -6.48749  0.75255
C   3.78643  3.46076 -0.74997
C  -4.89730  1.45575  1.56042
H  -5.87003  1.73216  1.13633
C   4.18712  1.86439 -3.03650
H   4.34485  1.26713 -3.94215
C   0.92914 -5.77825 -0.32099
H   0.21893 -5.90896  0.51678
H   0.88474 -6.73193 -0.88850
C  -1.12581  5.59129  0.84893
H  -2.06063  5.50145  0.26321
H  -1.29490  6.45980  1.52063
C  -1.50921 -1.42909 -3.03489
H  -0.47984 -1.52922 -2.64733
C   4.06068 -0.87817  2.03008
C   0.05956  5.89316 -0.09228
H   0.98159  6.05778  0.49878
H  -0.11805  6.85862 -0.61285
C  -3.79652 -2.20797 -2.32402
H  -4.16726 -1.71996 -3.23448
C  -4.83891  0.48111  2.57101
```

H	-5.75147	0.01934	2.96512
C	6.06246	-1.06294	0.05335
H	6.85059	-1.15271	-0.70419
C	-2.39272	-4.23749	1.24976
H	-1.31351	-4.03185	1.36055
C	5.08604	3.14579	-1.20342
H	5.94838	3.55404	-0.66237
C	6.23785	-0.16992	1.12161
H	7.14317	0.44069	1.19990
C	-4.20448	-3.51989	-0.34316
H	-4.89635	-4.06570	0.31067
C	3.65533	4.38615	0.45892
H	2.57316	4.50317	0.63603
C	1.71632	1.66387	-3.51164
H	0.79134	1.82796	-2.93026
C	4.83765	-2.84547	-1.23859
H	3.88721	-3.39211	-1.12124
C	5.23524	-0.09860	2.10159
H	5.37586	0.56938	2.95945
C	-4.69864	-2.90709	-1.50547
H	-5.75909	-2.97616	-1.76769
C	1.34975	-4.32323	2.87428
H	1.63395	-3.72782	3.75865
H	1.24296	-5.37538	3.19470
H	0.35922	-3.97260	2.53990
C	5.30400	2.34430	-2.33457
H	6.31972	2.11638	-2.67367
C	0.65985	4.10938	2.91814
H	0.65111	3.43585	3.79193
H	0.91004	5.12573	3.27210
H	1.47024	3.78052	2.24785
C	-2.35619	4.43602	3.36895
H	-3.33796	4.68613	2.93490
H	-2.03189	5.30152	3.97384
H	-2.51131	3.58543	4.05162
C	1.51363	-3.97962	-2.79433
H	1.05157	-3.48819	-3.66727
H	2.07094	-4.86266	-3.15566
H	2.24391	-3.27833	-2.36042
C	-3.89842	3.12809	-0.04097
H	-2.89194	3.53939	-0.22237
C	-1.16465	-5.43195	-2.48849
H	-1.91231	-5.90161	-1.82851
H	-0.67955	-6.23670	-3.06938
H	-1.71506	-4.79045	-3.19524
C	-1.13433	1.03178	4.88052
H	-1.26540	2.12525	4.89364
H	-0.19191	0.79715	5.40725
H	-1.96045	0.58666	5.46437
C	3.06961	-0.79996	3.19057
H	2.17356	-1.35573	2.86243
C	-1.08374	4.25240	-2.47865
H	-0.83170	3.83553	-3.46886
H	-1.70869	5.14984	-2.63728
H	-1.69389	3.50514	-1.94560
C	-0.79863	-1.03682	3.47893
H	-1.55025	-1.59989	4.06008
H	0.18075	-1.22022	3.95197
H	-0.76804	-1.46728	2.46131
C	-1.55800	-2.04737	-4.45103
H	-1.27430	-3.11175	-4.44301
H	-0.86530	-1.51725	-5.12853
H	-2.56929	-1.97341	-4.89082
C	1.80877	0.16794	-3.86678
H	2.72589	-0.07231	-4.43371
H	0.95450	-0.13356	-4.49734
H	1.78901	-0.45892	-2.95720
C	-1.85293	0.07211	-3.10569
H	-2.89347	0.23866	-3.44081
H	-1.19225	0.59905	-3.81507
H	-1.72172	0.55138	-2.11792
C	1.62468	5.71231	-2.67183
H	2.19852	6.46832	-2.10944
H	0.99637	6.25677	-3.39897
H	2.34707	5.10227	-3.23754
C	2.64997	0.64201	3.53225
H	3.50300	1.25498	3.87457
H	1.90330	0.64613	4.34508
H	2.19413	1.14204	2.65868
C	4.35982	-4.59847	2.27279
H	5.04665	-5.02451	1.52198
H	4.21834	-5.36386	3.05642
H	4.86681	-3.73397	2.72989
C	4.81334	-2.12158	-2.60237
H	3.96333	-1.42180	-2.68176
H	4.71895	-2.84727	-3.42925
H	5.74183	-1.54509	-2.76869
C	-2.58419	-5.76843	1.14355
H	-3.65233	-6.02567	1.02144
H	-2.22391	-6.26645	2.06117
H	-2.03735	-6.19652	0.28962
C	-4.84053	4.29215	0.33766
H	-5.87256	3.93821	0.51564
H	-4.88174	5.03461	-0.47895
H	-4.50480	4.81103	1.24912
C	-3.13493	-3.72263	2.50262
H	-3.05797	-2.62780	2.60448
H	-2.71297	-4.17689	3.41640
H	-4.20832	-3.98708	2.47370
C	-4.39178	2.47424	-1.35084
H	-3.69568	1.69252	-1.70307
H	-4.47285	3.22143	-2.15995
H	-5.38929	2.01806	-1.21159
C	4.29048	3.78735	1.73237
H	3.84908	2.80969	1.99161
H	4.13673	4.45721	2.59659
H	5.37993	3.64445	1.61172
C	1.62741	2.50477	-4.80805
H	1.50510	3.57798	-4.59293
H	0.76913	2.17942	-5.42333
H	2.54084	2.38682	-5.41895
C	3.63266	-1.49163	4.45538
H	3.88978	-2.54595	4.26773
H	2.89189	-1.46176	5.27462
H	4.54591	-0.98154	4.81201
C	5.99417	-3.87037	-1.21162
H	6.97233	-3.38088	-1.37004
H	5.86446	-4.61805	-2.01389
H	6.04210	-4.40459	-0.24938
C	4.25615	5.78303	0.18156
H	5.35030	5.72868	0.03517
H	4.06857	6.45991	1.03377
H	3.82221	6.23953	-0.72204
O	-6.56370	-0.40002	-0.61340
C	-7.63561	-0.91772	0.21031
H	-7.38525	-0.68172	1.25647
H	-7.68676	-2.02051	0.09736
C	-7.13850	-0.25644	-1.93152
H	-7.21471	-1.25076	-2.42075
H	-6.46153	0.38025	-2.52176
C	-8.52978	0.35020	-1.68209
H	-8.45930	1.44863	-1.62664
H	-9.24236	0.09660	-2.48315
C	-8.93607	-0.24416	-0.30052
H	-9.75021	-0.98119	-0.38997
H	-9.27762	0.54658	0.38569

TS(6-B)

SCF (BP86) Energy = -2816.15874495
Enthalpy 0K = -2814.614800
Enthalpy 298K = -2814.511424
Free Energy 298K = -2814.757436
Lowest Frequency = -43.4263 cm^{-1}
Second Frequency = 7.7196 cm^{-1}
SCF (BP86-D3BJ) Energy = -2816.69093841
SCF (C6H6) Energy = -2816.17542808
SCF (BS2) Energy = -4682.01206272

Si	-1.21185	4.04730	2.01187
Si	0.31628	4.65237	-1.53372
Si	0.37978	-4.48649	-1.54513
Si	2.83345	-4.05728	1.48047
Mg	0.21268	1.64063	0.10220
Mg	0.96206	-1.55232	0.03347
Na	-3.66451	-0.83577	0.23658
Na	3.88699	0.84835	-0.38042
N	-1.37861	2.43780	1.26288
N	1.18313	3.19996	-0.98085
N	2.76588	-2.42309	0.77757
N	-0.32621	-3.05482	-0.74452
C	-2.49453	1.68773	1.67070
C	-1.71860	-2.94355	-0.89840
C	2.78883	2.22852	-2.62460
C	-3.80770	1.88123	1.08286
C	2.49946	3.02259	-1.44300
C	-2.39784	0.69903	2.72915
C	3.92565	-1.63548	0.90158
C	-2.30600	-2.21516	-2.00904
C	-2.64497	-3.59114	0.01364
C	4.99719	-1.68625	-0.07634
C	-3.53915	-0.01667	3.14650
H	-3.44163	-0.73338	3.97065
C	-1.09560	0.48877	3.50142
H	-0.31009	1.01898	2.93444
C	2.58634	-5.47961	0.21737
H	3.30762	-5.32255	-0.60816
H	2.95580	-6.38225	0.74952
C	3.63987	3.60231	-0.75694
C	-4.92332	1.15388	1.55419
H	-5.90338	1.33416	1.09814
C	4.12308	2.01604	-3.03467
H	4.31183	1.42231	-3.93664
C	1.17704	-5.75642	-0.34677
H	0.46385	-5.92346	0.48238
H	1.18514	-6.71027	-0.91568
C	-1.35865	5.53117	0.80179
H	-2.28524	5.39035	0.21256
H	-1.57129	6.40119	1.45920
C	-1.43842	-1.49801	-3.04439
H	-0.39619	-1.59102	-2.69140
C	4.10562	-0.71648	2.01193
C	-0.18119	5.87115	-0.13587
H	0.73094	6.07690	0.45761
H	-0.39391	6.82385	-0.66650
C	-3.70423	-2.21788	-2.20777
H	-4.11470	-1.69818	-3.08237
C	-4.80569	0.19869	2.57756
H	-5.68327	-0.34285	2.94746
C	6.12228	-0.84114	0.04458
H	6.91682	-0.90740	-0.70865
C	-2.15290	-4.36920	1.23352
H	-1.06605	-4.18450	1.29054
C	4.95403	3.35523	-1.21061
H	5.79378	3.81192	-0.67281
C	6.26158	0.06245	1.10938
H	7.14525	0.70379	1.18942
C	-4.03467	-3.56602	-0.23618
H	-4.70381	-4.10732	0.44416
C	3.46096	4.52305	0.44933
H	2.37436	4.57966	0.62903
C	1.66651	1.67329	-3.50199
H	0.73521	1.79032	-2.91968
C	4.96696	-2.67179	-1.24386
H	4.02922	-3.24201	-1.13597
C	5.25216	0.10406	2.08424
H	5.36615	0.78031	2.93950
C	-4.57855	-2.90200	-1.34739
H	-5.65616	-2.90926	-1.53304
C	1.52582	-4.26159	2.86253
H	1.79648	-3.66147	3.74798
H	1.44851	-5.31726	3.17984
H	0.52506	-3.93674	2.53312
C	5.21342	2.56099	-2.33824
H	6.23940	2.38731	-2.67853
C	0.46777	4.16811	2.91508
H	0.48018	3.51440	3.80372
H	0.67042	5.20186	3.24855
H	1.29838	3.85988	2.25964
C	-2.57358	4.37543	3.32126
H	-3.51692	4.71073	2.85949
H	-2.23643	5.18056	3.99819
H	-2.80648	3.49124	3.93540
C	1.68599	-3.94684	-2.83127
H	1.20109	-3.47077	-3.70058
H	2.27029	-4.81010	-3.19755
H	2.39505	-3.22090	-2.40233
C	-4.03826	2.89310	-0.04013
H	-3.06665	3.38437	-0.21295
C	-0.93830	-5.49287	-2.50665
H	-1.64571	-6.01362	-1.84097
H	-0.42657	-6.26072	-3.11400
H	-1.53746	-4.86843	-3.18833
C	-1.17850	1.11800	4.91307
H	-1.39048	2.19772	4.87035
H	-0.22615	0.97992	5.45592
H	-1.97560	0.64182	5.51258
C	3.10970	-0.67019	3.17028
H	2.22818	-1.24661	2.83848
C	-1.26097	4.15210	-2.49328
H	-0.99689	3.70781	-3.46830
H	-1.91058	5.02569	-2.68274
H	-1.85164	3.40806	-1.93434
C	-0.69347	-0.99225	3.62296
H	-1.42273	-1.57586	4.21251
H	0.28119	-1.08823	4.13037
H	-0.60160	-1.46802	2.62987
C	-1.53804	-2.15211	-4.44226
H	-1.24344	-3.21312	-4.42130
H	-0.87809	-1.63190	-5.15924
H	-2.56748	-2.09718	-4.84058
C	1.84381	0.18146	-3.84267
H	2.77051	-0.01096	-4.41223
H	1.00565	-0.17648	-4.46511
H	1.86511	-0.43723	-2.92735
C	-1.78257	0.00086	-3.14751
H	-2.83468	0.16293	-3.44439
H	-1.15077	0.50081	-3.90119
H	-1.60789	0.51315	-2.18306
C	1.38434	5.72184	-2.71810
H	1.94305	6.49617	-2.16549
H	0.72891	6.24540	-3.43660
H	2.11686	5.13369	-3.29366

```
C   2.65301  0.75797  3.52249
H   3.48974  1.39151  3.86743
H   1.90885  0.73686  4.33736
H   2.18105  1.25239  2.65437
C   4.54291 -4.43157  2.26877
H   5.23361 -4.87632  1.53251
H   4.42047 -5.16592  3.08461
H   5.03575 -3.53932  2.68644
C   4.94411 -1.95805 -2.61297
H   4.07937 -1.27904 -2.70903
H   4.87805 -2.69201 -3.43525
H   5.86110 -1.36143 -2.77184
C  -2.37694 -5.89260  1.09144
H  -3.45318 -6.12875  1.00366
H  -1.99007 -6.42253  1.97986
H  -1.87000 -6.30467  0.20562
C  -5.07427  3.97709  0.33135
H  -6.07905  3.54239  0.48274
H  -5.15523  4.72412 -0.47794
H  -4.79833  4.50714  1.25653
C  -2.81219 -3.88257  2.54263
H  -2.70155 -2.79454  2.67986
H  -2.35099 -4.37860  3.41469
H  -3.89203 -4.11907  2.56542
C  -4.46649  2.19576 -1.35072
H  -3.69970  1.48217 -1.70011
H  -4.61223  2.93323 -2.15983
H  -5.41512  1.64527 -1.21455
C   4.13287  3.96498  1.72243
H   3.74878  2.96459  1.98565
H   3.94359  4.62761  2.58512
H   5.22840  3.88420  1.59921
C   1.52625  2.49306 -4.80732
H   1.34120  3.55944 -4.60400
H   0.68737  2.11104 -5.41674
H   2.44377  2.42184 -5.41929
C   3.68999 -1.35586  4.43076
H   3.97798 -2.40058  4.23482
H   2.94739 -1.35410  5.24894
H   4.58753 -0.82233  4.79297
C   6.14925 -3.66608 -1.19341
H   7.11613 -3.15135 -1.34072
H   6.05115 -4.42170 -1.99270
H   6.19714 -4.19275 -0.22717
C   3.98207  5.95048  0.16653
H   5.07692  5.95649  0.01554
H   3.76077  6.61771  1.01821
H   3.51984  6.38056 -0.73592
O  -6.83173 -0.51777 -0.66530
C  -7.87530 -1.03550  0.18317
H  -7.61188 -0.78386  1.22276
H  -7.92123 -2.14209  0.08914
C  -7.42074 -0.46480 -1.97845
H  -7.45347 -1.48403 -2.42314
H  -6.77906  0.17348 -2.60589
C  -8.84617  0.08399 -1.75911
H  -8.84646  1.18430 -1.81910
H  -9.55436 -0.29413 -2.51390
C  -9.19319 -0.38765 -0.31395
H  -10.02472 -1.11061 -0.29633
H  -9.48175  0.46613  0.31957
```

C

SCF (BP86) Energy = -2583.20592714
Enthalpy 0K = -2581.778346
Enthalpy 298K = -2581.684152
Free Energy 298K = -2581.914592
Lowest Frequency = 8.1163 cm^{-1}
Second Frequency = 11.9751 cm^{-1}
SCF (BP86-D3BJ) Energy = -2583.67007784
SCF (C6H6) Energy = -2583.21803275
SCF (BS2) Energy = -4124.83557367

```
Si  6.09247 -0.79383 -1.29873
Si  3.09992 -3.63523 -0.16165
Si -6.09255  0.79201 -1.30077
Si -3.10137  3.63540 -0.16422
N   4.71005  0.27323 -0.96933
N   2.50413 -2.18540  0.68774
N  -2.50545  2.18632  0.68630
N  -4.71017 -0.27447 -0.96915
C   4.61886  1.60214 -1.48512
C  -4.61831 -1.60366 -1.48409
C   0.33735 -2.45878  1.89097
C   3.95601  1.85026 -2.73516
C   1.76207 -2.30308  1.90081
C   5.13236  2.72307 -0.75160
C  -1.76250  2.30493  1.89874
C  -5.13262 -2.72419 -0.75054
C  -3.95402 -1.85245 -2.73323
C  -2.42654  2.24896  3.17187
C   4.95353  4.02653 -1.25347
H   5.35112  4.87527 -0.68375
C   5.88572  2.53750  0.56543
H   5.84448  1.45979  0.80316
C  -4.97719  3.51603 -0.58706
H  -5.53865  3.35000  0.35324
H  -5.21315  4.56478 -0.86563
C   2.42686 -2.24559  3.17347
C   3.79133  3.17411 -3.18694
H   3.27501  3.35248 -4.13785
C  -0.36743 -2.56670  3.10596
H  -1.45554 -2.70798  3.08469
C  -5.49308  2.57424 -1.71410
H  -4.76371  2.51462 -2.54542
H  -6.39637  3.03061 -2.17019
C   5.49284 -2.57619 -1.71132
H   4.76402 -2.51690 -2.54314
H   6.39630 -3.03312 -2.16652
C  -5.88730 -2.53791  0.56562
H  -5.84637 -1.46006  0.80273
C  -0.33774  2.46002  1.88784
C   4.97596 -3.51712 -0.58403
H   5.53723 -3.35097  0.35637
H   5.21136 -4.56615 -0.86202
C  -4.95324 -4.02792 -1.25153
H  -5.35146 -4.87636 -0.68179
C   4.28173  4.26466 -2.45840
H   4.14942  5.28650 -2.82954
C  -1.67656  2.34197  4.35808
H  -2.19329  2.29438  5.32331
C  -3.41402 -0.70664 -3.59110
H  -3.79480  0.22811 -3.14234
C   1.67768 -2.33797  4.36024
H   2.19500 -2.28922  5.32510
C  -0.28619  2.50922  4.33665
H   0.27862  2.59226  5.27080
C  -3.78884 -3.17654 -3.18413
H  -3.27142 -3.35541 -4.13434
C   3.94108 -2.05140  3.25052
H   4.34786 -2.36074  2.27050
C  -0.44185 -2.51799  0.57918
H   0.28124 -2.31515 -0.23046
C  -3.94083  2.05575  3.24998
H  -4.34796  2.36428  2.26986
C   0.36788  2.56853  3.10230
```

H	1.45605	2.70925	3.08024
C	-4.28010	-4.26670	-2.45558
H	-4.14739	-5.28873	-2.82602
C	-2.18918	3.90102	-1.82362
H	-1.12958	4.16353	-1.66817
H	-2.65630	4.71520	-2.40579
H	-2.21777	2.99062	-2.44847
C	0.28740	-2.50604	4.33984
H	-0.27676	-2.58865	5.27442
C	7.27024	-0.95087	0.20194
H	7.79523	-0.00250	0.40417
H	8.03442	-1.72821	0.02209
H	6.72405	-1.23089	1.12041
C	7.09523	-0.16212	-2.78949
H	6.54171	-0.29571	-3.73371
H	8.04961	-0.70960	-2.87500
H	7.32582	0.91135	-2.68600
C	-7.27184	0.94992	0.19860
H	-7.79686	0.00163	0.40107
H	-8.03597	1.72701	0.01748
H	-6.72655	1.23070	1.11738
C	3.41706	0.70400	-3.59308
H	3.79703	-0.23054	-3.14317
C	-7.09352	0.15884	-2.79211
H	-6.53894	0.29164	-3.73581
H	-8.04790	0.70605	-2.87920
H	-7.32401	-0.91458	-2.68787
C	7.37263	2.93636	0.42340
H	7.86553	2.37236	-0.38638
H	7.92414	2.74487	1.36144
H	7.47621	4.01063	0.18752
C	0.44058	2.51816	0.57548
H	-0.28312	2.31501	-0.23355
C	2.18809	-3.90115	-1.82119
H	1.12826	-4.16287	-1.66594
H	2.65487	-4.71594	-2.40280
H	2.21749	-2.99110	-2.44650
C	5.23492	3.30779	1.73571
H	5.25104	4.39888	1.56565
H	5.77248	3.11200	2.68046
H	4.17965	3.01531	1.87858
C	-7.37404	-2.93700	0.42239
H	-7.86621	-2.37352	-0.38820
H	-7.92647	-2.74502	1.35977
H	-7.47730	-4.01142	0.18704
C	-1.53390	-1.41071	0.50885
H	-2.27938	-1.57313	1.31388
H	-2.04036	-1.49444	-0.47656
H	-1.06206	-0.41681	0.64835
C	-5.23754	-3.30746	1.73696
H	-5.25337	-4.39865	1.56749
H	-5.77604	-3.11122	2.68108
H	-4.18245	-3.01477	1.88067
C	2.88956	-5.17509	0.93988
H	1.85524	-5.27253	1.30862
H	3.55040	-5.12525	1.82176
H	3.14087	-6.09120	0.37830
C	1.53228	1.41057	0.50502
H	2.27827	1.57337	1.30954
H	2.03816	1.49354	-0.48073
H	1.06033	0.41685	0.64536
C	-2.89182	5.17603	0.93639
H	-3.55341	5.12705	1.81775
H	-3.14246	6.09174	0.37386
H	-1.85777	5.27357	1.30588
C	-4.29455	0.56362	3.45305
H	-3.86199	-0.08231	2.66093
H	-5.38648	0.39908	3.45035
H	-3.88833	0.18862	4.40875
C	-3.91244	-0.77358	-5.05133
H	-3.50850	-1.65389	-5.58221
H	-3.59200	0.12271	-5.61138
H	-5.01233	-0.83464	-5.09858
C	3.91764	0.76998	-5.05262
H	3.51470	1.65011	-5.58456
H	3.59779	-0.12654	-5.61265
H	5.01761	0.83074	-5.09831
C	-1.86956	-0.64529	-3.55980
H	-1.48319	-0.50582	-2.53346
H	-1.49180	0.19115	-4.17423
H	-1.42638	-1.57896	-3.94926
C	1.87255	0.64294	-3.56404
H	1.48462	0.50430	-2.53817
H	1.49557	-0.19388	-4.17842
H	1.43012	1.57639	-3.95486
C	4.29403	-0.55884	3.45181
H	3.86061	0.08599	2.65927
H	5.38586	-0.39366	3.44830
H	3.88814	-0.18308	4.40735
C	-1.08335	-3.90025	0.33376
H	-0.31428	-4.68999	0.32189
H	-1.62607	-3.92741	-0.62730
H	-1.80316	-4.15278	1.13205
C	1.08224	3.90014	0.32884
H	0.31336	4.69007	0.31718
H	1.62425	3.92666	-0.63263
H	1.80272	4.15287	1.12646
C	-4.62569	2.91602	4.33146
H	-4.34219	2.60547	5.35260
H	-5.72328	2.82344	4.25740
H	-4.36281	3.98117	4.22025
C	4.62709	-2.91013	4.33250
H	4.34398	-2.59869	5.35347
H	5.72459	-2.81696	4.25772
H	4.36480	-3.97556	4.22255
Mg	-3.26216	0.48224	0.09808
Mg	3.26101	-0.48215	0.09737

D

SCF (BP86) Energy = -2583.21412018
Enthalpy 0K = -2581.788503
Enthalpy 298K = -2581.692872
Free Energy 298K = -2581.933250
Lowest Frequency = 6.2911 cm^{-1}
Second Frequency = 6.6066 cm^{-1}
SCF (BP86-D3BJ) Energy = -2583.65850152
SCF (C6H6) Energy = -2583.23103716
SCF (BS2) Energy = -4124.84408222

Si	2.23280	2.56103	-0.79708
Si	-2.23256	-2.56026	-0.79748
Si	2.22911	-2.56193	0.79637
Si	-2.22922	2.56329	0.79578
Mg	-3.88217	0.00173	0.00253
Mg	3.88256	-0.00132	0.00126
N	-3.67762	-1.93696	0.02318
N	3.67474	-1.93966	-0.02394
N	-3.67437	1.94008	-0.02460
N	3.67751	1.93734	0.02382
C	-4.70905	-2.78161	0.54466
C	0.69073	2.64381	0.35712
H	0.75617	1.77198	1.03783
H	0.79217	3.53049	1.01298
C	4.70959	2.78150	0.54476
C	-0.69074	-2.64242	0.35710
H	-0.75637	-1.77037	1.03752

H	-0.79222	-3.52889	1.01325
C	-4.70682	2.78411	-0.54491
C	5.83776	3.11733	-0.27638
C	4.70624	-2.78465	-0.54462
C	-0.68706	2.64478	-0.35835
H	-0.75360	1.77327	-1.03936
H	-0.78738	3.53182	-1.01387
C	-4.66566	3.27141	-1.88937
C	-4.66830	-3.26509	1.89054
C	-5.83573	3.11779	0.27614
C	1.84934	-1.26736	2.15946
H	2.67219	-1.21842	2.89377
H	0.92781	-1.51896	2.71139
H	1.69235	-0.24538	1.75876
C	4.66872	3.26675	1.88999
C	-5.83642	-3.11972	-0.27662
C	-2.52198	-4.27068	-1.57733
H	-2.91468	-4.97647	-0.82623
H	-1.57689	-4.68391	-1.96969
H	-3.24552	-4.22624	-2.40783
C	-3.51083	2.92714	-2.82852
H	-2.78943	2.33529	-2.23890
C	1.85206	1.26719	-2.16051
H	2.67508	1.21753	-2.89457
H	0.93093	1.51987	-2.71260
H	1.69404	0.24531	-1.75999
C	-5.93834	-2.59139	-1.70841
H	-4.90662	-2.37543	-2.03836
C	2.51768	-4.27209	1.57713
H	2.90944	-4.97863	0.82624
H	1.57260	-4.68445	1.97041
H	3.24186	-4.22758	2.40709
C	-1.85204	-1.26641	-2.16092
H	-2.67515	-1.21692	-2.89489
H	-0.93090	-1.51894	-2.71306
H	-1.69426	-0.24450	-1.76040
C	5.83428	-3.12075	0.27662
C	0.68720	-2.64349	-0.35810
H	0.75377	-1.77214	-1.03932
H	0.78769	-3.53069	-1.01340
C	-5.93745	2.58556	1.70649
H	-4.90520	2.37342	2.03740
C	5.93960	2.58715	-1.70749
H	4.90766	2.37276	-2.03783
C	2.52311	4.27116	-1.57725
H	2.91483	4.97730	-0.82596
H	1.57854	4.68421	-1.97104
H	3.24776	4.22637	-2.40677
C	6.87501	3.90479	0.25594
H	7.73552	4.15645	-0.37390
C	-1.84903	1.26939	2.15942
H	-2.67247	1.21921	2.89299
H	-0.92841	1.52241	2.71223
H	-1.69001	0.24766	1.75887
C	4.66501	-3.27053	-1.88959
C	6.82908	4.38014	1.57303
H	7.64409	4.99539	1.96883
C	3.51469	2.92001	2.82922
H	2.79256	2.33006	2.23856
C	-5.72875	-4.05553	2.37610
H	-5.69088	-4.41969	3.40998
C	5.72978	4.05669	2.37502
H	5.69179	4.42226	3.40840
C	-2.51896	4.27356	1.57586
H	-2.91058	4.97971	0.82452
H	-1.57434	4.68648	1.96964
H	-3.24367	4.22895	2.40535
C	5.93631	-2.58998	1.70750
H	4.90436	-2.37588	2.03802
C	-5.72709	4.06130	-2.37369
H	-5.68883	4.42851	-3.40648
C	-6.87338	3.90518	-0.25549
H	-7.73439	4.15524	0.37429
C	-6.87303	-3.90768	0.25622
H	-7.73296	-4.16108	-0.37372
C	3.51119	-2.92351	-2.82897
H	2.78970	-2.33238	-2.23874
C	-3.51518	-2.91574	2.82992
H	-2.79286	-2.32672	2.23856
C	-2.78333	4.18750	-3.34563
H	-3.45101	4.81743	-3.96001
H	-1.91824	3.91063	-3.97424
H	-2.41685	4.81097	-2.51236
C	-6.82710	4.38261	-1.57182
H	-7.64239	4.99782	-1.96710
C	-6.82726	-4.38126	1.57396
H	-7.64180	-4.99690	1.97014
C	-6.71984	-1.25756	-1.75140
H	-7.75537	-1.39250	-1.39329
H	-6.75852	-0.83991	-2.77304
H	-6.26849	-0.48259	-1.09749
C	6.55483	3.58962	-2.70456
H	6.03222	4.56005	-2.66934
H	6.48523	3.19795	-3.73446
H	7.62419	3.77666	-2.50196
C	-6.71331	1.24830	1.74534
H	-7.74918	1.37981	1.38696
H	-6.75070	0.82720	2.76560
H	-6.25915	0.47668	1.08912
C	-6.55606	3.58504	2.70444
H	-6.03594	4.55685	2.67087
H	-6.48616	3.19219	3.73388
H	-7.62576	3.76953	2.50132
C	6.71870	1.25185	-1.74854
H	7.75434	1.38535	-1.39023
H	6.75693	0.83267	-2.76957
H	6.26602	0.47839	-1.09366
C	6.87103	-3.90911	-0.25533
H	7.73140	-4.16102	0.37459
C	-3.98502	2.04833	-4.00862
H	-4.47028	1.12258	-3.65203
H	-3.13569	1.76193	-4.65427
H	-4.71971	2.58144	-4.63778
C	6.55219	-3.59172	2.70488
H	7.62159	-3.77839	2.50215
H	6.02998	-4.56238	2.67019
H	6.48263	-3.19963	3.73463
C	3.98999	2.03754	4.00616
H	4.72561	2.56861	4.63597
H	4.47469	1.11275	3.64625
H	3.14140	1.74934	4.65198
C	5.72555	-4.06141	-2.37425
H	5.68726	-4.42749	-3.40744
C	6.71497	-1.25440	1.74789
H	6.75297	-0.83454	2.76864
H	6.26235	-0.48132	1.09243
H	7.75068	-1.38775	1.38971
C	6.82470	-4.38513	-1.57217
H	7.63929	-5.00111	-1.96770
C	-6.55107	-3.59623	-2.70462
H	-6.02663	-4.56563	-2.66799
H	-6.48166	-3.20564	-3.73495
H	-7.62018	-3.78510	-2.50240
C	2.78781	4.17883	3.35086
H	1.92328	3.90008	3.97940
H	2.42062	4.80483	2.51982

H	3.45603	4.80686	3.96659
C	-3.99198	-2.03090	4.00449
H	-4.72793	-2.56097	4.63475
H	-4.47688	-1.10716	3.64208
H	-3.14415	-1.74079	4.65045
C	3.98715	-2.04249	-4.00673
H	4.47273	-1.11784	-3.64767
H	3.13871	-1.75408	-4.65265
H	4.72216	-2.57478	-4.63622
C	2.78313	-4.18213	-3.34946
H	3.45072	-4.81123	-3.96477
H	1.91874	-3.90314	-3.97808
H	2.41553	-4.80715	-2.51786
C	-2.78798	-4.17295	3.35495
H	-1.92409	-3.89225	3.98350
H	-2.41978	-4.80057	2.52559
H	-3.45625	-4.80001	3.97161

E

SCF (BP86) Energy = -2583.43445009
Enthalpy 0K = -2582.006425
Enthalpy 298K = -2581.910640
Free Energy 298K = -2582.140527
Lowest Frequency = 13.8228 cm^{-1}
Second Frequency = 14.3690 cm^{-1}
SCF (BP86-D3BJ) Energy = -2583.92378496
SCF (C6H6) Energy = -2583.45206713
SCF (BS2) Energy = -4287.14679731

Si	4.51983	1.14549	1.93217
Si	4.96351	-1.25893	-1.16298
Si	-4.52104	1.14285	-1.93198
Si	-4.96236	-1.26157	1.16344
Mg	2.24323	0.09747	-0.03113
Mg	-2.24313	0.09653	0.03097
Na	0.00151	-3.42916	0.00029
N	3.18036	1.61396	0.85855
N	3.23886	-1.51458	-0.73675
N	-3.23760	-1.51609	0.73696
N	-3.18179	1.61223	-0.85850
C	2.73888	2.97367	0.88855
C	-2.74106	2.97218	-0.88862
C	1.83635	-2.88743	-2.26407
C	3.39776	3.99168	0.11804
C	2.63356	-2.74205	-1.06345
C	1.61495	3.36340	1.69309
C	-2.63158	-2.74327	1.06336
C	-1.61737	3.36250	-1.69321
C	-3.40048	3.98988	-0.11815
C	-2.76885	-3.90956	0.21493
C	1.19508	4.70700	1.71610
H	0.33475	4.98703	2.33561
C	0.84398	2.35193	2.54039
H	1.29194	1.35995	2.34061
C	-6.08909	-0.85750	-0.33960
H	-5.89983	-1.62572	-1.11455
H	-7.11013	-1.08465	0.03506
C	2.77158	-3.90846	-0.21529
C	2.94081	5.32191	0.17831
H	3.45860	6.08678	-0.41374
C	1.21773	-4.12082	-2.56164
H	0.63307	-4.21150	-3.48446
C	-6.08670	0.55355	-0.98037
H	-6.32466	1.31781	-0.21675
H	-6.91622	0.61496	-1.71544
C	6.08608	0.55734	0.98079
H	6.32354	1.32168	0.21709
H	6.91547	0.61949	1.71595
C	-0.84587	2.35146	-2.54053
H	-1.29312	1.35918	-2.34057
C	-1.83436	-2.88846	2.26401
C	6.08964	-0.85381	0.34022
H	5.90083	-1.62205	1.11527
H	7.11093	-1.08024	-0.03421
C	-1.19824	4.70633	-1.71628
H	-0.33807	4.98681	-2.33582
C	1.84949	5.69281	0.97097
H	1.51053	6.73344	1.00424
C	-2.13563	-5.12242	0.56527
H	-2.26632	-5.99605	-0.08423
C	-4.59100	3.67563	0.78914
H	-4.79361	2.59735	0.67549
C	2.13897	-5.12158	-0.56581
H	2.27019	-5.99528	0.08349
C	-1.35643	-5.24339	1.72814
H	-0.88579	-6.19688	1.98892
C	-2.94427	5.32035	-0.17849
H	-3.46247	6.08496	0.41354
C	3.58628	-3.85943	1.07779
H	4.11617	-2.89233	1.07357
C	1.72258	-1.75067	-3.27961
H	2.18413	-0.86523	-2.80531
C	-3.58351	-3.86075	-1.07818
H	-4.11370	-2.89380	-1.07391
C	-1.21514	-4.12158	2.56142
H	-0.63054	-4.21214	3.48429
C	-1.85317	5.69182	-0.97118
H	-1.51478	6.73263	-1.00450
C	-5.10765	0.14345	2.44535
H	-4.68315	-0.16504	3.41587
H	-6.16397	0.42107	2.61066
H	-4.57673	1.05132	2.11437
C	1.35969	-5.24271	-1.72860
H	0.88949	-6.19638	-1.98950
C	3.95973	-0.30671	3.04797
H	3.17225	0.02105	3.74794
H	4.80114	-0.70591	3.64274
H	3.55740	-1.14425	2.45089
C	5.04522	2.57925	3.07227
H	5.45364	3.43282	2.50821
H	5.81988	2.23490	3.77947
H	4.19018	2.95594	3.65730
C	-3.96010	-0.30873	-3.04816
H	-3.17308	0.01983	-3.74828
H	-4.80136	-0.70845	-3.64279
H	-3.55691	-1.14610	-2.45142
C	4.58849	3.67803	-0.78917
H	4.79157	2.59984	-0.67555
C	-5.04754	2.57637	-3.07188
H	-5.45642	3.42963	-2.50769
H	-5.82206	2.23163	-3.77904
H	-4.19278	2.95364	-3.65694
C	0.98414	2.63027	4.05362
H	2.04027	2.60531	4.36954
H	0.43209	1.87976	4.64848
H	0.58191	3.62513	4.31473
C	-1.72116	-1.75178	3.27970
H	-2.18333	-0.86657	2.80558
C	5.10809	0.14603	-2.44504
H	4.68397	-0.16286	-3.41560
H	6.16427	0.42432	-2.61021
H	4.57653	1.05361	-2.11430
C	-0.64678	2.29679	2.14454
H	-1.16531	3.24562	2.36219
H	-1.17130	1.49288	2.69666
H	-0.76084	2.13460	1.05458

C -0.98654 2.62953 -4.05377
H -2.04272 2.60384 -4.36947
H -0.43411 1.87932 -4.64866
H -0.58503 3.62464 -4.31505
C   0.26626 -1.40196 -3.64499
H -0.25152 -2.24452 -4.13699
H   0.23607 -0.54801 -4.34359
H -0.31754 -1.11909 -2.74961
C   0.64501 2.29734 -2.14499
H   1.16288 3.24647 -2.36291
H   1.16992 1.49369 -2.69712
H   0.75943 2.13535 -1.05505
C   5.69060 -2.83732 -1.95589
H   5.86740 -3.63326 -1.21424
H   6.66193 -2.59669 -2.42235
H   5.03311 -3.25009 -2.73881
C -0.26496 -1.40225 3.64481
H   0.25342 -2.24457 4.13658
H -0.23510 -0.54835 4.34349
H   0.31845 -1.11892 2.74931
C -5.68818 -2.84042 1.95663
H -5.86453 -3.63653 1.21506
H -6.65959 -2.60049 2.42327
H -5.03022 -3.25268 2.73943
C -2.66357 -3.88359 -2.31983
H -1.95149 -3.03857 -2.31838
H -3.25324 -3.80751 -3.25005
H -2.08161 -4.82255 -2.36730
C -5.86746 4.44767 0.38537
H -5.72601 5.53891 0.48262
H -6.71562 4.16463 1.03454
H -6.15732 4.24370 -0.65857
C   5.86455 4.45063 -0.38521
H   5.72258 5.54181 -0.48235
H   6.71291 4.16806 -1.03432
H   6.15440 4.24668 0.65874
C -4.26874 3.94328 2.27719
H -3.40042 3.35396 2.61615
H -5.12917 3.68038 2.91843
H -4.03546 5.00889 2.45174
C   4.26626 3.94562 -2.27724
H   3.39821 3.35597 -2.61630
H   5.12686 3.68307 -2.91840
H   4.03259 5.01115 -2.45179
C   2.66637 -3.88258 2.31947
H   1.95408 -3.03774 2.31809
H   3.25602 -3.80639 3.24969
H   2.08466 -4.82170 2.36689
C   2.53356 -2.07234 -4.55727
H   3.59042 -2.28205 -4.32467
H   2.49954 -1.22303 -5.26195
H   2.12275 -2.95705 -5.07609
C -2.53168 -2.07408 4.55749
H -3.58847 -2.28432 4.32509
H -2.49797 -1.22484 5.26228
H -2.12028 -2.95863 5.07611
C -4.62937 -4.99239 -1.17934
H -4.15297 -5.98430 -1.27992
H -5.26565 -4.84455 -2.06900
H -5.28350 -5.02637 -0.29313
C   4.63248 -4.99077 1.17892
H   4.15637 -5.98282 1.27965
H   5.26882 -4.84268 2.06848
H   5.28651 -5.02464 0.29263

F

SCF (BP86) Energy = -2583.51442294
Enthalpy 0K = -2582.087047
Enthalpy 298K = -2581.991969
Free Energy 298K = -2582.215237
Lowest Frequency = 22.0906 cm^{-1}
Second Frequency = 28.1536 cm^{-1}
SCF (BP86-D3BJ) Energy = -2584.00913155
SCF (C6H6) Energy = -2583.54967010
SCF (BS2) Energy = -4287.23357084

Si 4.39642 1.13372 1.63281
Si 4.45586 -0.91945 -1.74125
Si -4.39540 1.13591 -1.63298
Si -4.45656 -0.91780 1.74076
Mg 1.62790 0.04648 0.00979
Mg -1.62772 0.04702 -0.00970
Na 0.00051 -3.22063 0.00231
N   2.77882 1.48788 1.00596
N   2.89913 -1.38066 -1.02857
N -2.89998 -1.37960 1.02813
N -2.77773 1.48929 -1.00581
C   2.30059 2.78573 1.36565
C -2.29901 2.78703 -1.36522
C   1.52855 -2.96434 -2.38480
C   2.57896 3.94063 0.55165
C   2.41732 -2.67389 -1.27146
C   1.56054 3.00430 2.58116
C -2.41884 -2.67309 1.27090
C -1.55878 3.00552 -2.58066
C -2.57697 3.94190 -0.55106
C -2.74476 -3.78935 0.40046
C   1.18193 4.30788 2.95745
H   0.63632 4.44994 3.89934
C   1.17421 1.85316 3.51095
H   1.51809 0.92565 3.01911
C -5.82336 -0.47661 0.46770
H -5.89412 -1.32101 -0.24643
H -6.76506 -0.52401 1.05781
C   2.74329 -3.79051 -0.40155
C   2.17308 5.22196 0.96870
H   2.40763 6.08454 0.33198
C   0.99779 -4.26063 -2.55876
H   0.33535 -4.45011 -3.41178
C -5.75214 0.86484 -0.29166
H -5.67035 1.70344 0.42607
H -6.71040 1.03648 -0.82749
C   5.75273 0.86238 0.29112
H   5.67109 1.70111 -0.42646
H   6.71122 1.03352 0.82669
C -1.17303 1.85439 -3.51070
H -1.51757 0.92697 -3.01915
C -1.53094 -2.96427 2.38477
C   5.82315 -0.47900 -0.46845
H   5.89379 -1.32352 0.24556
H   6.76465 -0.52672 -1.05885
C -1.17953 4.30900 -2.95665
H -0.63378 4.45099 -3.89847
C   1.48783 5.42356 2.17090
H   1.18658 6.42963 2.48386
C -2.17970 -5.06417 0.61700
H -2.44787 -5.88351 -0.06181
C -3.32290 3.82254 0.77678
H -3.47991 2.74137 0.93793
C   2.17748 -5.06503 -0.61804
H   2.44575 -5.88469 0.06036
C -1.30263 -5.31579 1.68311
H -0.87478 -6.31222 1.83619
C -2.17047 5.22312 -0.96782
H -2.40473 6.08570 -0.33097
C   3.74419 -3.63230 0.74087

H	4.00899	-2.56146	0.76056
C	1.20058	-1.90058	-3.43204
H	1.53004	-0.93753	-3.00445
C	-3.74527	-3.63045	-0.74216
H	-4.00902	-2.55935	-0.76216
C	-1.00103	-4.26093	2.55884
H	-0.33938	-4.45100	3.41234
C	-1.48499	5.42466	-2.16989
H	-1.18324	6.43066	-2.48261
C	-4.26032	0.56532	2.93196
H	-3.68453	0.27560	3.82756
H	-5.24654	0.93661	3.26573
H	-3.73230	1.40649	2.45359
C	1.29950	-5.31589	-1.68357
H	0.87099	-6.31205	-1.83658
C	4.38384	-0.40716	2.77565
H	3.90110	-0.16007	3.73700
H	5.40650	-0.76873	2.98958
H	3.81878	-1.23977	2.32708
C	5.09814	2.56475	2.70091
H	5.33610	3.45579	2.09698
H	6.02822	2.23285	3.19731
H	4.38782	2.88688	3.47887
C	-4.38324	-0.40462	-2.77631
H	-3.90077	-0.15722	-3.73772
H	-5.40598	-0.76604	-2.99010
H	-3.81811	-1.23745	-2.32824
C	3.32462	3.82119	-0.77633
H	3.48102	2.73998	-0.93779
C	-5.09645	2.56752	-2.70076
H	-5.33444	3.45834	-2.09652
H	-6.02643	2.23602	-3.19759
H	-4.38581	2.88985	-3.47834
C	1.86425	1.95016	4.89103
H	2.96291	1.95375	4.79929
H	1.58017	1.09583	5.53384
H	1.57096	2.87504	5.42096
C	-1.20290	-1.90093	3.43244
H	-1.53207	-0.93766	3.00512
C	4.26009	0.56394	-2.93219
H	3.68387	0.27471	-3.82767
H	5.24643	0.93473	-3.26616
H	3.73270	1.40533	-2.45352
C	-0.35521	1.76728	3.69714
H	-0.76586	2.69551	4.13219
H	-0.62885	0.93715	4.37265
H	-0.86130	1.60034	2.73034
C	-1.86278	1.95220	-4.89087
H	-2.96145	1.95651	-4.79931
H	-1.57917	1.09782	-5.53383
H	-1.56877	2.87700	-5.42053
C	-0.30383	-1.80088	-3.74773
H	-0.70837	-2.74223	-4.16233
H	-0.49131	-1.01165	-4.49536
H	-0.88230	-1.54024	-2.84333
C	0.35638	1.76758	-3.69667
H	0.76768	2.69565	-4.13143
H	0.62958	0.93743	-4.37234
H	0.86220	1.60007	-2.72983
C	5.23232	-2.35814	-2.76339
H	5.98751	-2.90270	-2.17011
H	5.74802	-1.95389	-3.65288
H	4.48798	-3.09411	-3.10823
C	0.30155	-1.80171	3.74817
H	0.70594	-2.74343	4.16209
H	0.48926	-1.01302	4.49633
H	0.88000	-1.54050	2.84391
C	-5.23378	-2.35627	2.76265
H	-5.99006	-2.89965	2.16966
H	-5.74831	-1.95200	3.65280
H	-4.48998	-3.09327	3.10644
C	-3.15611	-4.01341	-2.11596
H	-2.25529	-3.42344	-2.35472
H	-3.89275	-3.83044	-2.91822
H	-2.87866	-5.08318	-2.15599
C	-4.70901	4.50575	0.74323
H	-4.61077	5.58949	0.54728
H	-5.22710	4.38768	1.71310
H	-5.35819	4.08297	-0.04103
C	4.71113	4.50363	-0.74280
H	4.61351	5.58736	-0.54652
H	5.22899	4.38555	-1.71280
H	5.36020	4.08024	0.04122
C	-2.49714	4.38401	1.95419
H	-1.48413	3.95391	1.98465
H	-2.99757	4.17201	2.91735
H	-2.38278	5.48080	1.87642
C	2.49902	4.38344	-1.95347
H	1.48574	3.95398	-1.98387
H	2.99916	4.17135	-2.91676
H	2.38535	5.48028	-1.87543
C	3.15477	-4.01426	2.11484
H	2.25485	-3.42295	2.35382
H	3.89180	-3.83222	2.91696
H	2.87581	-5.08363	2.15505
C	1.99247	-2.14644	-4.73880
H	3.07926	-2.16911	-4.56147
H	1.78224	-1.34827	-5.47364
H	1.70574	-3.11063	-5.19842
C	-1.99495	-2.14702	4.73905
H	-3.08172	-2.16947	4.56158
H	-1.78467	-1.34907	5.47412
H	-1.70844	-3.11137	5.19846
C	-5.03496	-4.44158	-0.48034
H	-4.82801	-5.52801	-0.47042
H	-5.78140	-4.24984	-1.27206
H	-5.48695	-4.17925	0.48931
C	5.03301	-4.44483	0.47920
H	4.82503	-5.53106	0.46980
H	5.77978	-4.25347	1.27071
H	5.48510	-4.18338	-0.49065

Neutral Sodium Mirror Oligomers

^{2}Na (*neutral doublet*)
SCF (BP86) Energy = -0.190251935461
Enthalpy 0K = -0.190252
Enthalpy 298K = -0.187891
Free Energy 298K = -0.205335
SCF (BP86-D3BJ) Energy = -0.190251935461
SCF (C6H6) Energy = -0.194926402159
SCF (BS2) Energy = -162.278274773

Na 0.10000 0.10000 0.10000

^{1}Na$_2$ (*neutral singlet*)
SCF (BP86) Energy = -0.402634016756
Enthalpy 0K = -0.402297
Enthalpy 298K = -0.398345
Free Energy 298K = -0.424572
Lowest Frequency = 147.9295 cm^{-1}
SCF (BP86-D3BJ) Energy = -0.404383134369
SCF (C6H6) Energy = -0.408508331967
SCF (BS2) Energy = -324.582566453

Na 0.00000 -0.00000 1.61056
Na 0.00000 -0.00000 -1.61056

^{3}Na$_2$ (*neutral triplet*)
SCF (BP86) Energy = -0.380701750018
Enthalpy 0K = -0.380642
Enthalpy 298K = -0.376452
Free Energy 298K = -0.406164
Lowest Frequency = 26.2787 cm^{-1}
SCF (BP86-D3BJ) Energy = -0.387174366222
SCF (C6H6) Energy = -0.388015591562
SCF (BS2) Energy = -324.556997547

Na 0.00000 0.00000 2.50746
Na 0.00000 0.00000 -2.50746

^{1}Na$_4$ (*neutral singlet*)
SCF (BP86) Energy = -0.816046725385
Enthalpy 0K = -0.814952
Enthalpy 298K = -0.806510
Free Energy 298K = -0.851016
Lowest Frequency = 12.1387 cm^{-1}
Second Frequency = 37.3753 cm^{-1}
SCF (BP86-D3BJ) Energy = -0.823760404012
SCF (C6H6) Energy = -0.826417681689
SCF (BS2) Energy = -649.176337063

Na 1.40049 -3.00310 -0.00000
Na 1.40049 0.81868 -0.00000
Na -1.40049 -0.81868 -0.00000
Na -1.40049 3.00310 -0.00000

^{5}Na$_4$ (*neutral quintet*)
SCF (BP86) Energy = -0.767741060859
Enthalpy 0K = -0.766950
Enthalpy 298K = -0.758262
Free Energy 298K = -0.804817
Lowest Frequency = 52.3943 cm^{-1}
Second Frequency = 52.4052 cm^{-1}
SCF (BP86-D3BJ) Energy = -0.801754694495
SCF (C6H6) Energy = -0.777878298195
SCF (BS2) Energy = -649.122910198

Na 1.48310 1.48310 1.48325
Na 1.48310 -1.48337 -1.48325
Na -1.48310 1.48337 -1.48325
Na -1.48310 -1.48310 1.48325

^{1}Na$_8$ (*neutral singlet*)
SCF (BP86) Energy = -1.66027131451
Enthalpy 0K = -1.657526
Enthalpy 298K = -1.639292
Free Energy 298K = -1.716309
Lowest Frequency = 6.5422 cm^{-1}
Second Frequency = 13.7152 cm^{-1}
SCF (BP86-D3BJ) Energy = -1.68155894147
SCF (C6H6) Energy = -1.67254018286
SCF (BS2) Energy = -1298.38094373

Na 2.91399 4.74075 -0.40935
Na 0.92499 1.55995 -0.18981
Na -1.28990 0.64372 -2.79137
Na -3.17976 -2.65903 -2.27781
Na 3.17976 2.65903 2.27781
Na 1.28990 -0.64372 2.79137
Na -0.92499 -1.55995 0.18981
Na -2.91399 -4.74075 0.40935

^{9}Na$_8$ (*neutral nonet*)
SCF (BP86) Energy = -1.53843918409
Enthalpy 0K = -1.536705
Enthalpy 298K = -1.517596
Free Energy 298K = -1.602564
Lowest Frequency = 6.1091 cm^{-1}
Second Frequency = 11.4594 cm^{-1}
SCF (BP86-D3BJ) Energy = -1.61303157093
SCF (C6H6) Energy = -1.55281745207
SCF (BS2) Energy = -1298.25032202

Na 2.50748 5.02590 -1.31007
Na 2.83141 0.96912 -2.46694
Na -0.86000 2.78776 -2.56696
Na -0.80851 -1.71731 -1.09160
Na 0.80851 1.71731 1.09160
Na 0.86000 -2.78776 2.56696
Na -2.83141 -0.96912 2.46694
Na -2.50748 -5.02590 1.31007

^{2}Na$_9$ (*neutral doublet*)
SCF (BP86) Energy = -1.87231816286
Enthalpy 0K = -1.869267
Enthalpy 298K = -1.848499
Free Energy 298K = -1.933510
Lowest Frequency = 9.3231 cm^{-1}
Second Frequency = 16.4348 cm^{-1}
SCF (BP86-D3BJ) Energy = -1.89812654960
SCF (C6H6) Energy = -1.88436148435
SCF (BS2) Energy = -1460.68299993

Na 3.30072 1.98636 -0.00024
Na -0.00159 -0.00080 0.00006
Na -0.00064 3.54293 -0.00155
Na -3.30226 1.98792 0.00019
Na -3.30145 -1.98816 -0.00067
Na -6.17659 -0.00021 0.00176
Na 3.30172 -1.98553 -0.00026
Na 0.00196 -3.54380 -0.00106
Na 6.17812 0.00129 0.00177

^{10}Na$_9$ (*neutral 10-tet*)
SCF (BP86) Energy = -1.73663859520
Enthalpy 0K = -1.734152

Enthalpy 298K = -1.712920
Free Energy 298K = -1.799419
Lowest Frequency = 28.5818 cm^{-1}
Second Frequency = 28.6202 cm^{-1}
SCF (BP86-D3BJ) Energy = -1.79413440808
SCF (C6H6) Energy = -1.75089668151
SCF (BS2) Energy = -1460.53948761

```
Na  1.53059 -1.95862 -2.16296
Na -2.46284 -0.34118 -2.16498
Na -1.52626 -3.77111 -0.00376
Na -2.46554 -0.34829  2.16293
Na -2.50299  3.20709  0.00327
Na  0.93366  2.30316  2.16361
Na  1.53027 -1.96326  2.16118
Na  0.93318  2.30897 -2.15978
Na  4.02993  0.56323  0.00050
```
